# Epigenetic Switch Driven by DNA Inversions Dictates Phase Variation in *Streptococcus pneumoniae*

**DOI:** 10.1371/journal.ppat.1005762

**Published:** 2016-07-18

**Authors:** Jing Li, Jing-Wen Li, Zhixing Feng, Juanjuan Wang, Haoran An, Yanni Liu, Yang Wang, Kailing Wang, Xuegong Zhang, Zhun Miao, Wenbo Liang, Robert Sebra, Guilin Wang, Wen-Ching Wang, Jing-Ren Zhang

**Affiliations:** 1 Center for Infectious Disease Research, School of Medicine, Tsinghua University, Beijing, China; 2 Department of Genetics and Genomic Sciences, Icahn Institute of Genomics and Multiscale Biology, Icahn School of Medicine at Mount Sinai, New York, New York, United States of America; 3 MOE Key Laboratory of Bioinformatics, Bioinformatics Division, TNLIST and Department of Automation, Tsinghua University, Beijing, China; 4 College of Veterinary Medicine, Gansu Agricultural University, Lanzhou, Gansu, China; 5 W. M. Keck Foundation Biotechnology Resource Laboratory, Yale University, New Haven, Connecticut, United States of America; 6 Biomedical Science and Engineering Center, Institute of Molecular and Cellular Biology, National Tsing Hua University, Hsinchu, Taiwan; 7 Collaborative Innovation Center for Biotherapy, State Key Laboratory of Biotherapy and Center, West China Hospital, West China Medical School, Sichuan University, Chengdu, Sichuan, China; Children's Hospital Boston, UNITED STATES

## Abstract

DNA methylation is an important epigenetic mechanism for phenotypic diversification in all forms of life. We previously described remarkable cell-to-cell heterogeneity in epigenetic pattern within a clonal population of *Streptococcus pneumoniae*, a leading human pathogen. We here report that the epigenetic diversity is caused by extensive DNA inversions among *hsdS*_*A*,_ *hsdS*_*B*,_ and *hsdS*_*C*_, three methyltransferase *hsdS* genes in the Spn556II type-I restriction modification (R-M) locus. Because *hsdS*_*A*_ encodes the sequence recognition subunit of this type-I R-M DNA methyltransferase, these site-specific recombinations generate pneumococcal cells with variable HsdS_A_ alleles and thereby diverse genome methylation patterns. Most importantly, the DNA methylation pattern specified by the HsdS_A1_ allele leads to the formation of opaque colonies, whereas the pneumococci lacking HsdS_A1_ produce transparent colonies. Furthermore, this HsdS_A1_-dependent phase variation requires intact DNA methylase activity encoded by *hsdM* in the Spn556II (renamed colony opacity determinant or *cod*) locus. Thus, the DNA inversion-driven ON/OFF switch of the *hsdS*_*A1*_ allele in the *cod* locus and resulting epigenetic switch dictate the phase variation between the opaque and transparent phenotypes. Phase variation has been well documented for its importance in pneumococcal carriage and invasive infection, but its molecular basis remains unclear. Our work has discovered a novel epigenetic cause for this significant pathobiology phenomenon in *S*. *pneumoniae*. Lastly, our findings broadly represents a significant advancement in our understanding of bacterial R-M systems and their potential in shaping epigenetic and phenotypic diversity of the prokaryotic organisms because similar site-specific recombination systems widely exist in many archaeal and bacterial species.

## Author Summary

DNA methylation is a well-known epigenetic mechanism for phenotypic diversification in all forms of life. This study reports our discovery that the Spn556II type-I RM locus in human pathogen *Streptococcus pneumoniae* undergoes extensive DNA inversions among three homologous DNA methyltransferase genes. These site-specific recombinations generate subpopulations of progeny cells with dramatic epigenetic and phenotypic differences. This is exemplified by the striking differences in colony morphology among the pneumococcal variants that carried different allelic variants of the methyltransferase genes. Phase variation has been well documented for its importance in pneumococcal pathogenesis, but it is currently unknown how this phenotypic switch occurs at the molecular level. This work has thus discovered an epigenetic cause for pneumococcal phase variation. Our findings have a broad implication on the epigenetic and phenotypic diversification in prokaryotic organisms because similar DNA rearrangement systems also exist in many archaeal and bacterial species.

## Introduction

DNA methylation has been demonstrated as an epigenetic means of regulating many important biological processes in both prokaryotic and eukaryotic organisms. Cytosine methylation in the CpG dinucleotide context is essential for shaping embryonic cells into different cell types of the mammals; mutations in DNA methylation-associated genes lead to embryonic death in mice [1–3]. In prokaryotes, DNA methylation is catalyzed by solitary methyltransferases and those associated with restriction-modification (R-M) systems. Examples of the former include the N6-adenine methyltransferases Dam and CcrM, and the C5-cytosine methyltransferase Dcm [4]. As the best characterized DNA methyltransferase in bacteria, the Dam methylase (recognizing a 5’-GATC-3’ sequence) is involved in multiple functions in *Escherichia coli*, such as chromosomal replication, DNA repair, and regulation of transposition [5]. Dam is also responsible for the ON/OFF reversible phase variation of the pyelonephritis-associated pilus (Pap) [6, 7] and antigen 43 (Ag43) [8, 9]. The transcription of *pap* and *agn43* is regulated by the methylation status of multiple GATC sequences in the promoter regions of the *pap* and *agn43* loci. Cell-to-cell variations in the methylation status at the GATC sites by Dam result in ON/OFF production of the Pap pili and Ag43 antigen in a clonal population [10].

The vast majority of DNA methyltransferases in prokaryotic organisms are associated with ubiquitous R-M systems, which are currently recognized as a defense mechanism against invasion of foreign DNA, particularly bacteriophages [11]. The R-M systems are currently divided into four types; each of them typically contains two basic functional units: endonucleases and cognate DNA methyltransferases. As exemplified by the restriction enzymes commonly used in DNA cloning (e.g., BamHI and EcoRI), a typical type-II R-M system consists of a DNA endonuclease (HsdR) and a methyltransferase (HsdM). The former can independently cleave (or restrict) DNA molecules at the specific sequence sites unless the sites are methylated by its partner DNA methyltransferase. In contrast, the type-I R-M system contain three subunits: HsdR, HsdM, and HsdS [12]. HsdS (sequence specificity protein) is responsible for sequence recognition function of both the HsdR and HsdM activities in each type-I R-M system because neither HsdR nor HsdM is capable of sequence recognition. Typical HsdS proteins comprise two unique target recognition domains (TRDs), each of which recognizes one half of the type-I recognition sequence. The *hsdS* genes undergo DNA inversions catalyzed by the HvsR tyrosine recombinase in *Mycoplasma pulmonis*, a respiratory pathogen of rodents [13–16]. While some of the DNA inversions between the *hsdS* genes in *M*. *pulmonis* generate polymorphic HsdS protein variants that recognize unique DNA sequences and thus possess different restriction activities, the other DNA rearrangements lead to the loss of the R-M activities [17]. Although producing the loss-of-function variants of HsdS does not align well with the current paradigm of the type-I R-M systems as a defense mechanism against invasion by foreign DNA [17], the biologic significance of these *hsdS* recombinations remains unclear.

*Streptococcus pneumoniae* (pneumococcus), is a major human pathogen worldwide and responsible for death of approximately 1 million annually [18, 19]. Phenotypic plasticity of *S*. *pneumoniae* is the major driving mechanism behind the success of this pathogen in its adaptation to the increasingly hostile environment in humans, the only known natural host [20, 21]. These include strain-to-strain antigenic variations in the polysaccharide capsule and major surface proteins [21, 22], shuffling of virulence factors [23], and development of resistance to antibiotics [24]. These adaptive traits are predominantly realized by horizontal gene transfer through natural genetic transformation [25]. In addition, genetic diversification in *S*. *pneumoniae* can be also achieved by intra-genomic recombinations in the *hsdS* genes of the type-I RM systems [26–28]. The site-specific DNA rearrangements result in “extra DNA fragments” during shotgun sequencing and assembly of the pneumococcal genome [26], inter-genomic recombinations [27], and programmed variations in genome DNA methylation pattern [27, 28].

*S*. *pneumoniae* is capable of spontaneous and reversible switch between the opaque and transparent colony forms on transparent agar plates, so called phase variation [29]. The opaque and transparent variants are distinct in multiple pathogenesis-associated characteristics, such as the amounts of polysaccharide capsule (higher in the opaque) and cell wall teichoic acids (more in the transparent), autolysis (faster in the transparent), adherence to host epithelial cells (higher in the transparent), and evasion of opsonophagocytic killing (greater in the opaque) [30–33]. These *in vitro* phenotypic differences are correlated with the pneumococcal behaviors in animal models. The opaque variants are more virulent in systemic infection but deficient in nasopharyngeal colonization; the transparent variants display higher levels of nasopharyngeal colonization with relatively lower virulence [30, 33]. Although the box elements (repeat sequences in the pneumococcal genome) influence the frequency of phase variation [34], the molecular basis of this pneumococcal adaptation mechanism remains to be defined.

Our recent study has identified two functional type-I R-M systems (Spn556II and Spn556III) in the multi-drug resistant type 19F strain ST556 of *S*. *pneumoniae* [35]. One of the intriguing findings is the extensive cell-to-cell diversity in the DNA methylation patterns of the genome (or methylomes) in a clonal population. This work revealed that the intercellular heterogeneity of DNA modifications is caused by site-specific recombinations in the three methyltransferase *hsdS* genes of the Spn556II locus. Extensive DNA excisions and inversions of the *hsdS* genes generate bacterial subpopulations with distinct methylomes and phase variation in colony morphology. Further investigation revealed that ON/OFF inversions among the three *hsdS* genes dictate the phase variation through reversible switch of genome methylation pattern (or epigenetic status). These findings have not only provided an epigenetic mechanism for the pneumococcal phase variation, but also identified a novel function for a bacterial type-I R-M system far beyond its known role in the R-M activity.

## Results

### DNA rearrangements occur in the Spn556II locus

**Fig 1:**
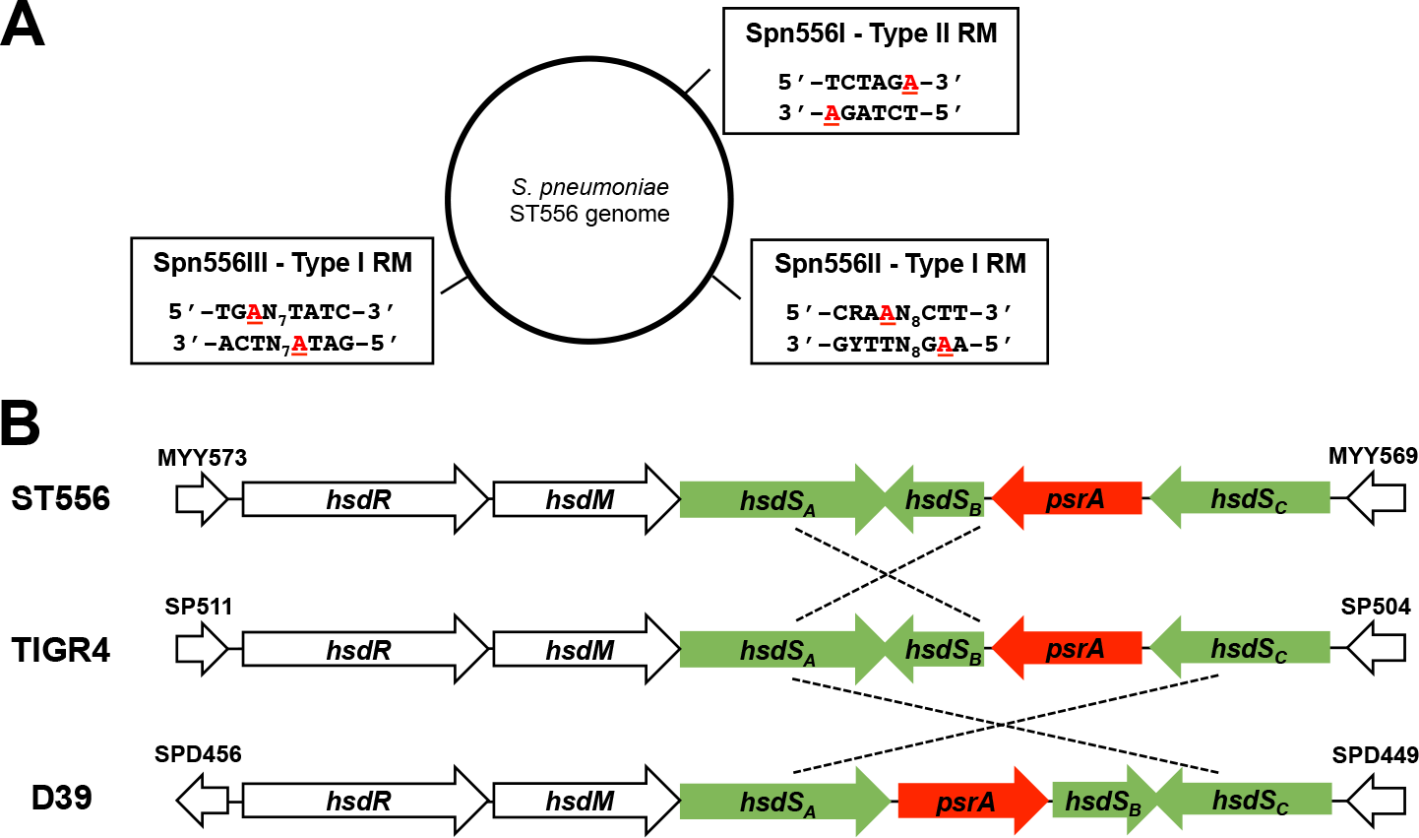
Genetic arrangement in the Spn556II locus. **A.** The three functional DNA methylation motifs recognized by the three R-M systems in strain ST556 according to our previous study [35]. The methylated bases are highlighted with red characters. **B.** The gene order and other features in the Spn556 locus of three pneumococcal strains. The orientations of the coding sequences are indicated by arrowheads. Each *hsdS* segment with identical or nearly identical sequences between the two of three strains (ST556, TIGR4 and D39) is indicated with a dashed line. Drawing is not to scale.

Our recent study identified three DNA motifs specifically methylated by three R-M systems (two type-I: Spn556II and Spn556III; a type-II: Spn556I) in the multi-drug resistant type 19F strain ST556 of *S*. *pneumoniae* (Fig 1A) [35]. We observed remarkable heterogeneity in methylation patterns of the DNA motifs recognized by the Spn556II type-I R-M system, as reflected by the median methylation proportion (MMP) values. MMP was estimated by a qDNAmod bioinformatic tool [35], which is available at https://github.com/zhixingfeng/qDNAmod/releases. Approximately 50% of the 1,026 loci of the methylation motif (5’-CRAAN_8_CTT-3’) in the sequenced copies of the ST556 genome (MMP = 0.5) remained unmethylated in a clonal population of ST556. In contrast, the 664 loci of the XbaI-like Spn556I (type-II) recognition sequence (5’-TCTAGA-3’) were almost completely methylated in all sequenced genome copies (MMP = 0.92). In this context, this finding suggests that the Spn556II recognition sequence is methylated in some of pneumococcal cells.

To uncover the molecular and genetic basis of this heterogeneity in DNA methylation, we characterized the Spn556II locus in this study. The Spn556II locus in ST556 consists of 8,076 nucleotides or six genes (Fig 1B). The first two genes code for two of the three subunits of the typical type-I R-M system: the endonuclease (HsdR, MYY572) and methyltransferase (HsdM, MYY571). Three of the downstream genes (MYY570, MYY2565, MYY2555) are homologous to the genes encoding the sequence specificity (HsdS) subunits of the bacterial type-I R-M systems. We designated the three *hsdS* genes as *hsdS*_*A*_ (MYY570), *hsdS*_*B*_ (MYY2565) and *hsdS*_*C*_ (MYY2555). Sequencing analysis revealed that *hsdS*_*A*_ (1,566 base pair, bp) and *hsdS*_*C*_ (1,281 bp) each consist of two variable regions encoding two TRDs, whereas *hsdS*_*B*_ (609 bp) encodes only one TRD. The gene (MYY2560) between *hsdS*_*B*_ and *hsdS*_*C*_ was designated as pneumococcal site-specific recombinase A (*psrA*) because of its homology with tyrosine site-specific recombinases of the DNA breaking-rejoining enzyme super-family. Although the Spn556II locus is highly conserved among the genomes of all *S*. *pneumoniae* strains available to date, there is striking heterogeneity in DNA sequence configurations. As an example, the 3’ sequence of *hsdS*_*A*_ in type-4 strain TIGR4 is placed in the position of *hsdS*_*B*_ with an inverted orientation in ST556 (Fig 1B). Similarly, the orientations and/or sequences of the four genes in the *hsdS* locus in type-2 strain D39 are differently arranged from those of TIGR4 and ST556. These peculiar sequence arrangements raised the possibility of DNA inversion in this region. To avoid potential confusions between the sequences and loci of three *hsdS* genes, we designated these genes by their genomic positions instead of specific sequences (Fig 1B). As an example, the first *hsdS* gene is always referred to as *hsdS*_*A*_ regardless its sequence nature.

**Fig 2:**
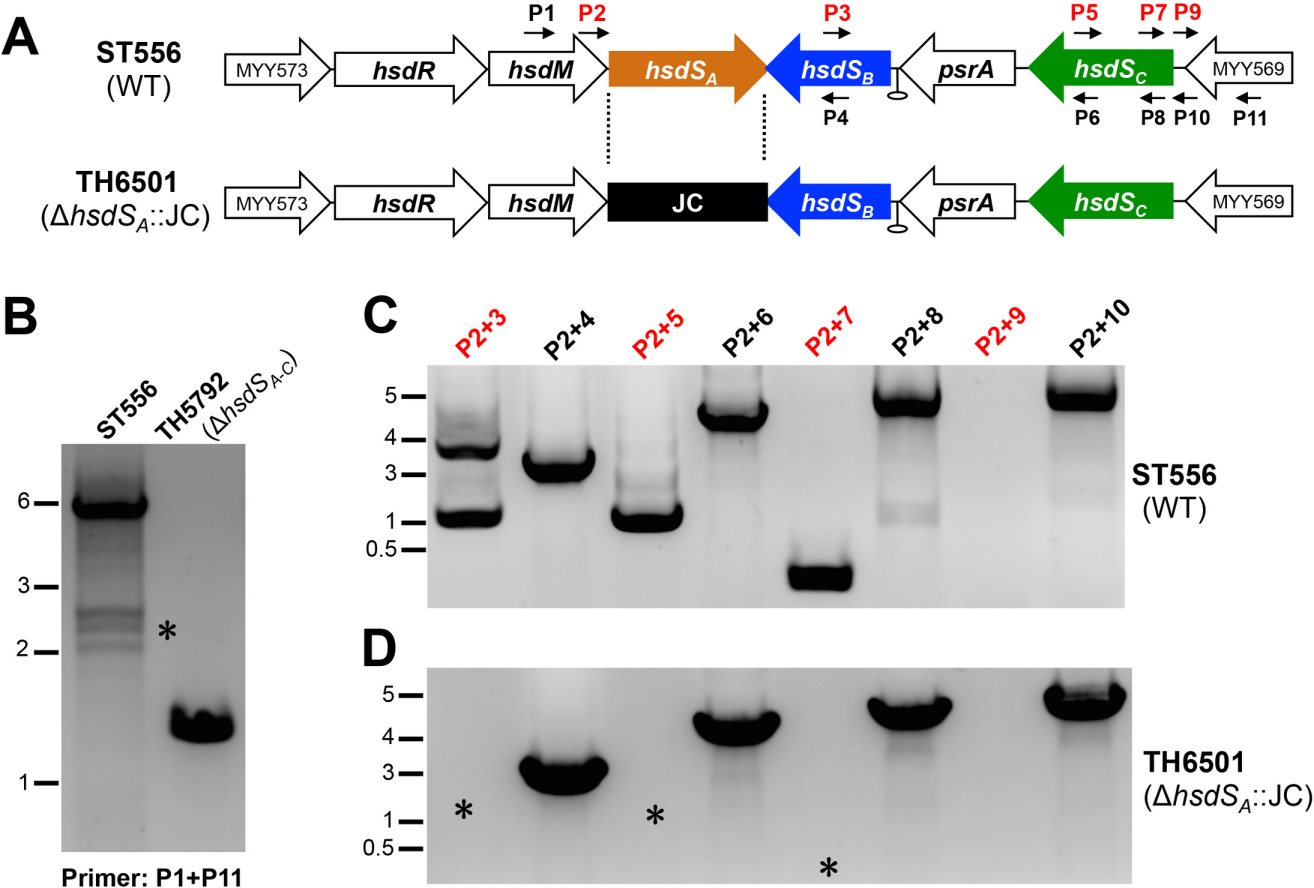
Detection of DNA rearrangements in the Spn556II locus by PCR. **A.** Positions of the primers used for PCR amplification in the Spn556II locus of ST556. The predicted rho-independent transcription terminator is indicated by a hairpin. The primers used in (**B**) and (**C**) are indicated by small arrows. The JC-replaced region in TH6501 is marked with dashed lines. **B.** Amplification of the Spn556II locus in ST556 and isogenic mutant TH5792 lacking the entire Spn556II locus with primers P1 and P11. The PCR mixtures were processed by DNA electrophoresis and stained by the Goldview dye (Yeasen, Beijing, China). The PCR products that were absent in the mutant strains are marked with asterisks (*). The sizes of the DNA markers are indicated in kilobases. **C.** Detection of DNA rearrangements in the *hsdS* regions of the Spn556II locus. PCR reactions were performed with the genomic DNA of ST556 using the same set of primer pairs indicated at the top of each lane, and marked as in (**B**). **D**. Same as in (C) except for using the genomic DNA from the ST556 derivative lacking *hsdS*_*A*_ strain (TH6501).

To test this hypothesis, we initially amplified the entire locus of ST556 and *hsdS*-null mutant TH5792 by polymerase chain reaction (PCR) with flanking primers P1 and P11. The 4.346-kilobase (kb) sequence between the start codons of *hsdS*_*A*_ and *hsdS*_*C*_ in ST556 was removed by unmarked deletion (Fig 2A and 2B). While the reaction with the wild type genomic DNA yielded a major amplicon of approximately 6 kb and multiple smaller DNA fragments, deleting the entire *hsdS* region resulted in a single amplicon of approximately 1.5 kb (Fig 2B). This result supported the notion that this locus is under DNA rearrangement. We further amplified the *hsdS* region of the Spn556II locus using a combination of the primers with the same orientations (Fig 2A). The rationale was that no PCR products should be obtained for the reactions with two forward primers unless the DNA sequence inversions had occurred in the *hsdS* region (template DNA). As an example, PCR reaction with primers P2 and P3 resulted in two products of 1.2 and 3.5 kb in size (Fig 2C); similar reactions with the same-orientation primer pairs of P2/P5 and P2/P7 also yielded PCR products. However, amplification with primers P2 and P9 (forward primer downstream of *hsdS*_*C*_) did not yield any detectable product although similar reaction with primers P2 and P10 (complementary to P9) produced a 5-kb product. This result suggested that DNA inversions occur in the *hsdS* region of the Spn556II locus.

We further tested whether these DNA rearrangements depend on *hsdS*_*A*_, the first *hsdS* gene in the locus, with a Δ*hsdS*_*A*_::JC mutant TH6501 of ST556, in which the entire *hsdS*_*A*_ coding sequence was replaced by Janus cassette (JC). Using the genomic DNA of TH6501, we were able to amplify the DNA sequences in the *hsdS* region with the combination of P2 and the downstream reverse primers (e.g., P4, P6, P8, and P10), but no amplicons were detected with the forward primers (e.g. P2/P3, P2/P5, and P2/P7)(Fig 2D). This result demonstrated that *hsdS*_*A*_ is essential for the DNA rearrangements in the *hsdS* region. The same approach also detected the products of DNA inversions with genomic DNA samples of TIGR4, but not that of isogenic Δ*hsdS*_*A*_::JC mutant TH6500 (S1A Fig). Amplifications with the same forward primer pairs of the *hsdS* sequences yielded detectable product(s) in TIGR4 (S1B Fig), but no amplicons were obtained in strain TH6500 using the same primer pairs (e.g. P2/P3’, P2/P5, and P2/P7)(S1C Fig). It should be mentioned that the amplicon profiles of TIGR4 were different from those of ST556 because of the differences in the sequence configuration or orientation in the *hsdS* region of the Spn556II locus in two strains (S1 Fig). Together, these experiments strongly suggested DNA rearrangements in the Spn556II locus in strains ST556 and TIGR4. Because all the sequenced genomes of *S*. *pneumoniae* possess the homologues of Spn556II locus, we conclude that the DNA rearrangement in the *hsdS* region is a common phenomenon in this pathogen.

### DNA rearrangements in the Spn556II locus are caused by DNA inversions and excisions in the three *hsdS* genes

**Fig 3:**
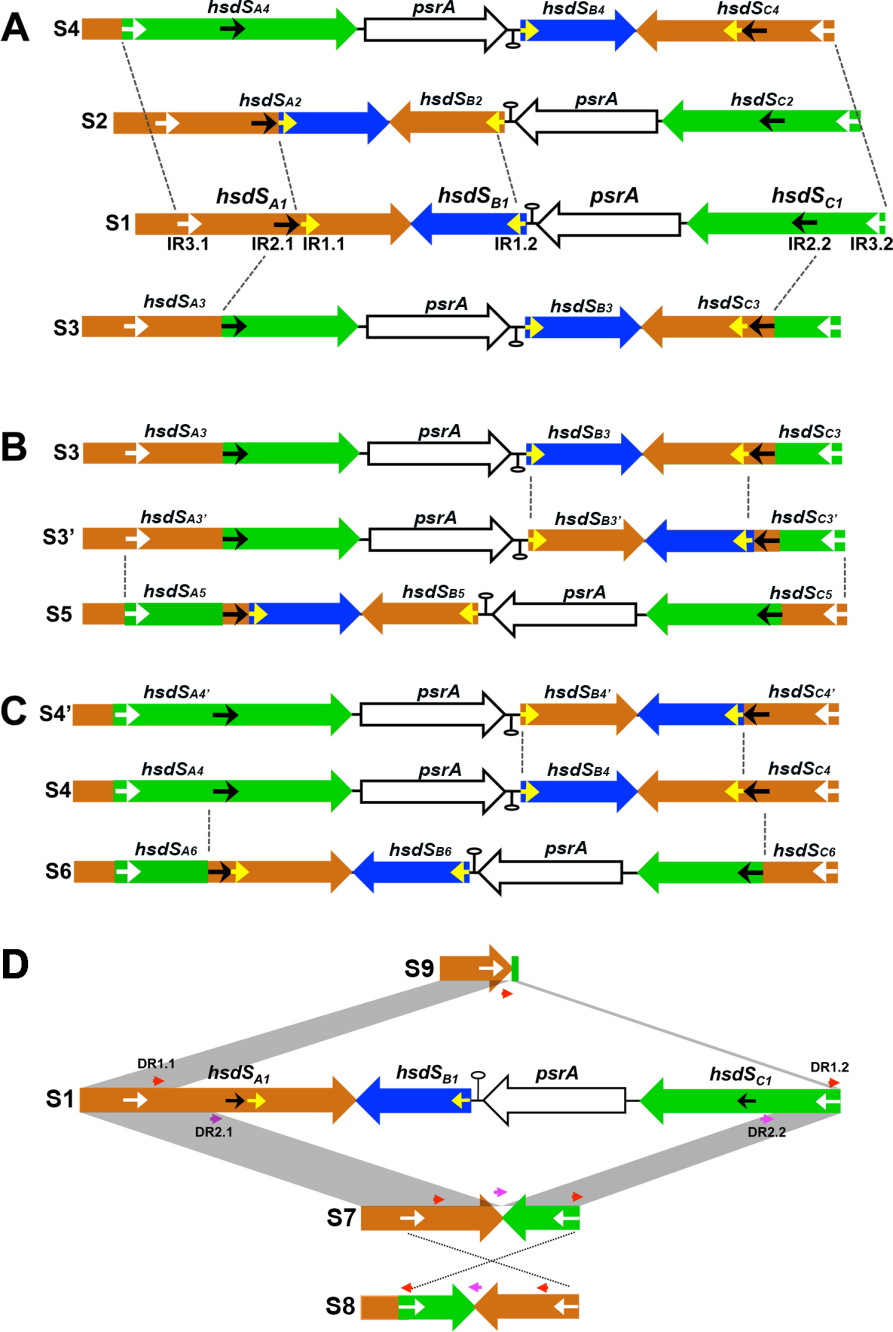
DNA configurations generated by inversions and excisions in the *hsdS* genes of the Spn556II locus. DNA configurations derived by three inversions from form S1 (**A**), S3 (**B**), or S4 (**C**). Each gene and its orientation are indicated with a large arrow. The inverted repeats (IRs) in the *hsdS* genes are represented by yellow (IR1), black (IR2), and white (IR3) arrowheads. The inversion sites are indicated by dashed lines. Each DNA configuration is assigned with an S number. DNA configurations generated by excisions between *hsdS*_*A*_ and *hsdS*_*C*_ demarcated by direct repeat sets 1 (DR1, red arrows) and (DR2, purple arrows) (**D**). Excisions mediated by the DR1 and DR2 yields *hsdS*_*A*_ variant S9 and S7. Further inversion in S7 generates S8. S7 may also generate variant S9 by further DNA excision between DR1.1 and DR1.2.

To determine the precise nature of DNA rearrangements in the *hsdS* region of the Spn556II locus, we performed DNA cloning and sequence analysis of the *hsdS* region of the Sp556II locus. The region was first amplified from the genomic DNA of ST556 with the flanking primers Pr7676 and Pr7677, cloned in the TA cloning vector, and analyzed by DNA sequencing. In combination with the single molecule real-time (SMRT) sequencing analysis, this process identified 11 different forms of sequence configuration in the *hsdS* region, which were apparently generated by DNA inversion (8 forms) and excision/inversion (3 forms) events (Fig 3). For the convenience of description, each of these sequence configurations or forms is given a unique S number, assigning the arrangement in the current genome of ST556 (accession CP003357) as S1.

As summarized in Fig 3A–3C, eight DNA arrangements were derived from DNA inversions among the three *hsdS* genes. Each inverted DNA segment is flanked by one of the three inverted repeats (IR), which are referred to as IR1 (15 bp), IR2 (298 bp), and IR3 (85 bp). While *hsdS*_*A*_ carries the forward sequences of all three IRs (IR1.1, IR2.1, and IR3.1), the inverted counterparts of these sequences are located in *hsdS*_*B*_ (IR1.2) or *hsdS*_*C*_ (IR2.2 and IR3.2). The first three forms of DNA rearrangements (S2, S3, and S4) could be generated by three independent inversions of S1 (Fig 3A). S2 matched the product of the inversion between *hsdS*_*A*_ and *hsdS*_*B*_, which is mediated by IR1.1 and IR1.2. This recombination could lead to the replacement of the 553-bp 3’ coding *hsdS*_*A*_ region with the 553-bp *hsdS*_*B*_. The resulting *hsdS*_*A*_ allele (*hsdS*_*A2*_, 1,569 bp) encodes an HsdS protein with a complete different TRD in the carboxyl region. Forms S3 and S4 were generated within the coding regions of *hsdS*_*A*_ and *hsdS*_*C*_ flanked by inverted repeats IR2 and IR3 respectively, producing two additional *hsdS*_*A*_ alleles, *hsdS*_*A3*_ (1,569 bp) and *hsdS*_*A4*_ (1,551 bp), as well as two hybrid alleles of *hsdS*_*C*_ (Fig 3A). The reverse reactions mediated by these inverted repeats would return these DNA rearrangements to the S1 form.

Four additional DNA forms (S3’, S4’, S5, and S6) were apparently generated by different DNA inversions in S2, S3, and S4. S3’ was derived from S3 by an inversion of the sequence flanked by inverted repeats IR1.1 and IR1.2 (between *hsdS*_*B*_ and *hsdS*_*C*_), which retained the same *hsdS*_*A*_ allele (*hsdS*_*A3*_) with new *hsdS*_*B*_ and *hsdS*_*C*_ alleles (Fig 3B). S5 was in turn generated from S3’ by an inversion of the sequence bound by IR3.1 (in *hsdS*_*A*_) and IR3.2 (in *hsdS*_*C*_), thereby generating a mosaic *hsdS*_*A5*_ allele (1,551 bp) that is composed of the sequences originated from all three *hsdS* genes of S1. Two additional forms (S4’ and S6) were similarly generated by two parallel inversions of S4 (Fig 3C). S4’ had the same *hsdS*_*A4*_ allele as the parental form (S4), but S6 _gained_ a new *hsdS*_*A6*_ allele (1,548 bp) consisting of the partial coding sequences from *hsdS*_*A*_ and *hsdS*_*C*_.

**Fig 4:**
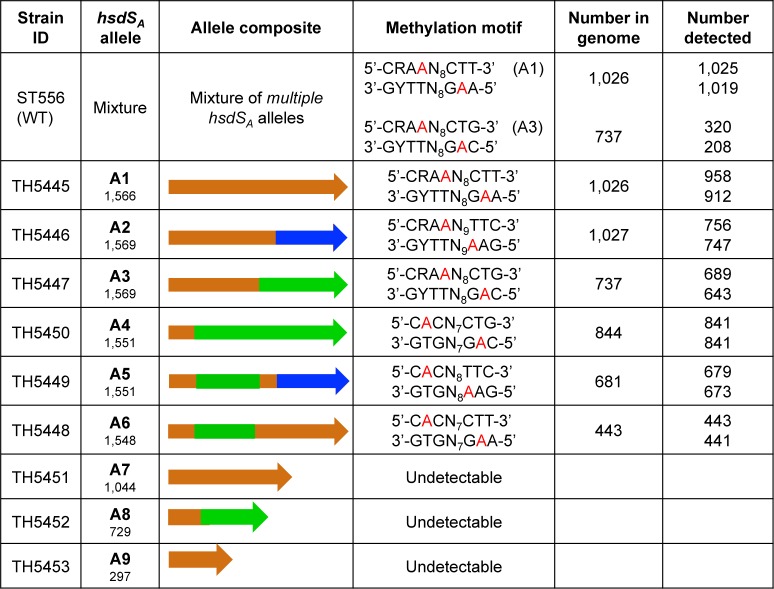
The pneumococcal genomic DNA motifs methylated by the *hsdS*_*A*_ allelic variants. The DNA methylation sequences in the genomes of strain ST556 (WT) or its derivatives (A1-A9) each possessing one of the 9 *hsdS*_*A*_ alleles were detected by SMRT sequencing. The methylation motifs recognized by Spn556I and Spn556III were also detected in all of the strains but are not shown here for the sake of space; methylated m6A bases are indicated with red characters. R = A or G, Y = T or C.

The cloning and sequencing analysis of the PCR products amplified from the *hsdS* region in the Spn556 locus with primers Pr7676 and Pr7677 also identified three truncated forms of this region (S7, S8, and S9)(Fig 3D). Form S7 suffered from a 3,143-bp truncation within the coding regions of *hsdS*_*A*_ and *hsdS*_*C*_. The truncation was flanked by two 11-bp direct repeat (DR) sequences (DR2.1 and DR2.2; 5’-TTGCTTCTATT-3’). Form S8 lacked the same sequence as S7, but the remaining segments flanked by the IR3 sequences in S7 and S8 had opposite orientations (Fig 3D). This sequence feature suggested that S8 was derived from S7 by IR3-mediated inversion. Form S9 represented the largest deletion in this locus, lacking a 4,104-bp segment of the *hsdS* region. The deleted sequence in S9 was demarcated by a pair of 12-bp imperfect DR sequence (DR1.1: 5’-ATGTTCCTTATG-3’, DR1.2: 5’- ATGTTTCTTATG-3’) and consisted of the 3’ *hsdS*_*A*_ coding sequence and the entire coding sequences of *hsdS*_*B*_, *psrA*, and *hsdS*_*C*_. As a result, forms S7, S8, and S9 encoded shorter alleles of *hsdS*_*A*_: *hsdS*_*A7*_ (1,044 bp), *hsdS*_*A8*_ (729 bp), and *hsdS*_*A9*_ (297 bp)(Fig 4). S7 and S9 appeared to be the products of two DNA excision events between the coding regions of *hsdS*_*A*_ and *hsdS*_*C*_ due to the presence of direct repeat sequences at the ends of the deleted sequences in both the forms, a typical feature of DNA excisions catalyzed by site-specific recombinases [36]. Potential deletional events responsible for the shorten *hsdS* forms are illustrated in Fig 3D, but these forms may arise from other deletional events. As an example, S8 may be generated by DNA inversion in S7 or deletion in S4. Identification of the truncated *hsdS* region revealed that the Spn556II locus undergoes DNA excisions, in addition to DNA inversions. Additional cloning and sequencing trials with the PCR products of the *hsdS* region in strain TIGR4 showed similar DNA rearrangements in the *hsdS* region of the Spn556II locus, thus confirming the previous observation by Tettelin *et al*. [26].

Together, these experiments demonstrated that the three *hsdS* genes in the Spn556II locus undergo extensive repeat sequence-mediated DNA inversions and excisions in both ST556 and TIGR4. These site-specific recombinations can generate at least 11 different forms of sequence in this locus. In the context of previous studies [26–28], and genetic conservation of this locus among strains of *S*. *pneumoniae*, these results strongly suggest that similar DNA inversions and excisions universally occur across this species.

### The *hsdS* allelic variations lead to dramatic changes in *S*. *pneumoniae* methylome

Since *hsdS* determines the sequence specificity of the type-I R-M systems, site-specific recombinations in the Spn556II locus raised the possibility of variable genome methylation profiles (methylomes) among the pneumococci carrying various *hsdS* alleles. Our SMRT sequencing only identified a methylation motif for the *hsdS*_*A*_ gene, but not *hsdS*_*B*_ or *hsdS*_*C*_ in ST556 [35](Fig 1A). This result suggested that only *hsdS*_*A*_ produces a functional S subunit of the DNA methyltransferase. Thus, our further investigation focused on characterizing *hsdS*_*A*_ allelic variation.

To determine unequivocally the impact of the *hsdS*_*A*_ recombinations on pneumococcal methylome, we engineered a series of pneumococcal derivatives each with a unique locked *hsdS*_*A*_ allele by replacing the entire 4,346-bp *hsdS* region of ST556 with one of the 9 *hsdS*_*A*_ alleles (Fig 4). Because two pairs of the *hsdS* arrangements (S3/S3’ and S4/S4’) share the identical *hsdS*_*A*_ alleles (*hsdS*_*A3*_ and *hsdS*_*A4*_), we only analyzed 9 unique *hsdS*_*A*_ alleles out of the 11 sequence configurations. As confirmed by DNA sequencing analysis, the *hsdS* DNA rearrangements were undetectable in the locked strains.

SMRT sequencing analysis revealed that ST556 and its *hsdS*_*A*_ locked strains shared the sequence motifs methylated by Spn556I (type-II R-M) and Spn556III (type-I R-M)(accession SRX1757519). In addition, the pneumococci carrying each of the locked *hsdS*_*A*_ alleles 1–6 methylated the chromosome with a unique methylation motif in the genome of ST556. This result demonstrated that these *hsdS*_*A*_ alleles are functional in dictating DNA methylation sequences. In contrast, no specific methylation motifs were detected for *hsdS*_*A*_ alleles in S7, S8 and S9, suggesting these *hsdS*_*A*_ alleles are not active due to truncations in their coding sequences. The methylation motifs for *hsdS*_*A*_ alleles 1–6 reflected the modular feature of the *hsdS*_*A*_ allelic variants. All of the HsdS_A_ alleles sharing the same *hsdS* sequence segments (or TRDs) recognized the identical DNA sequence motifs. For examples, the HsdS proteins encoded by alleles A1, A2, and A3 shared the first TRDs and the first halves of their methylation motifs (5’-CRAA-3’); the same correspondence is true between the second TRDs of alleles A3 and A4, and the second halves of their methylation motifs (5’-CTG-3’)(Figs 3A and 4). Because each of the *hsdS*_*A*_ allelic variants recognizes a different methylation motif, the site-specific recombinations in the Spn556II locus represent a novel mechanism of epigenetic diversification in this pathogen.

Since only the methylation motifs specified by *hsdS*_*A1*_ and *hsdS*_*A3*_ were detected by SMRT sequencing in ST556 (Fig 4), we assessed whether these *hsdS*_*A*_ alleles were more abundantly represented than the other *hsdS*_*A*_ alleles in the clonal populations of ST556 by comparing the number of the sequencing reads for each of the nine *hsdS*_*A*_ alleles in the SMRT sequencing data of ST556. This analysis revealed that the pneumococci carried *hsdS*_*A1*_ (62.8%), *hsdS*_*A2*_ (20.4%), or *hsdS*_*A3*_ (12%); the pneumococci carrying the other six *hsdS*_*A*_ alleles contributed <5% of the sequencing reads. These results suggested that the pneumococci carrying the *hsdS*_*A1*_ allele represented the most dominant subpopulation in the clonally derived culture that was used to prepare the genomic DNA for SMRT sequencing. The allelic dominance of *hsdS*_*A1*_ was corroborated by the overwhelming proportion of the opaque colonies in cultured clonal ST556 populations (S1 Table), suggesting that different forms of the *hsdS* arrangements are generated at uneven rates under the laboratory conditions.

### Tyrosine recombinase PsrA catalyzes one of the three ON/OFF DNA inversions associated with the *hsdS*_*A1*_ allele

**Fig 5:**
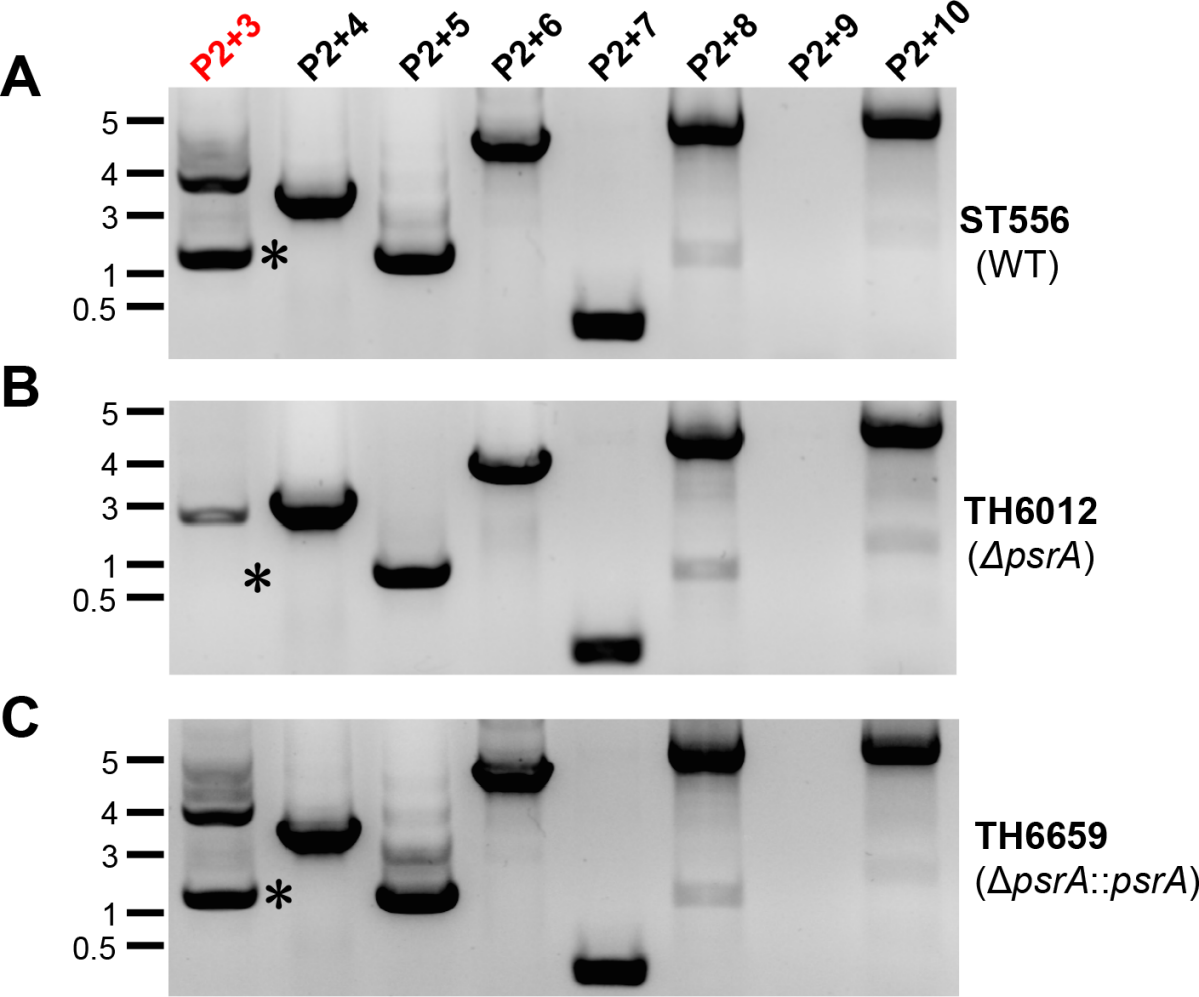
Requirement of *psrA* for the DNA inversion between the inverted repeats (IR1.1 and IR1.2). Amplification of the Spn556II *hsdS* region in ST556 (upper panel)(**A**), isogenic mutant lacking *psrA* (TH6012, middle panel)(**B**), or complemented TH6012 with the wild type *psrA* gene (TH6659, lower panel)(**C**) with primer pairs indicated at the top of each lane as in Fig 2C. The major band absent in TH6012 is marked with an asterisk (*).

Because the *hsdS* inversions in the Spn556II locus led to remarkable switches in pneumococcal methylome (Fig 4), we attempted to identify the DNA recombinase(s) that are involved in these recombination events. We initially tested the impact of the putative recombinase encoded by *psrA* in this locus. A *psrA* unmarked deletion mutant TH6012 was constructed in ST556. Direct comparison of the *hsdS* recombination profiles between ST556 and TH6012 by PCR amplification showed that the DNA inversion mediated by inverted repeat IR1 was detectable in the wild type (Fig 5B; P2+3) but not in the Δ*psrA* mutant as highlighted by an asterisk in Fig 5B. In contrast, the DNA inversions mediated by the IR2 (Fig 5B; P2+5) and IR3 (Fig 5B; P2+7) remained intact in the Δ*psrA* mutant. This result suggested that *psrA* is involved in the IR1-mediated inversion between forms S1 and S2 (Fig 3A). To define the role of PsrA in the *hsdS* rearrangement, we complemented the *psrA* deletion in strain TH6012 by knocking in the wild type *psrA* in the original chromosomal position of *psrA*. The resulting strain TH6659 re-gained the capability of the IR1-mediated inversion between *hsdS*_*A*_ and *hsdS*_*B*_ as the parent strain ST556 (Fig 5C). This result demonstrated that PsrA catalyzes the DNA inversion of the sequence bounded by IR1.1 and IR1.2, and thus controls the reversible switch between alleles A1 and A2 of *hsdS*_*A*_ in the Spn556II locus (Figs 3A and 4). Since *psrA* is also present in TIGR4 and many other *S*. *pneumoniae* strains with the complete genome sequences, we tested its impact on the *hsdS* rearrangements in TIGR4. As shown in S2 Fig, the Δ*psrA* mutant of TIGR4 (TH6555) lacked the inversion of sequence between *hsdS*_*A*_ and *hsdS*_*B*_, but this mutation did not show detectable impact on other recombinations.

We also tested the impact of 13 additional genes that encode known or putative recombinases, including *xerS* (MYY1187), *recA* (MYY1848), MYY32, MYY49, MYY258, MYY375, MYY1100, MYY1101, MYY1188, MYY1181, MYY1348, MYY1379, and MYY1771. XerS is a tyrosine recombinase catalyzing chromosome dimer resolution [37]; RecA a recombinase for homologous recombination and DNA repair [38]. Deleting each of these genes in ST556 did not show detectable impact on any of the inversions in the Spn556II locus. These data demonstrated that multiple recombinases are involved in the DNA rearrangements of the Spn556II locus, and PsrA is only involved in the IR1-mediated inversion between *hsdS*_*A*_ alleles A1 and A2 in the Spn556II locus of strains ST556 and TIGR4. The DNA recombinase(s) catalyzing additional inversions and excisions remains to be identified.

### The *hsdS*_*A*_ allelic switches alter *S*. *pneumoniae* restriction activity

We further tested whether DNA rearrangements in the Spn556II locus lead to diversification in pneumococcal restriction activity. Unmethylated pIB166 shuttle plasmids (each carrying multiple copies of a unique methylation motif) were individually transformed in ST556 or its derivatives each with a locked *hsdS*_*A*_ allele. Transformation of ST606 with the plasmid carrying the recognition sequences of HsdS_A1_, HsdS_A2_, or HsdS_A3_ yielded less than 50% of the transformants that were generated with the motif-less plasmid. This result indicated that *hsdS*_*A*_ rearrangements resulted in functional diversification in restriction spectrum of an originally clonal pneumococcal population (S3A Fig) The same procedure revealed even lower levels of transformation when the plasmids harboring the recognition motifs (A1, A2, and A3) were used to transform the strains expressing the corresponding *hsdS*_*A*_ alleles (TH5445, TH5446, and TH5447; S3B–S3D Fig). This result demonstrated that allelic variation in *hsdS*_*A*_ generates diversity in restriction activity of clonal pneumococcal populations.

### The *hsdS*_*A*_ allelic switches correlate with pneumococcal phase variation in colony opacity

**Fig 6:**
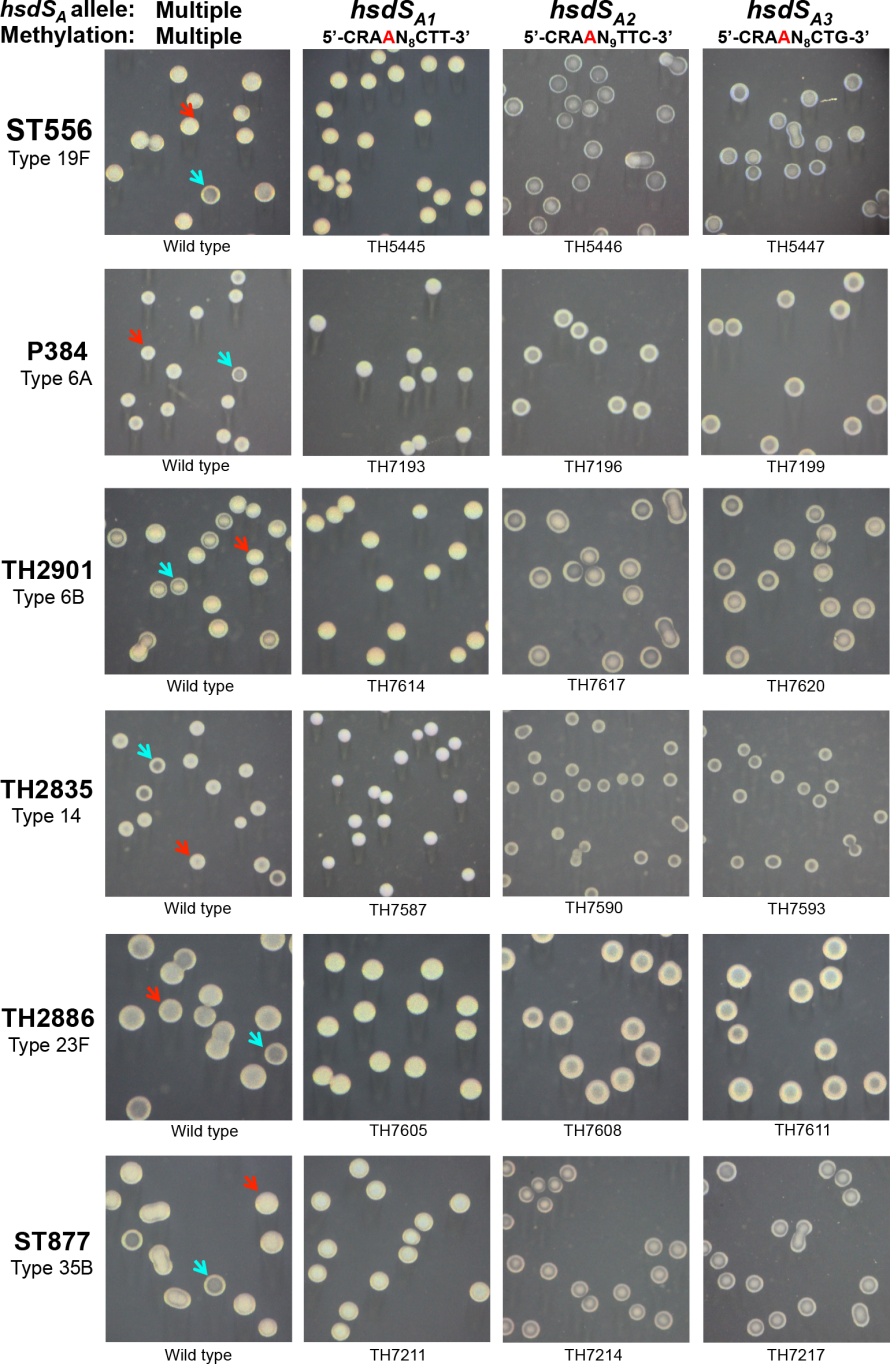
Colony morphology of six *S*. *pneumoniae* strains and their derivatives each carrying an invariable *hsdS*_*A*_ allele. Pneumococcal strains ST556 (19F), P384 (6A), TH2901 (6B), TH2835 (14), TH2886 (23F), and ST877 (35B) were grown on TSA plates supplemented with catalase; the colonies photographed under a dissection microscope as described in reference [33]. The Spn556II *hsdS*_*A*_ genotype and corresponding profile of chromosomal methylation in each strain are marked at the top of each column. Strain designation is indicated at the bottom of each photograph. The representative colonies with opaque and transparent appearance in the wild types are highlighted with blue and red arrowheads, respectively.

To determine potential phenotypic impact of the site-specific *hsdS* recombinations on other aspects of pneumococcal biology, we systematically compared the *hsdS*_*A*_ allelic variants, in terms of the well-characterized properties that are important for pneumococcal pathogenesis. This screening revealed a striking difference between the pneumococci harboring *hsdS*_*A1*_ and other *hsdS*_*A*_ alleles in colony opacity on the TSA plates (without blood). Phase variation between opaque and transparent colony forms has been well documented to impact nasopharyngeal colonization and virulence in *S*. *pneumoniae* [29, 30, 33], but the molecular mechanism behind the generation of phase variation has remained mysterious. Microscopic examination revealed that the parent strains (ST556 and ST606) with the intact Spn556II locus are capable of reversible switch between the opaque and transparent colony phenotypes (Fig 6, first column; S4 Fig; S1 Table). However, the isogenic pneumococci carrying only the *hsdS*_*A1*_ allele (TH5445) produced uniformly opaque colonies (Fig 6, second column; S5 Fig; S1 Table). In sharp contrast, the other eight *hsdS*_*A*_ alleles always yielded transparent colonies with occasional formation of opaque colonies (Fig 6, S5 Fig; S1 Table). All of the opaque colonies formed by the *hsdS*_*A2*_ -*hsdS*_*A9*_ variants tested thus far had the same spontaneous mutations in the pyruvate oxidase *spxB* gene as described by Ramos-Montanez *et al*. [39], which lead to opaque colony phenotype [39]. This experiment revealed a strong correlation between the *hsdS*_*A1*_ allele and opaque colony phenotype.

**Fig 7:**
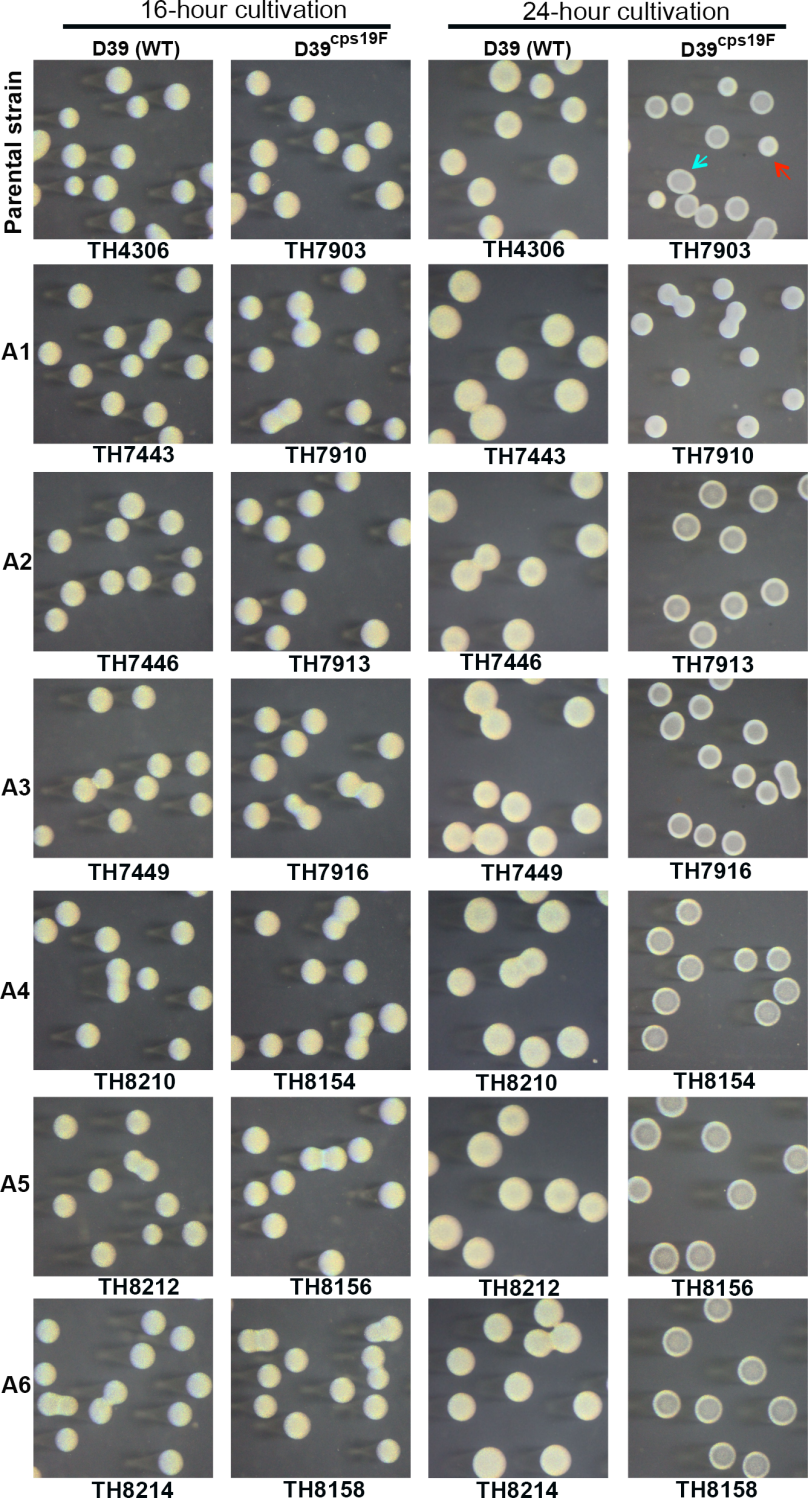
Colony morphology of strain D39 and its derivatives each carrying one of the 6 invariable *hsdS*_*A*_ alleles. The *hsdS*_*A*_ allele-locked derivatives of strain D39 (WT, type 2) and isogenic capsule switch variant producing a type-19F capsule (D39^cps19F^) were grown for 16 or 24 hours and processed as described in Fig 6. The name of each strain is listed at the bottom of each photograph; the *hsdS*_*A*_ allele genotype marked at the bottom and left side of each row. The representative colonies with opaque and transparent appearance in the parental strains are indicated with blue and red arrowheads, respectively.

To rule out potential background effect, we constructed similar *hsdS*_*A*_ allele-locked derivatives of strains P384 (type 6A), TH2901 (type 6B), TH2835 (type 14), TH2886 (type 23F), ST877 (type 35B), D39 (type 2), and TIGR4 (type 4). Strain P384 (type 6A) was previously used to study pneumococcal phase variation in colony morphology [40, 41]. While all of the wild type strains, except for D39 and TIGR4, produced both opaque and transparent colonies, the isogenic derivatives carrying the *hsdS*_*A1*_ allele formed uniformly opaque colonies in the five strain backgrounds (Fig 6). Similar to the ST556 derivatives, all of the *hsdS*_*A2*_- or *hsdS*_*A3*_-locked derivatives of P384, TH2901, TH2835, TH2886, and ST877 displayed the transparent colony phenotype. In sharp contrast, the wild type strains of D39 and TIGR4 and all of their isogenic *hsdS*_*A*_ allele-locked derivatives showed uniformly opaque colonies at the 16^th^ and 24^th^ hour of cultivation (Fig 7, columns 1 and 3; S7 Fig). With the exception of strains D39 and TIGR4, this result confirmed the association between the *hsdS*_*A1*_ allele and opaque colony phenotype.

To define the impact of the *hsdS*_*A*_ allelic variation on the colony phenotypes in D39 and TIGR4, we initially swapped the entire capsule gene clusters between D39 (with a type-2 capsule) and ST556 (with a type-19F capsule) by counter selection since the capsule types can alter the appearance of pneumococcal colonies [29]. The capsule-swapping derivatives of D39 and ST556 (respectively referred to as D39^cps19F^ and ST556^cps2^ hereafter) were used as the parental strains to generate single *hsdS*_*A*_ allele-locked mutants. Similar to the wild type D39, D39^cps19F^ and its derivatives (each with a unique *hsdS*_*A*_ allele) produced uniformly opaque colonies at the 16^th^ hour of cultivation (Fig 7, columns 1 and 2). However, extended cultivation led to the formation of mixed opaque and transparent colonies (Fig 7, D39^cps19F^). At the 24^th^ hour of cultivation, the D39^cps19F^ derivative carrying the locked allele A1 of *hsdS*_*A*_ formed opaque colonies, whereas those with alleles A2-6 produced transparent colonies (Fig 7, rows 2–7 of column 4). This result indicated that the *hsdS*_*A*_ allele-associated phase variation operates in strain D39 as the other *S*. *pneumoniae* strains represented in Fig 6, but the type-2 capsule can obscure the morphologic differences between the opaque and transparent colonies in D39. In the reciprocal direction of the capsule swapping, ST556^cps2^ and its *hsdS*_*A*_-locked derivatives behaved like the counterparts of the wild type ST556 in colony phenotypes, forming a mixture of opaque and transparent (ST556^cps2^), opaque (allele A1), or transparent (alleles A2-6) colonies at the 16^th^ hour of cultivation, despite the fact that the opaque and transparent colonies of ST556^cps2^ were less distinguishable than those of the wild type ST556 (S6 Fig). This result demonstrated that both the capsule type and strain background exert substantial impact on the development of morphologically differentiable opaque and transparent colonies. Similar procedures revealed that TIGR4 also formed opaque and transparent colonies in the same *hsdS*_*A*_ allele-dependent manner as D39 once the type-4 capsule is removed in this strain (S7 Fig). Together, these results have demonstrated a strict association between the allelic variants of the *hsdS*_*A*_ gene and colony phenotypes (A1—opaque; A2-6—transparent) in all eight pneumococcal strains tested thus far. Because each strain tends to have a different designation for this locus (e.g. Spn556II and SpnD39III) according to the nomenclature rules of the R-M systems [42], we thus propose a functional name for this locus: colony opacity determinant (*cod*).

It should be noted that a recent study also reported that *hsdS*_*A*_ (or SpnD39III) allelic variation influences colony opacity in strain D39 [28], but its result is partially discrepant from what is described above in two aspects. First, the *hsdS*_*A*_ allele SpnIIIA (A3 in this study) and SpnIIIE (A1 in this study) were previously described to be associated with the opaque colonies [28], but only the pneumococci carrying locked allele A1 produced opaque colonies in at least eight strain backgrounds test thus far (including D39^cps19F^) in this work; the other alleles unanimously yielded transparent colonies. Second, Manso *et al*. observed that certain *hsdS*_*A*_ alleles (e.g. A2—SpnIIIB, A4—SpnIIID, A5—SpnIIIC, and A6—SpnIIIF) yielded mixtures of opaque and transparent colonies in the D39 background [28]. However, similarly constructed pneumococcal derivatives of D39^cps19F^ (Fig 7) and six other strains (Fig 6) formed uniformly transparent colonies. Potential cause for these discrepancies is elaborated in Discussion.

### The capsule is dispensable for the phase variation associated with the *hsdS*_*A*_ allelic switches

**Fig 8:**
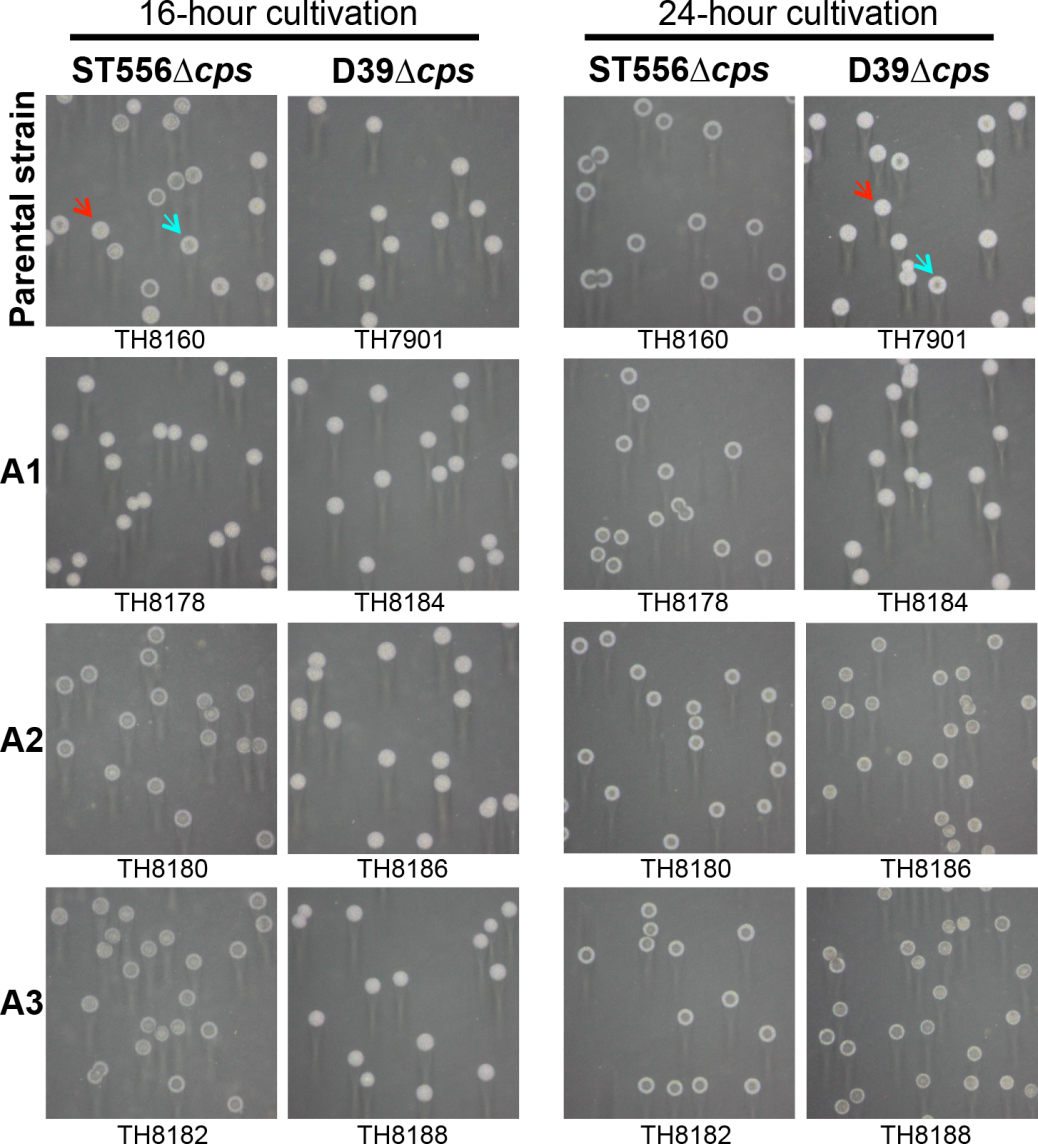
Epigenetic-driven phase variation in unencapsulated pneumococci. The unencapsulated mutants of ST556 (parental strain TH8160) and D39 (parental strain TH7901) were used to generate single *hsdS*_*A*_ allele-locked strains by counter selection as described in Fig 6. Colonies of each strain were prepared and photographed as in Fig 6. The strains in each row shared the same *hsdS*_*A*_ allele (A1, A2, or A3). The strains in each column were constructed from the same unencapsulated parental strain (top of each column). The name of each strain is listed at the bottom of each photograph.

The capsule swapping experiment revealed significant impact of capsule type on the extent of the colony phenotypes (Fig 7 and S6 Fig), but it was unclear if the capsule is required for the phase variation driven by the *hsdS*_*A*_ allelic variation. We thus determined the phase variation of unencapsulated pneumococci in the strain backgrounds of ST556 and D39. Similar to the encapsulated parental strain (Fig 6 and S5 Fig), unencapsulated ST556 (TH8160) produced a mixture of opaque and transparent colonies at the 16^th^ hour of cultivation; its derivatives showed an opaque (strain TH8192 carrying allele A1 of *hsdS*_*A*_) or transparent colony phenotype (strains TH8194 and TH8196 with alleles A2 and A3, respectively) (Fig 8). At the 24^th^ hour of cultivation, the unencapsulated derivatives of D39 displayed the same pattern of the *hsdS*_*A*_ allele-dependent colony phenotypes as the counterparts of ST556 (Fig 8, column 4). In agreement with the encapsulated counterparts (S6 Fig), the unencapsulated ST556 pneumococci formed uniformly transparent colonies with a typical “crater” structure in the center of the colonies at the 24^th^ hour of cultivation (Fig 8, column 3), whereas the colonies of the D39 derivatives were uniformly opaque at the 16^th^ hour of cultivation (column 2). Lastly, the unencapsulated TIGR4 derivatives also displayed the same relationship between the *hsdS*_*A*_ alleles and colony phenotypes at the 24^th^ hour of cultivation although the wild type TIGR4 behaved as D39 with uniformly opaque colonies at both the 16^th^ and 24^th^ hours of growth (S7 Fig). This experiment demonstrated that the pneumococcal capsule is not required for the phase variation in colony opacity.

### The phase variation depends on the DNA methyltransferase activity of HsdM

**Fig 9:**
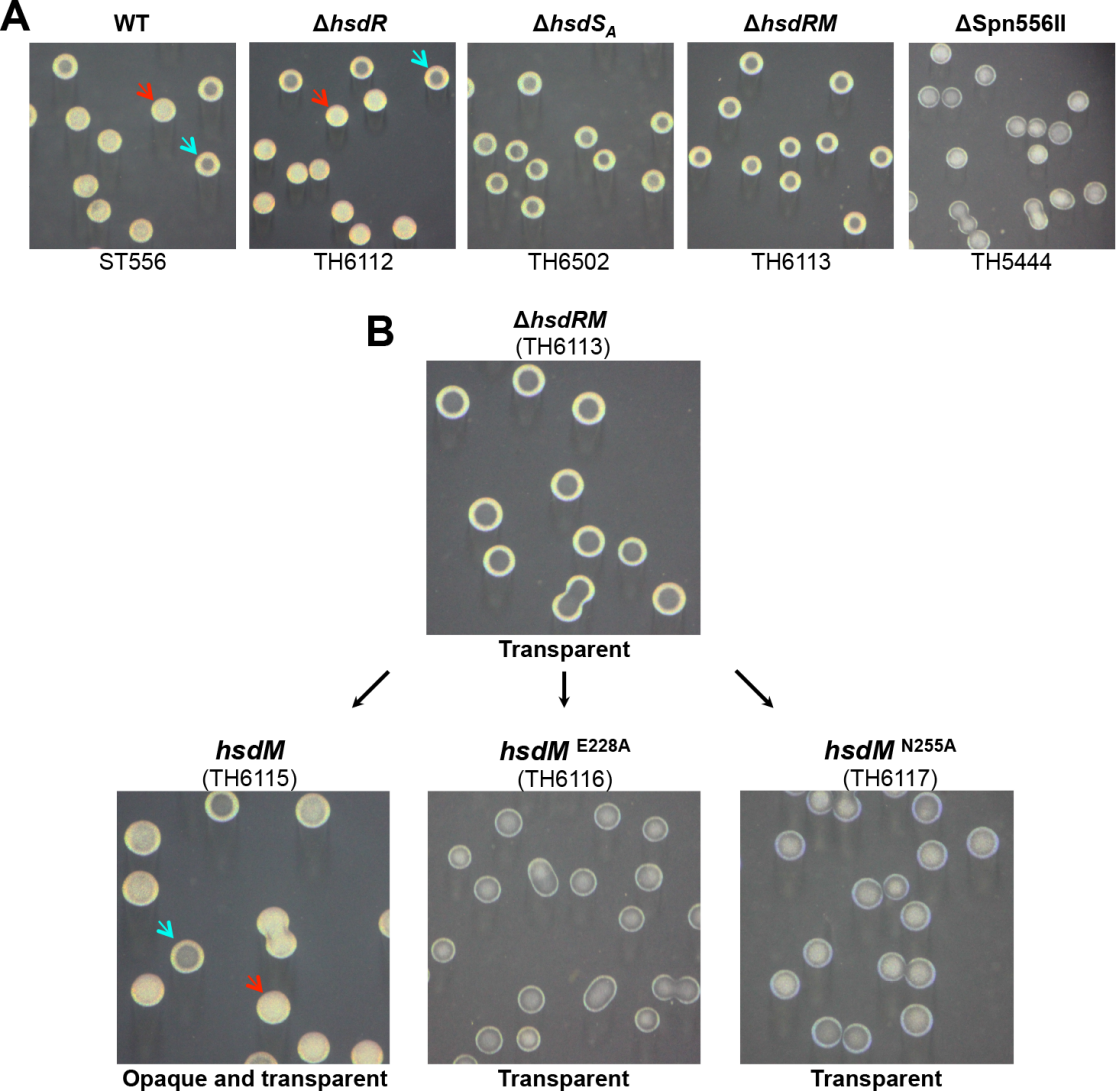
Essential roles of the DNA methyltransferase activity in defining pneumococcal colony opacity. **A.** Necessity and sufficiency of *hsdM* and *hsdS*_*A*_ in defining pneumococcal colony opacity. Isogenic mutants each with an unmarked deletion in the coding region of *hsdR*, *hsdS*_*A*_, *hsdRM or* Spn556II were constructed in the Spn556II locus of ST556. Colonies of each strain were prepared, photographed, and marked as in Fig 6. **B.** Requirement of the DNA methyltransferase catalytic activity in defining pneumococcal colony opacity. Strain TH6113 lacking the entire coding region of *hsdR* (MYY572) and *hsdM* (MYY571) (producing transparent colonies) was complemented with either the wild type *hsdM* gene (MYY571) or its catalytically inactive mutant with an E228A or N255A point mutation. Colonies are presented as in (**A**).

To define the mechanism of the *hsdS*_*A*_-associated phase variation, we determined whether the R and M subunits of the Spn556II type-I R-M system are involved in the colony opacity phase variation by generating unmarked deletions in *hsdR*, *hsdM*, and *hsdS*_*A*_ of ST556. While the Δ*hsdR* strain (TH6112) did not display detectable difference from the parent strain in colony morphology, the mutants lacking *hsdS*_*A*_ (TH6502), *hsdRM* (TH6113), or Spn556II (TH5444) formed uniformly transparent colonies (Fig 9A). This result showed that both *hsdM* and *hsdS*_*A*_ but not *hsdR* are essential for phase variation in colony opacity, indicating that the DNA methyltransferase activity but not restriction activity of the Spn556II R-M system is required for this phenotype.

To establish a causal relationship between the catalytic activity of HsdM in DNA methylation and the phase variation, we complemented the Δ*hsdRM* mutant (TH6113) with wild type or mutant forms of *hsdM* (MYY571). The wild type *hsdM* yielded both opaque and transparent colonies (Fig 9B), indicating successful restoration of the phase variation. In contrast, two mutant *hsdM* alleles (E228A and N255A) failed to complement the colony phenotype of the Δ*hsdRM* mutant. E228 and N255 represent two of the amino acid residues that are essential for the catalytic activities of the N6-adenine DNA methyltransferases [43]. Accordingly, DNA methyltransferase activity was detected only with the wild type HsdM, but not its E228A and N255A mutants by DNA methylation protection (S8 Fig). The *hsdM* complementation experiment showed that pneumococcal phase variation in colony opacity depends on the methyltransferase activity of the *cod* locus. The essentiality of the methyltransferase activity for pneumococcal phase variation was also confirmed by a similar complementation experiment in the *hsd*_*A1*_∆*hsdRM* mutant of ST556 (TH7928). Only the wild type *hsdM*, but not its point mutants, restored the opaque colony phenotype of allele A1 (S9 Fig). Taken together, these results unequivocally demonstrate that the *hsdS* gene inversion-driven epigenetic diversity leads to striking switch between the colony phenotypes of *S*. *pneumoniae*.

### Reversible ON/OFF switch of the *hsdS*_*A1*_ allele is sufficient for phase variation in pneumococcal colony opacity

**Fig 10:**
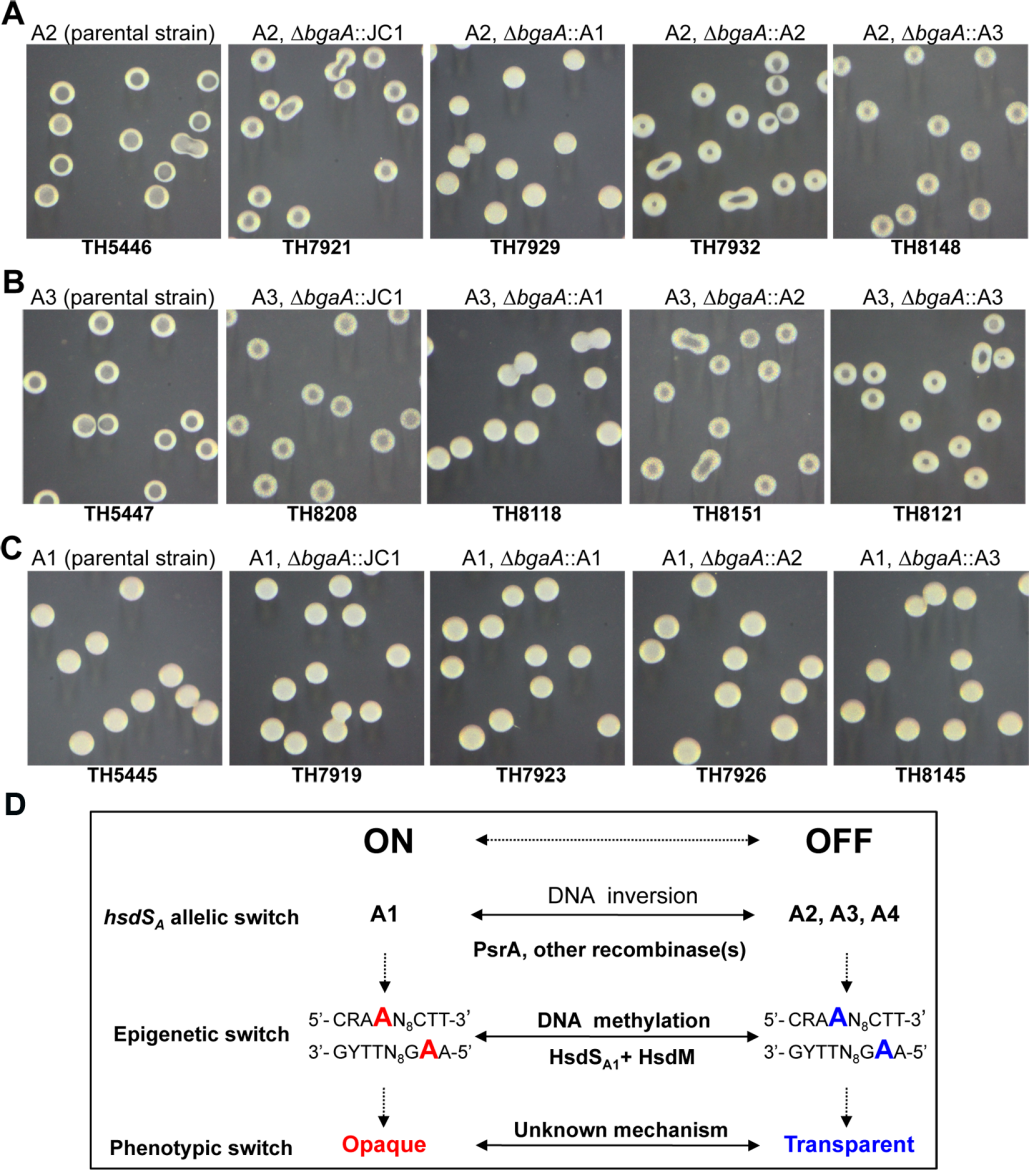
Allele A1 of *hsdS*_*A*_ dictates the opaque colony phenotype of *S*. *pneumoniae*. Chromosomal co-expression of *hsdS*_*A*_ alleles A1-A3 in the *bgaA* locus of the ST556 derivatives carrying the locked *hsdS*_*A*_ allele A2 (**A**), A3 (**B**) or A1 (**C**). A modified Janus cassette (JC1) was used to replace partially the coding sequence of *bgaA* in each parent strain. JC1 in the resulting strains were subsequently replaced by the fusion PCR product of the A1, A2, or A3 allele of *hsdS*_*A*_ by counter selection, which consisted of the *hsdRMS* promoter and full coding sequence of each allele. Colonies of each strain were prepared, photographed, and marked as in Fig 6. The genotype and name of each strain are marked at the top and bottom of each photograph. All of the ST556 derivatives carrying allele A1 produced uniformly opaque colonies. In (**D**), the relationship of the *hsdS*_*A*_ allelic variations by DNA inversions and the resulting epigenetic and phenotypic switch is diagrammatically illustrated; the methylated and unmethylated adenine nucleotides in the DNA motif by the Hsd_A1_-associated methyltransferase is highlighted with red and blue characters, respectively. R = A or G, Y = T or C.

We further determined whether switching the *hsdS*_*A*_ alleles in the same S. *pneumoniae* strain background is able to reverse the opaque and transparent colony phenotypes, a requirement for phase variation [44], by co-expressing two different *hsdS*_*A*_ alleles in the same strains. The alleles A1, A2, and A3 were individually inserted in the *bgaA* locus of the ST556 derivatives each carrying a locked A1, A2, or A3 allele in the *cod* locus. *bgaA*, encoding a dispensable β-galactosidase [45], has been previously used to express exogenous genes in *S*. *pneumoniae* [46], and partial replacement of *bgaA* with JC1 (modified Janus cassette, see MATERIALS AND METHODS) did not have apparent effect on the colony phenotypes of the resulting strains (TH7919, TH7921, and TH8208) (Fig 10). Genetic addition of the opaque phenotype-defining allele A1 to strain TH5446 (with a locked A2 allele) led to the conversion of colony phenotype from transparent (in the parental strain) to opaque (in the resulting strain TH7929) (Fig 10A). Similar procedure with allele A1 also switched the colony morphology of the A3-locked strain (TH5447) from transparent to opaque (in strain TH8118) (Fig 10B). However, genetically overlaying allele A2 or A3 to the allele-A1 strain TH5445 did not have any detectable impact on its colony phenotype (in the resulting strains TH7926 and TH8145)(Fig 10C).

The *hsdS*_*A*_-allele overlay experiment demonstrated that the *hsdS*_*A*_ allelic variant A1 is functionally dominant over alleles A2 and A3 in defining the opaque colony phenotype of *S*. *pneumoniae*. Because the pneumococci carrying all non-A1 alleles (e.g. A2-A6) shared the same colony phenotype with all of the existing loss-of-function mutants in this locus (e.g. ΔSpn556II—TH5444, Δ*hsdRM*—TH6113, Δ*hsdS*_*A*_—TH6502; Fig 9A), formation of the transparent colonies represents a default phenotype for the OFF phase of allele A1. In other words, the epigenetic statuses defined by other non-A1 alleles of *hsdS*_*A*_ are not required for the phase variation. These data have delineated a mechanistic pathway from DNA inversions among three *hsdS* genes, alternating methylation of pneumococcal genome, to phase variation in colony opacity (Fig 10D). In this pathway, site-specific recombinase-catalyzed DNA inversions drive reversible ON/OFF allelic switch between the A1 and other alleles (A2, A3, and A4) of *hsdS*_*A*_ in the *cod* locus, leading to ON/OFF switch in the A1-associated DNA methyltransferase activity and thereby epigenetic (or methylation) switch of pneumococcal genome. Finally, the epigenetic switch driven by DNA inversions results in bacterial phase variation in colony opacity. In agreement with this logic, both the opaque and transparent variants of the *psrA*-deficient ST556 (TH6012) showed substantially lower levels of phase switch although the *psrA* mutant was still capable of phase variation with extensive passaging (S1 Table). Based on the SMRT sequencing data of limited strains (e.g. ST556, TIGR4, and D39), the pneumococcal genome contains more than 1,000 copies of the methylation motif (1,026 copies in ST556) recognized by HsdS_A1_. The specific locus (or loci) of the motif associated with the opaque colony phenotype remains to be determined.

### The epigenetic-driven phase variation generates pneumococcal subpopulations with variable capacity in adhesion to host epithelial cells

**Fig 11:**
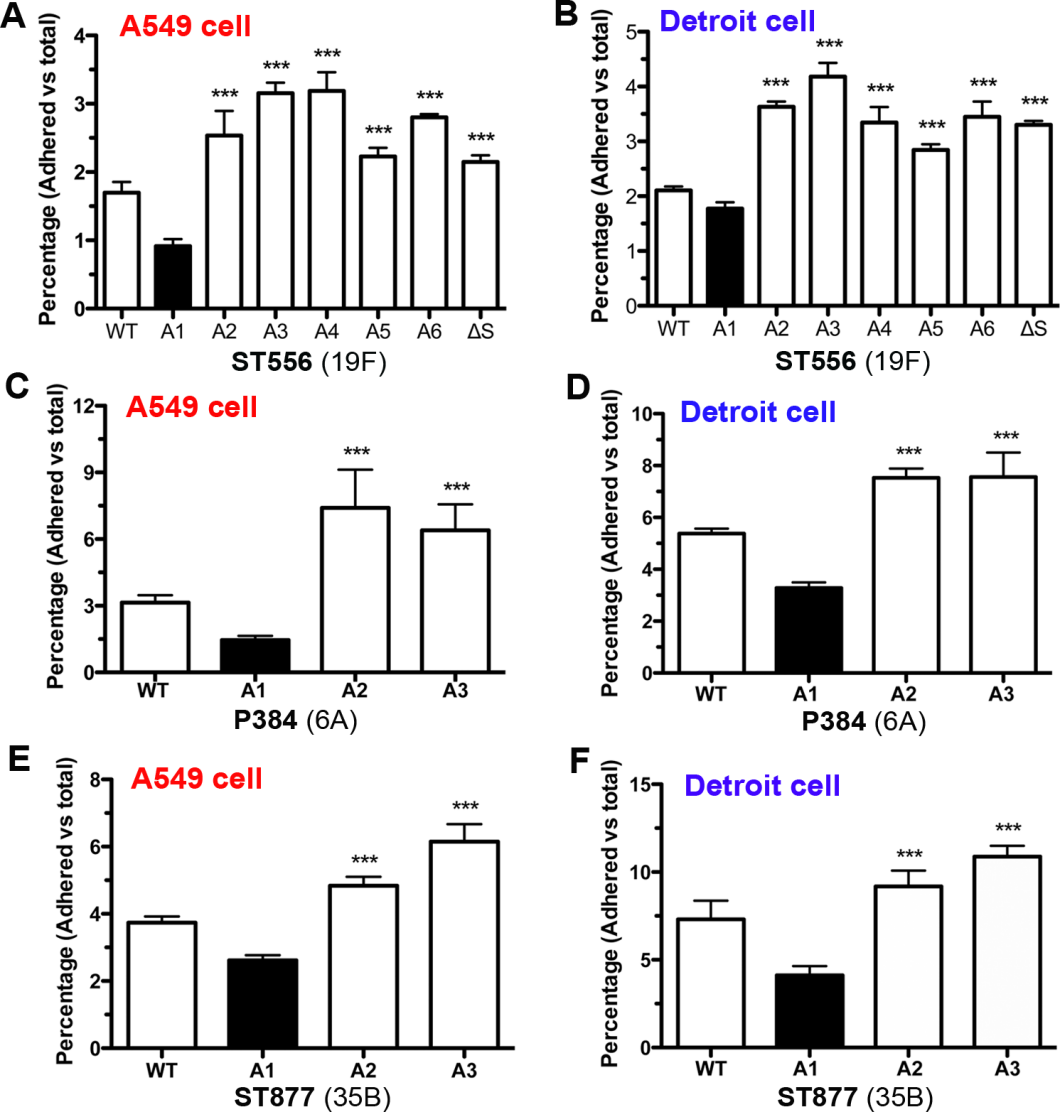
Significant impact of epigenetic-mediated phase variation on pneumococcal adhesion to host epithelial cells. The *hsdS*_*A1*_, *hsdS*_*A2*_, or *hsdS*_*A3*_ allele-carrying derivatives of strains ST556 (panels A and B), P384 (panels C and D), and ST877 (panels E and F) were cultured on the TSA plates supplemented with catalase as represented in Fig 6, and used to determine adhesion to human lung (A549 line) and nasopharyngeal (Detroit 562 line) cells in 24-well plates by counting CFU of adhering bacteria after extensive washing of the cell monolayers. The pneumococci carrying the *hsdS*_*A1*_ (A1) (producing opaque colonies) are significantly less adherent than those carrying the *hsdS*_*A2*_ (A2) or *hsdS*_*A3*_ (A3).

Previous studies show that the pneumococci producing opaque colonies are less adhesive to host epithelial cells than the transparent counterparts [31]. We thus tested the impact of the *hsdS*_*A*_ allelic variations on pneumococcal adhesion to host cells by incubating the ST556 derivatives each carrying one of the six *hsdS*_*A*_ alleles with human airway epithelial cells (lines A549-lung; Detroit 562-nasopharnx). Consistent with its opaque colony phenotype (Fig 6, TH5445), the strain carrying *hsdS*_*A1*_ (Fig 11A and 11B; A1) was significantly less adhesive than the transparent counterparts carrying the other *hsdS*_*A*_ alleles (A2-A6) in both cell models. The parent strain ST556 displayed an intermediate adhesion level (Fig 11A and 11B; WT), likely because the population was composed of both opaque and transparent pneumococcal variants due to the *hsdS* rearrangements in the *cod* locus. In the agreement with the OFF status of the A1 allele and its transparent phenotype (Fig 9A; TH6502), the *hsdS*-null mutant displayed substantially enhanced adhesion to host cells (Fig 11A and 11B; ΔS). The reduced adhesion of the *hsdS*_*A1*_-expressing pneumococci to host cells was reproducibly observed with the derivatives of other pneumococcal strains as exemplified with the type-6A P384 (Fig 11C and 11D) and type-35B ST877 (Fig 11E and 11F) strains. The adhesion experiments showed that *hsdS* gene inversion-driven epigenetic diversity results in colony opacity-dependent variations in pneumococcal adhesion to host epithelial cells.

### The epigenetic-driven phase variation diversifies the capacity of pneumococcal carriage in the upper respiratory tract

Significant differences among the *hsdS*_*A*_ allelic variants in adhesion capacity raised the possibility of intra-strain diversity in pneumococcal colonization in the upper respiratory tract because bacterial attachment to host epithelia is a critical requirement for successful colonization [47]. We tested this hypothesis with the *hsdS*_*A*_ allele-locked pneumococcal strains in a mouse competition carriage model. To avoid potential impact of selection markers on bacterial behavior *in vivo*, a PCR-based method was used to differentiate two *hsdS*_*A*_-specific pneumococcal strains in each mouse at the termination of the experiment.

**Fig 12:**
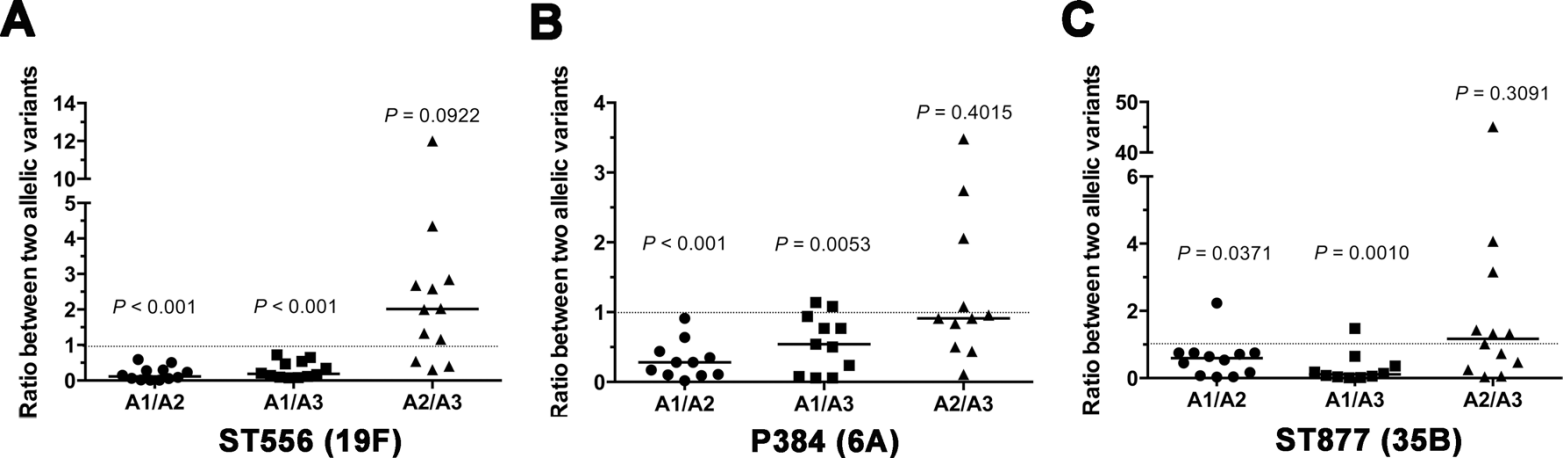
Significant impact of epigenetic-mediated phase variation on nasopharyngeal colonization of the pneumococci in the mouse co-carriage model. The pneumococcal derivatives of strains ST556 (A), P384 (B), and ST877 (C) each carrying the *hsdS*_*A1*_, *hsdS*_*A2*_, or *hsdS*_*A3*_ allele were grown on the TSA plates supplemented with catalase as represented in Fig 6. Two of the three unique *hsdS*_*A*_ allelic derivatives (A1, A2, and A3) from each strain background were mixed at a 1:1 ratio before being used to inoculate intranasally C57BL/6 mice. The colonizing pneumococci were recovered from each mouse by washing the nasal cavity 7 days post inoculation. The output ratio of the two the *hsdS*_*A*_ allele-specific variants co-infecting the same mouse was determined with the nasal lavage sample by PCR with the *hsdS*_*A*_ allele-specific primers. The *hsdS*_*A1*_-specific variant derived from each of three different strain backgrounds (A1) (forming opaque colonies) was less fit than the counterpart carrying *hsdS*_*A2*_ (A2) or *hsdS*_*A3*_ (A3) (forming transparent colonies) in the nasopharynx.

The co-carriage experiment revealed that the ST556 derivatives are substantially different in the ability to colonize the upper airway. The carriage level of the *hsdS*_*A1*_-specific pneumococci (TH5445) was significantly lower than those of its counterpart carrying *hsdS*_*A2*_ (TH5446, by 5.3 fold) or *hsdS*_*A3*_ (TH5447, by 3.3 fold)(Fig 12A). In contrast, the co-infection experiment did not show significant differences in the nasopharyngeal colonization between with the *hsdS*_*A2*_ (TH5446)- and *hsdS*_*A3*_ (TH5447)-specific strains. Similar patterns of differences in nasopharyngeal colonization were also observed between the *hsdS*_*A1*_- and *hsdS*_*A2*_/*hsdS*_*A3*_-locked pneumococci that were generated in the isogenic backgrounds of strain P384 (Fig 12B), and ST877 (Fig 12C). The relatively weaker colonization of the *hsdS*_*A1*_-specific pneumococci is fully consistent with their lower levels of adhesion to cultured epithelial cells (Fig 11), and with the previous observation that the transparent variants of *S*. *pneumoniae* have a fitness advantage than the opaque counterparts in the nasopharynx [33]. These data indicated that the epigenetic switches driven by the site-specific recombinations in the *hsdS* genes of the *cod* locus generate pneumococcal variants with variable capacity of nasopharyngeal carriage.

## Discussion

Bacterial R-M systems are well known for their function as an immune system against invasion of foreign DNA. DNA modifications catalyzed by the DNA methyltransferases of the R-M systems protect bacterial genomes from self-digestion by cognate restriction endonucleases. However, recent studies have indicated that the R-M systems impact bacterial biology beyond the immune function. The spontaneous ON/OFF mutations in the genes encoding the methyltransferases of type-III R-M systems lead to significant variations in the gene transcription in several pathogenic bacteria [48]. The site-specific recombinations in the *hsdS* genes of a type-I R-M system in *S*. *pneumoniae* result in variations in genome DNA methylation pattern, gene transcription, and virulence [27, 28]. These studies suggest that these R-M systems play a novel biological role(s) beyond their sole known function in stabilization of bacterial genomes. However, a causal relationship between the changes in the methylation (epigenetic) pattern and non-restriction phenotypes has not been established. Our data, for the first time, definitively demonstrate that the epigenetic patterns defined by the methyltransferase of a bacterial R-M system can fulfill an important biological function that is unrelated to the known R-M activity.

Phase variation between opaque and transparent colony phenotypes, first described in 1994 [33], is important for pneumococcal adaptation to different host niches and thereby pathogenesis [30, 31, 33, 49], but the molecular mechanism behind the phase variation remains undefined. This study has uncovered a novel epigenetic mechanism for this important mystery in pneumococcal pathobiology. First, we have demonstrated that the Spn556II type-I R-M (or *cod*) locus of *S*. *pneumoniae* determines the opaque and transparent colony phenotypes in all eight pneumococcal strains/serotypes tested in this work by gene knockout, complementation and phenotypic analysis. All wild type strains formed mixtures of opaque and transparent colonies, but the null mutants of the entire locus, *hsdM*, *hsdS*_*A*_ (one of the three *hsdS* genes) produced uniformly transparent colonies (Fig 9 and S9 Fig). Second, our data showed that the pneumococcal phase variation between the opaque and transparent colony phenotypes is determined by reversible ON-and-OFF switch of the *hsdS*_*A*_ A1 allele. The pneumococci carrying A1 (ON phase) uniformly produced opaque colonies, whereas the loss of A1 always led to the formation of transparent colonies in all eight pneumococcal strains tested thus far (Figs 6–9, S5–S7 Figs). Furthermore, expressing A1 in the strains with the transparent colony phenotype reverted the colony phenotype to the opaque phase (Fig 10). Third, N6-adenine methylation of motif 5’-CRAAN_8_CTT-3’ in pneumococcal genome by the A1-specific methyltransferase dictates the opaque colony phenotype. The results shown in Fig 9 and S9 Fig proved that the catalytic activity of the DNA methyltransferase defined by the A1 allele of *hsdS*_*A*_ is essential for the A1-dependent opaque colony phenotype. The loss-of-function mutations in either catalytic (HsdM) or sequence recognition (HsdS_A1_) subunit of the DNA methyltransferase completely abolish the opaque colony phenotype (Fig 9 and S9 Fig). Lastly, the tyrosine recombinase PsrA catalyzes one of the three inversions that control the ON/OFF switch of the *hsdS*_*A1*_ allele. We have demonstrated that the pneumococcal site-specific recombinase A (PsrA) catalyzes the DNA inversion between the *hsdS*_*A*_ and *hsdS*_*B*_ genes in two strains by gene knockout and complementation (Fig 5 and S2 Fig).

This inversion-driven epigenetic mechanism of pneumococcal phase variation represents the first case in which DNA inversion and epigenetic variation are combined to generate phenotypic switch. There are numerous well-characterized examples for the phase variation generated either by DNA inversion or epigenetic switch [50, 51]. These are best represented by phase variations in the *Salmonella* flagellum [52–54] and the *E*. *coli* pyelonephritis-associated pilus [6, 7]. However, to our best knowledge, there has been no well-documented phase variation that utilizes both the mechanisms of DNA inversion and methylation. DNA inversion and methylation are relatively simple biochemical reactions, which minimally requires only two unique components (a recombinase and two inverted repeats for inversion; a methyltransferase and a target sequence for methylation). An apparent constraint of both the DNA inversion and methylation-driven phase variation is that each mechanism alone can only target limited number of genes as exemplified by the Hin invertase-catalyzed switch of the *Salmonella* flagellum [52–54] and the Dam methylase-mediated ON/OFF phase variation in the *E*. *coli* pyelonephritis-associated pilus [6, 7]. As shown in this study, DNA inversion in the type-I R-M *hsdS* genes effectively combines the biochemical simplicity of inversion reaction with the global nature of the DNA methylation catalyzed by the R-M DNA methyltransferases, which typically methylate a large number of sites in microbial genomes. This combinatorial mode of action may allow phenotypic switches in the biological structures/properties that are defined by multiple but physically unrelated genes or loci. Along this line, it is reasonable to believe that the pneumococcal phase variation in colony opacity is defined by a highly coordinated action of multiple genetic elements although the actual mechanism remains to be defined.

It is intriguing that the ON/OFF phases of the HsdS_A1_-specific epigenetic status are controlled by multiple site-specific recombinases. As illustrated in Fig 3A, the *hsdS*_*A1*_ allele can be switched ON or OFF by three different DNA inversions (e.g. A1-A2, A1-A3, and A1-A4). Previous studies hypothesize that the recombinase encoded by *psrA* in the *cod* locus drives the site-specific recombinations among the three adjacent *hsdS* genes [26–28]. However, the recombination mechanisms have not been experimentally characterized. Our result showed that tyrosine recombinase PsrA only catalyzes the A1-A2 inversion between the *hsdS*_*A*_ and *hsdS*_*B*_ (or DNA configurations S1 and S2), but not the inversions between *hsdS*_*A*_ and *hsdS*_*C*_. Thus, an unknown recombinase(s) must catalyze the DNA inversions and excisions between *hsdS*_*A*_ and *hsdS*_*C*_, particularly the A1-A3 and A1-A4 inversions associated with the phase variation. The complex nature of the inversions in the *cod* locus is reminiscent of the inversions that mediate the type-I pilus phase variation in *E*. *coli*. The inversion in the promoter sequence of *fimA*, the structural gene of type-I pilin, is catalyzed by two independent tyrosine recombinases, FimB and FimE [55]. While FimB drives inversion in both directions, FimE catalyzes the reaction from the “ON” to the “OFF” orientation [56, 57]. Furthermore, the inversion frequency of the *fimA* promoter is modulated by temperature, media, sialic acid, and nutrient availability [58–60]. In this context, it is possible that the phase variation and other potentially unknown consequence(s) brought about by the site-specific recombinations in the *cod* locus may be subject to multiple regulations, enabling the pneumococci to produce epigenetic/phenotypic variants in response to different intracellular and/or extracellular conditions. This hypothesis is consistent with the previous finding that the expression of *psrA* is significantly higher in an *spxB* mutant [61]. SpxB, a pyruvate oxidase, is a central player in pneumococcal carbohydrate metabolism by generating the phosphoryl donor metabolite acetyl-phosphate and hydrogen peroxide [62]. We are in the process of determining how metabolic status of *S*. *pneumoniae* modulates the expression of *psrA* and site-specific recombinations in the *cod* locus.

By using the epigenetic/phase-locked strains, our cell adhesion and nasopharyngeal carriage experiments have recapitulated some of the important phenotypic properties associated with the opacity variants [29]. As compared with the non-A1 (transparent) variants, the A1-carrying (opaque) pneumococci displayed significantly lower levels of epithelial adhesion in both human lung (A549 line) and nasopharyngeal (Detroit 562 line) cells (Fig 11). This result is consistent with the inferior nasopharyngeal carriage of the A1-carrying pneumococci in the adult mouse model (Fig 12). These characteristics of the epigenetic variants are fully consistent with the previous observations that the opaque and transparent variants have their own unique superiority in nasopharyngeal carrier (for the transparent) or evasion of phagocytic killing during invasive infections systemic infection (for the opaque) [30, 31, 33, 49].

It is known that unencapsulated pneumococci undergo spontaneous phase variation in colony opacity [29], but this is the first report of phase variation in phase-locked unencapsulated strains. This work showed that the capsule is not required for pneumococcal variation. The unencapsulated mutants of D39 (serotype 2), TIGR4 (serotype 4), and ST556 (serotype 19F) displayed the same colony phenotypes as their encapsulated parents in an *hsdS*_*A1*_ allele-dependent manner (Fig 8 and S7 Fig). It should be emphasized that certain capsule types (e.g. 2 and 4) can blur the morphologic difference between opaque and transparent colonies as described previously [29]. We were unable to differentiate opaque and transparent colonies of D39 and TIGR4 derivatives until their original capsules were removed (Fig 8 and S7 Fig) or replaced with a type-19F capsule (Fig 7). The obscure colony phenotypes of D39 may explain several key discrepancies between our data and some of the results recently reported by Manso *et al*. [28]. The D39 derivative with a locked allele A3 of *hsdS*_*A*_ (SpnIIIA in reference [28]), was described to form 100% opaque colonies by Manso *et al*. [28]. However, this allele was always associated with the transparent colonies in the eight strains tested in this work (Figs 6–8). In addition, we could not substantiate the previous report that the pneumococci carrying the *hsdS*_*A*_ alleles A2 (SpnIIIB), A4 (SpnIIID), A5 (SpnIIIC), and A6 (SpnIIIF) form mixtures of opaque and transparent colonies [28]. These alleles consistently yielded transparent colonies in all eight strains tested in this work (Figs 6–10). Opaque colonies were occasionally observed with some of the non-A1 carrying strains, but the sequencing analysis indicated that they were caused by mutations in the *spxB* gene as documented previously [39]. Because both the studies apparently used the same experimental conditions to visualize pneumococcal colonies as described by Weiser *et al*. [33], and our data were repeated in multiple pneumococcal strains, we believe that some of the colony morphology results reported in the previous study [28] were incorrectly interpreted. This might be due to marginal differences between the opaque and transparent colonies of the D39 derivatives (with the original type-2 capsule).

There is much more to be learnt from the DNA rearrangements in this type-I R-M locus, in terms of the functional implications for the allelic variants of *hsdS*_*A*_, *hsdS*_*B*_, and *hsdS*_*C*_, which are generated by DNA inversion and excision events. While we identified a total of nine *hsdS*_*A*_ alleles, only the *hsdS*_*A1*_ allele has a defined role in the formation of pneumococcal opaque colony variant. Because our SMRT sequencing data showed that the other five *hsdS*_*A*_ (*hsdS*_*A2-6*_) alleles are functional in DNA methylation (Fig 4), it is reasonable to postulate that these methylase variants may contribute to additional phenotypic diversity of *S*. *pneumoniae* in the aspects that were not characterized in this study. The pneumococci carrying the *hsdS*_*A*_ alleles derived from the DNA excisions between *hsdS*_*A*_ and *hsdS*_*C*_ (*hsdS*_*A7*_, *hsdS*_*A8*_, or *hsdS*_*A9*_) did not show detectable allele-specific methylation signals in the SMRT sequencing data (Fig 4), indicating that the corresponding HsdS variants are no longer functional in the methylase activity. Because the HsdS subunits are also required for the endonuclease activity of the HsdR subunit [63], it is likely that these truncated *hsdS* alleles are also inactive in restriction activity. The DNA rearrangements in the type-I R-M loci of *M*. *pulmonis* also lead to the loss of the R-M activity [17]. This result thus challenges the current paradigms on the sole function of bacterial R-M systems as an immune system to confer protection from invasion of foreign DNA. Although it is puzzling why this bacterium possesses the DNA excision mechanism in the *cod* locus, we cannot rule out the possibility that the truncated HsdS variant proteins fulfill a non-R-M function.

The findings of this study have a broad biological implication in bacterial epigenetic and phenotypic diversification because similar recombination systems widely exist in the type-I R-M systems of the prokaryotic organisms. Besides the well-characterized *hsdS* recombinations in *M*. *pulmonis* [17], our recent search of the genome sequence databases identified homologs of the Spn556II recombination system (e.g., multiple *hsdS* and recombinase *psrA* genes) in many archaeal and bacterial species, representing a broad phylogenetic spectrum of bacteria, such as *Mycoplasma* (e.g., *Mycoplasma mycoides* and *Mycoplasma bovis*), Spirochetes (e.g., *Treponema medium* and *Treponema denticola*), Gram-negative bacteria (e.g., *Bacteroides fragilis* and *Campylobacter upsaliensis*), and Gram-positive bacteria (e.g., *Enterococcus faecalis* and *Streptococcus agalactiae*). Among these include many pathogenic bacteria, such as *B*. *fragilis*, *E*. *faecalis*, *Enterococcus faecium*, *S*. *agalactiae*, and *Streptococcus suis*. High conservation of this recombination system strongly suggests that DNA rearrangements of the *hsdS* genes widely occur in the prokaryotic organisms. In fact, DNA rearrangements in the *hsdS* genes have been previously observed in *B*. *fragilis* during the genome assembly from cloned DNA segments [64]. Based on our data in this work, it is reasonable to predict that site-specific recombinations in the DNA methyltransferase *hsdS* genes of the type-I R-M systems may serve an important function(s) in promoting epigenetic and phenotypic diversity among the prokaryotic organisms.

## Materials and Methods

### Bacterial strains, cultivation, and chemical reagents

The bacterial strains used in this study are described in S2 Table. The pneumococci were grown in Todd-Hewitt broth with 5% yeast extract (THY) or on tryptic soy agar (TSA) plates with 3% defibrinated sheep blood at 37°C with 5% CO_2_ as described [65], unless otherwise specified. *E*. *coli* DH5α was used for DNA cloning as described [66]. The restriction enzymes and DNA polymerases were purchased from New England Biolabs (NEB, Beijing, China). All ingredients for bacterial culture media and other chemicals were supplied by Sigma (Shanghai, China) unless otherwise indicated.

### Construction of pneumococcal mutants

All mutagenesis works in *S*. *pneumoniae* were carried out by natural transformation essentially as described [67]. The relevant mutant strains, primers, and mutagenesis procedures (e.g., PCR amplification, parental strains, and genotypes of resulting strains) are described in S2, S3, and S4 Tables. The unmarked deletions were constructed in streptomycin-resistant derivatives ST606 (of ST556) and ST1759 (of TIGR4) by counter selection as described [68]. The mutants TH5792 (of strain ST556) and TH5794 (of strain TIGR4) lacking the entire *hsdS* region were constructed in two steps. First, the up- and down-stream regions were amplified from genomic DNA samples of the wild type strains, respectively. The resulting DNA products were digested with XbaI and XhoI, and ligated to the XbaI/XhoI-digested Janus cassette (JC) that was amplified with primers Pr1097 and Pr1098 from strain ST588 [67], and transformed into ST606 and ST1759 respectively, resulting in the JC replacement strains TH5791 (for ST556) and TH5793 (for TIGR4). Second, the unmarked deletion was generated by replacing the JC marker with the desirable sequences. The up- and down-stream sequences of the *hsdS* region were amplified from either ST556 or TIGR4, and linked by fusion PCR as described [69]. The fused DNA fragments were transformed into TH5791 or TH5793, resulting in strains TH5792 (ST556∆*hsdS*_*A-C*_) and TH5794 (TIGR4∆*hsdS*_*A-C*_). The other unmarked deletions in pneumococcal genes were made by the same procedures. The *hsdS*_*A*_ and *psrA* JC-replacement mutants TH6501 (ST606∆*hsdS*_*A*_::JC), TH6500 (ST1759∆*hsdS*_*A*_::JC), TH5993 (ST606∆*psrA*::JC), and TH6525 (ST1759∆*psrA*::JC) were generated and used to produce unmarked deletion mutants TH6502 (TH6501**∆** *hsdS*_*A*_), TH6012 (TH5993∆*psrA*), and TH6555 (TH6525∆*psrA*) as described in S4 Table.

The *hsdS*_*A*_ allele-locked strains in ST556 were constructed by amplifying the up- and down-stream sequences from the genomic DNA or cloned PCR products with appropriate *hsdS*_*A*_ alleles as described above. Some of the strains were generated in strain TH5993 with the fusion PCR products of two amplicons using the terminal primers: TH5445 (invariable *hsdS*_*A1*_), TH5446 (invariable *hsdS*_*A2*_), TH5447 (invariable *hsdS*_*A3*_), TH5451 (invariable *hsdS*_*A7*_), TH5452 (invariable *hsdS*_*A8*_), and TH5453 (invariable *hsdS*_*A9*_). The sequences of the other *hsdS*_*A*_ alleles and their flanking sequences were separately amplified with three pairs of primers before being used to perform fusion PCR and transformation of TH5993 as listed in S4 Table.

Due to high false positive rates with the original version of JC in the counter selection step, we engineered a new Janus cassette (referred to as JC1) to establish *hsdS*_*A*_ allele-locked strains hereafter. JC1 was established by replacing the original promoter sequence of JC with a stronger promoter of elongation factor Tu (EF-Tu, MYY1418), which was amplified from the genomic DNA of ST556 by primers Pr9840 and Pr9396. The *kan-rpsL* coding sequences in the original JC [68] was amplified by primers Pr9395 and Pr1098, and linked to the downstream of the EF-Tu promoter amplicon by fusion PCR with primers Pr9840 and Pr1098. The JC1 amplicon was digested with XbaI and XhoI, and ligated to the XbaI/XhoI-digested the up- and down-stream amplicons that were originally amplified from genomic DNA of each target strain with primer pairs Pr10103/Pr10104 and Pr10105/Pr10106. The ligated DNA fragments were transformed into streptomycin-resistant derivatives of D39 (TH4306), TIGR4 (ST1759), P384 (TH6671), TH2901 (TH7562), TH2835 (TH7556), TH2886 (TH7560) and ST877 (TH6675), to generate the *hsdS*-region replacement strains TH7457 (TH4306∆*hsdS*_*A-C*_::JC1), TH7454 (ST1759∆*hsdS*_*A-C*_::JC1), TH7187 (TH6671∆*hsdS*_*A-C*_::JC1), TH7574 (TH7562∆*hsdS*_*A-C*_::JC1), TH7568 (TH7556∆*hsdS*_*A-C*_::JC1), TH7572 (TH7560∆*hsdS*_*A-C*_::JC1) and TH7191 (TH6675∆*hsdS*_*A-C*_::JC1). These strains were then used to construct the corresponding *hsdS*_*A*_ allele-locked strains by replacing JC1 with the appropriate sequences of the *hsdS*_*A*_ alleles through counter selection as described above. The coding sequences of *hsdS*_*A1*_, *hsdS*_*A2*_, *hsdS*_*A3*_, *hsdS*_*A4*_, *hsdS*_*A5*_ and *hsdS*_*A6*_ were amplified from genomic DNA of TH5445, TH5446, TH5447, TH5450, TH5449 and TH5448 using primers Pr10108/Pr10109, and linked to flanking sequences of the Spn556II *hsdS* region by fusion PCR. The up- and down-stream regions of the *hsdS* region were amplified from genomic DNA sample of each target strain. The resulting fusion PCR amplicons were transformed into the corresponding JC1-replacement derivatives to generate single *hsdS*_A_ allele-locked strains.

A similar procedure was used to construct the capsule switch mutants. The up- and down-stream regions of the capsule locus were amplified from the genomic DNA of ST606 and TH4306, digested by XbaI or XhoI, ligated to JC1, and transformed into ST606 and TH4306, resulting in unencapsulated strains TH7901 (for D39) and TH8160 (for ST556). The type-2 or -19F capsule operons and their flanking regions were amplified from the genomic DNA of ST556 or D39, and transformed into TH7901 (TH4306∆*cps*::JC1) and TH8160 (ST606∆*cps*::JC1), respectively, resulting in capsule switch mutants TH7903 (D39^cps19F^) and TH8162 (ST556^cps2^). The *hsdS*_*A*_ allele-locked strains in TH7903 (D39^cps19F^) were constructed in a similar manner. Strain TH7908 (TH7903∆*hsdS*_*A-C*_::JC1) was prepared by amplifying the genomic DNA of TH7457 (TH4306∆*hsdS*_*A-C*_::JC1) using primer pairs Pr8055/Pr8058 and transforming the amplicon into strain TH7903. The *hsdS*_*A*_ alleles and their flanking DNA sequences were amplified from genomic DNA of *hsdS*_*A*_ allele-locked D39 derivatives with primers Pr10103 and Pr10106, and transformed into the TH7908 (TH7903∆*hsdS*_*A-C*_::JC1), resulting in strains TH7910 (TH7903∆*hsdS*_*A-C*_::*hsdS*_*A1*_), TH7913 (TH7903∆*hsdS*_*A-C*_::*hsdS*_*A2*_), TH7916 (TH7903∆*hsdS*_*A-C*_::*hsdS*_*A3*_), TH8154 (TH7903∆*hsdS*_*A-C*_::*hsdS*_*A4*_), TH8156 (TH7903∆*hsdS*_*A-C*_::*hsdS*_*A5*_), TH8158 (TH7903∆*hsdS*_*A-C*_::*hsdS*_*A6*_). Similarly, strain TH8164 (TH8162∆*hsdS*_*A-C*_::JC1) was generated by transforming the DNA fragments from PCR amplification of ∆*hsdS*_*A*_ *-*_*C*_::JC1 region in TH5791 (ST606) using primers Pr8055 and Pr8058. The *hsdS*_*A*_ allele-locked strains TH8166 (TH8164∆*hsdS*_*A-C*_::*hsdS*_*A1*_), TH8168 (TH8164∆*hsdS*_*A-C*_::*hsdS*_*A2*_), TH8170 (TH8164∆*hsdS*_*A-C*_::*hsdS*_*A3*_), TH8172 (TH8164∆*hsdS*_*A-C*_::*hsdS*_*A4*_), TH8174 (TH8164∆*hsdS*_*A-C*_::*hsdS*_*A5*_), TH8176 (TH8164∆*hsdS*_*A-C*_::*hsdS*_*A6*_) in the TH8162 (ST556^cps2^) background were constructed by replacing JC1 in TH8164 (TH8162∆*hsdS*_*A-C*_::JC1) by transformation with the appropriate amplicons from the *hsdS*_*A*_ allele-locked ST556 derivatives using primers Pr8055 and Pr8058, respectively.

The *hsdS*_*A*_ allele-locked derivatives of the unencapsulated ST556, D39 and TIGR4 were constructed by amplifying the up- (with primers Pr10489/Pr11159) and down-stream (with Pr10491/Pr11160) sequences of the capsule operons, digesting the amplicon with XbaI and XhoI, ligating with XbaI/XhoI digested JC1, and transforming the ligation product into derivatives of ST556: TH5445 (ST606∆*hsdS*_*A*_ *-*_*C*_::*hsdS*_*A1*_), TH5446 (ST606∆*hsdS*_*A*_ *-*_*C*_::*hsdS*_*A2*_), TH5447 (ST606∆*hsdS*_*A*_ *-*_*C*_::*hsdS*_*A3*_); D39: TH7443 (TH4306∆*hsdS*_*A*_ *-*_*C*_::*hsdS*_*A1*_), TH7446 (TH4306∆*hsdS*_*A*_ *-*_*C*_::*hsdS*_*A2*_), TH7449 (TH4306∆*hsdS*_*A*_ *-*_*C*_::*hsdS*_*A3*_); TIGR4: ST1759 (TIGR4::*rpsL1*), TH7434 (ST1759 ∆*hsdS*_*A*_ *-*_*C*_::*hsdS*_*A1*_), TH7437 (ST1759 ∆*hsdS*_*A*_ *-*_*C*_::*hsdS*_*A2*_), TH7440 (ST1759 ∆*hsdS*_*A*_ *-*_*C*_::*hsdS*_*A3*_), resulting in unencapsulated mutants of ST556 (TH8178, TH8180, and TH8182), D39 (TH8184, TH8186, and TH8188), and TIGR4 (TH8190, TH8192, TH8194, and TH8196).

The unmarked deletion mutants in the *hsdRMS* genes of the Spn556II R-M system were similarly constructed in the ST556 and TH5445 strain backgrounds. The JC1-replacement strain TH6111 (ST606∆*hsdR*::JC1) and TH7912 (TH5445∆*hsdR*::JC1) were generated by amplifying the upstream sequence (with primers Pr8047 and Pr8048), JC1 (with Pr9840 and Pr1098), and downstream sequence (with Pr8049 and Pr8050), digesting the amplicons with XbaI/XhoI, and transforming the ligation product into strain ST606 and TH7912, respectively. The unmarked deletion mutant TH6112 (TH6111∆*hsdR*) and TH7915 (TH7912∆*hsdR*) was subsequently made by amplifying the flanking sequences of *hsdR* with primer pairs Pr8047/Pr8798 and Pr8797/Pr8050, linking the amplicons by fusion PCR with the terminal primers, transforming strain TH6111 and TH7912 with the fusion PCR product, and counter selecting for streptomycin-resistant/kanamycin-sensitive transformants. Strain TH5914 (ST606∆*hsdRM*::JC1) and TH7925 (TH5445∆*hsdRM*::JC1) lacking the *hsdR* and *hsdM* were derived by transformation of ST606 and TH5445 with the ligated products of JC1 and the XbaI/XhoI-digested flanking sequences (amplified with primer pairs Pr8047/Pr8048 and Pr8053/Pr8054), respectively. These strains were in turn used to prepare TH6113 (TH5914∆*hsdRM*) and TH7928 (TH7925∆*hsdRM*) by transformation of strain TH5914 and TH7925 with the fusion PCR product of the flanking amplicons (Pr8047/Pr8800 and Pr8799/8054), respectively. Strain TH5444 (TH4992∆Spn556II) was generated with the fusion PCR product of the flanking amplicons (Pr6930/Pr7956 and Pr7955/Pr7953) from strain TH4992 (ST606∆Spn556II::JC).

Strain TH5915 was prepared in TH6112 by replacing *hsdM* with JC1 using primer pairs Pr8771/Pr8772, Pr8773/Pr8774, and Pr9840/Pr1098. Complementation of *hsdM* was carried out by replacing JC1 in TH5915 (TH6112∆*hsdM*::JC1) through transformation with the Pr8771/Pr8774 amplicon (wild type *hsdM*, TH6115), fusion PCR product of the Pr8771/Pr8816 and Pr8815/Pr8774 amplicons (for E228A *hsdM*, TH6116), or fusion PCR product of the Pr8771/Pr8818 and Pr8817/Pr8774 amplicons (for N255A *hsdM*, TH6117) from the genomic DNA of strain TH6112. The similar procedure was used to construct TH7918 (TH7915∆*hsdM*::JC1), TH8199 (TH7918::*hsdM*), TH8200 (TH7918::*hsdM* ^E228A^) and TH8201 (TH7918::*hsdM* ^N255A^) in the TH5445 strain background.

Complementation strains TH6659 and TH6669 of *psrA* were prepared by transforming TH5993 (ST606∆*psrA*::JC) and TH6525 (TH1759∆*psrA*::JC) with the Pr7602/Pr7567 amplicon representing the *psrA* and its flanking sequences.

The strains co-expressing two same or different alleles of *hsdS*_*A*_ were similarly generated in the ST556 background. Strains TH7919 (TH5445∆*bgaA*::JC1), TH7921 (TH5446∆*bgaA*::JC1), and TH8208 (TH5447∆*bgaA*::JC1) were constructed by replacing the partial *bgaA* coding sequence with JC1 using primer pairs Pr8227/Pr8228, Pr9840/Pr1098, Pr8229/Pr8230. The *hsdS*_*A*_-locked alleles (*hsdS*_*A1*_, *hsdS*_*A2*_ or *hsdS*_*A3*_) were amplified from the genomic DNA of TH5445 (TH5993∆*hsdS*_*A*_ *-*_*C*_::*hsdS*_*A1*_), TH5446 (TH5993∆*hsdS*_*A*_ *-*_*C*_::*hsdS*_*A2*_), and TH5447 (TH5993∆*hsdS*_*A*_ *-*_*C*_::*hsdS*_*A3*_) with primers Pr11163 and Pr11164, respectively. The flanking regions of *bgaA* and promoter of the Spn556II locus were amplified from the genomic DNA of ST556 with Pr11165/Pr11166, digested with BsaI, and ligated with BsaI-digested amplicon of the *hsdS*_*A*_-locked alleles A1, A2, or A3. The ligation products were transformed into TH7919 (TH5445∆*bgaA*::JC1), TH7921(TH5446∆*bgaA*::JC1), and TH8208 (TH5447∆*bgaA*::JC1), resulting in TH7923 (TH7919∆*bgaA*::*hsdS*_*A1*_), TH7926 (TH7919∆*bgaA*::*hsdS*_*A2*_), TH8145 (TH7919∆*bgaA*::*hsdS*_*A3*_), TH7929 (TH7921∆*bgaA*::*hsdS*_*A1*_), TH7932 (TH7921∆*bgaA*::*hsdS*_*A2*_), TH8148 (TH7921∆*bgaA*::*hsdS*_*A3*_), TH8118 (TH8208∆*bgaA*::*hsdS*_*A1*_), TH8151 (TH8208∆*bgaA*::*hsdS*_*A2*_), and TH8121 (TH8208∆*bgaA*::*hsdS*_*A3*_).

### Microscopic observation and quantification of pneumococcal colony variants

Microscopic visualization of pneumococcal colony opacity was carried out with TSA plates supplemented with catalase (Sigma) as described [33]. Briefly, *S*. *pneumoniae* strains were cultivated in THY to an optical density at 620 nm (OD_620_) of 0.5 at 37°C with 5% CO_2_ as described before. The bacterial culture was stored in THY with 15% glycerol (v/v) at -80°C. After the concentrations of viable bacteria in the frozen stocks were determined by counting the number of colonies on blood plates, the frozen stocks were diluted to approximately 10^4^ colony forming unit (CFU)/ml in phosphate-buffered saline (PBS), mixed with equal volume (100 μl each) of catalase (60,000 units/ml), and spread on TSA plates without blood. The plates were incubated for 16 or 24 hours before microscopic visualization of pneumococcal colonies. Colony morphology was observed and photographed under a dissection microscope with a substage illuminator and an angle-adjusting mirror.

Phenotypic stability of pneumococcal strains in colony opacity was assessed by relative ratio between the opaque and transparent colonies produced from a single seeding colony essentially as described above. Initially, frozen stocks were spread on fresh TSA agar plates without blood to generate well-separated seeding colonies. The colonies with either opaque or transparent phenotype were resuspended in 100 μl PBS, diluted to approximately 2,000 CFU/ml in PBS, mixed with equal volume (100 μl each) of the catalase solution, and spread on fresh TSA plates without blood. All colonies on each plate were microscopically assigned to either opaque or transparent phenotype 16 hours post inoculation. Three different colonies with the same phenotype in each strain were tested when available.

### DNA amplification, cloning, and sequencing

PCR was carried out with a high fidelity PrimeSTAR DNA polymerase (TaKaRa, Dalian, China) described [70]. The primers used in this study are described in S3 Table. The entire *hsdS* region of the *cod* locus was amplified by PCR from genomic DNA of ST556 with primers Pr7676 and Pr7677, and cloned in TA cloning vector pCR2.1 (Invitrogen, Beijing, China) in *E*. *coli* as instructed by the supplier. The resulting colonies were randomly picked to detect the inserts and their sizes by colony PCR [71]. The amplicons from the PCR-positive colonies were sequenced using vector-based primers Pr7651 and Pr7652, and compared with the genome sequence of ST556 (accession CP003357). The sequences of the allelic variants in the *cod* locus identified by this approach (as summarized in Fig 3) were deposited to the NCBI GenBank under the following accession numbers: KU313683 (S1), KU321882 (S2), KU321883 (S3), KU321884 (S4), KU321885 (S5), KU321886 (S6), KU321887 (S3’), KU321888 (S4’), S7: KU321889 (S7), KU321890 (S8), and KU321891 (S9).

The single molecule real-time (SMRT) sequencing was carried out in the W. M. Keck Foundation Biotechnology Resource Laboratory at Yale University as described in our previous study [35]. The SMRT sequencing data of the ST556 whole genome amplification (WGA) from our previous study [35] were used as a methylation negative control for bioinformatic identification of methylation sites from the native (unamplified) genomic DNA. Rho-independent transcription terminators were identified by the internet-based ARNold program at http://rna.igmors.u-psud.fr/toolbox/arnold/index.php#Results. The raw data represented in Fig 4 are available at the NCBI under the following accession numbers: SRX1757519 (ST556), SRX1752589 (TH5445), SRX1752590 (TH5446), SRX1752592 (TH5447), SRX1752599 (TH5448), SRX1752600 (TH5449), SRX1752601 (TH5450), SRX1752602 (TH5451), SRX1752603 (TH5452), and SRX1752604 (TH5453).

The relative representation of the *hsdS*_*A*_ allelic variants in the ST556 population used for the SMRT sequencing was estimated by individually aligning the sequences of S1-S9 with adaptor trimmed SMRT sequencing data by MUMmer (version 3.23) [72]. The parameter “nucmer-l 10” was used to run MUMmer. For each allele, reads that span all the variable regions are referred to as supporting reads. Proportion of each allele was calculated by the following formula: allele proportion = number of supporting reads of the allele/total number of supporting reads of all the alleles. The raw data for analysis of allele proportion is available at the NCBI under the accession: SRX1757517.

### Determination of restriction activity

The genetic transformation efficiency was used to assess the restriction activity of pneumococcal strains as described [73]. The methylation motif-specific donor plasmids were constructed in the pIB166 shuttle vector [74]. The DNA sequences (~500 bp) containing five copies of the methylation motif for *hsdS*_*A1*_ (5’-CRAAN_8_CTT-3’), *hsdS*_*A2*_ (5’-CRAAN_9_TTC-3’), or *hsdS*_*A3*_ (5’-CRAAN_8_CTG-3’) were identified from different loci of the ST556 genome based on the SMRT sequencing data (Fig 4), amplified using primer pairs Pr9576/Pr9577 (*hsdS*_*A1*_), Pr9578/Pr9579 (*hsdS*_*A2*_), and Pr9780/Pr9781 (*hsdS*_*A3*_), digested with ScaI/HindIII, and ligated to the ScaI/HindIII-digested amplicon of pIB166 (with primers Pr10598/Pr10599), resulting in pTH7223 (*hsdS*_*A1*_), pTH7224 (*hsdS*_*A2*_), and pTH7225 (*hsdS*_*A3*_). A DNA fragment without any of the three methylation motifs was amplified from genomic DNA of ST556 with primers Pr9785 and Pr9786 as a negative control (pTH7222). Equal amount of the recombinant plasmids (500 ng/ml) isolated from a DNA-methyltransferase-deficient *E*. *coli* strain ER2796 [75], was used to transformed ST556 or its *hsdS*_*A*_ allele-locked derivatives by standard natural transformation method [76] to determine the transformation efficiency by enumerating the number of chloramphenicol-resistant colonies for each combination of strain and donor plasmid. The ratio between the numbers of the transformants and total pneumococcal cells is used to represent the transformation efficiency or restriction activity of each strain upon transformation with one of the donor plasmids. The data are presented as means of triplicate reactions ± standard deviations.

### Assessment of DNA methyltransferase activity

The activity of the type-I R-M methyltransferase was determined by restriction digestion with methylation-sensitive restriction enzymes as described [77]. The plasmid (pTH4832, pRRS::*hsdM*) carrying *hsdM* and *hsdS*_*A*_ of Spn556II and the recognition DNA sequence 5’-CAAAAAAAAGTACTT-3’ of *hsdS*_*A*_ was used as described [35]. The 228^th^ glutamate and 255^th^ asparagine, two residues essential for the DNA methylase activity of HsdM was mutated to alanine as described [78]. Briefly, the two mutations were introduced by PCR amplification of TH4832 plasmid (pRRS::*hsdM*) with the primer pairs Pr8815/Pr8816 and Pr8817/Pr8818, and transformation of *E*. *coli* ER2796, resulting in plasmids pTH8221 (pRRS::*hsdM* ^E228A^) and pTH8222 (pRRS::*hsdM* ^N255A^). As an activity-negative control, plasmid pTH4836 containing frame-shifted methyltransferase (pRRS::frame-shifted *hsdM*) was used as described [35]. To detect methylation status of the sequence motifs, the restriction digestion reactions were performed as described [35].

### Host cell adhesion assay

The cell lines A549 and Detroit 562 were obtained from American Type Culture Collection (Manassas, VA). They were used to determine the adhesion capacity of pneumococcal strains essentially as described [79]. Four wells each with a confluent monolayer of A549 cells in 24-well plates were individually infected with approximately 10^7^ pneumococci of each strain, centrifuged at 1,000 RPM for 5 min, and incubated for 1 hr at 37°C. The adherent pneumococci were determined by enumerating the number of bacterial colonies after washing off the non-adherent bacteria with PBS, lysis of the monolayers with cold 0.025% Triton X-100, and spread of the lysates on TSA blood plates. The pneumococci used for the adhesion assay were collected by washing the colonies from the catalase-supplemented TSA plates with F12-K medium (for A549) or Eagle’s Minimum Essential Medium (for Detroit 562). The colonies were prepared as described above in the microscopic observation section. Each experiment was repeated at least three different times. The results of representative experiments are presented as means of four replicates ± standard deviations.

### Nasopharyngeal carriage

Pneumococcal nasopharyngeal carriage was determined in co-infection mouse model as described [65]. Groups of 12 female C57BL/6 mice (6–8 weeks old, Vital River, Beijing, China) were intranasally inoculated with a sub-lethal dose of each two-strain mixture at 1:1 ratio in 10 μl sterile Ringer’s solution (ST556: 10^7^ CFU, P384: 10^6^ CFU, ST877: 10^7^ CFU). The mice were sacrificed by cervical dislocation to collect nasal lavages and obtain colonies on the TSA blood plates 7 days post infection. The pneumococcal inocula for all co-infection experiments were prepared with the colonies from the catalase-supplemented TSA plates as described in Fig 6. The colonies of each *hsdS*_*A*_-specific strain were initially resuspended in sterile Ringer’s solution followed by diluting to approximately the same levels of viable pneumococcal according to the ratio between the value of OD_620_ and the CFU for each strain predetermined by the pilot experiments. The two composite strains used in the same inoculum were mixed at a 1:1 ratio in terms of CFUs immediately before the infection experiment. A fraction of each inoculum of two composite strains was plated on blood dishes to determine the actual CFU values. The ratio between the two composite strains in each mouse was determined among 48 randomly selected colonies obtained with each of the nasal lavage samples by multiplex PCR using a combination of the common primer Pr10583 with the *hsdS*_*A*_ allele-specific primers Pr10584 (for *hsdS*_*A1*_), Pr10585 (for *hsdS*_*A2*_), or Pr10586 (for *hsdS*_*A3*_). The results are presented as the colony ratio between the two *hsdS*_*A*_ allelic variants in the output sample from the same mouse.

### Statistical analysis

Statistical comparisons of incidence were performed using Poisson distribution with 95% confidence intervals (CI of mean). Statistical significance of the data from cell adhesion experiments was analyzed by unpaired Student *t* test, from animal experiments was analyzed by one-sample Student *t* test (hypothetical value = 1.0). Significant differences are defined by *P* values (two tailed) < 0.05 (*), < 0.01 (**), and < 0.001(***).

### Ethics statement

All animal works associated with pneumococcal infection in mice were conducted in accordance with the principles in the Chinese law on the humane use of animals for scientific experimentations, and approved by the Institutional Animal Care and Use Committee in Tsinghua University with the animal protocol number 14-ZJR1.

## Supporting Information

S1 Table Quantitative assessment of the stability in the colony opacity phenotypes among the ST556 derivatives. (DOCX)

S2 Table Plasmids and bacterial strains used in this study. (DOCX)

S3 Table Primers used in this study. (DOCX)

S4 Table PCR amplifications used for pneumococcal mutagenesis in this study. (DOCX)

S1 Fig Detection of DNA rearrangements in the Spn556II locus of TIGR4 by PCR. Positions of the primers (**A**) used for PCR amplification are indicated by small arrows. The Spn556II locus in TIGR4 (**B**) and isogenic mutant TH6500 (TIGR4Δ*hsdS*_*A*_::JC)(**C**) were amplified with primers as indicated at the top of each lane. The PCR products that were absent in the mutant strain are marked with asterisks (*). The sizes of the DNA markers are indicated in kilobases.
(TIF)

S2 Fig Requirement of *psrA* for the 15-bp IR1-mediated DNA inversion in TIGR4. The isogenic *psrA* mutant (TH6555) was constructed by counter selection (**A**). The Spn556II *hsdS* region in TIGR4 (upper panel), TH6555 (middle panel), or *psrA* complemented strain TH6669 (lower panel) were amplified with primer pairs indicated at the top of each lane (**B**). The major band absent in TH6555 is marked with an asterisk (*).
(TIF)

S3 Fig Restriction activities of the *hsdS*_*A*_ allelic variants. Transformation frequency of ST556 derivatives carrying wild type *hsdS*_*A*_ locus (**A**), invariable *hsdS*_*A1*_ (**B**), *hsdS*_*A2*_ (**C**), or *hsdS*_*A3*_ (**D**) using pIB166 carrying the methylation motifs of HsdS_A1_ (pTH7223), HsdS_A2_ (pTH7224), or HsdS_A3_ (pTH7225). pIB166 without the methylation motifs (none)(pTH7222) was used as a negative control.
(TIF)

S4 Fig Colony opacity of pneumococcal strains with the intact *cod* locus. The colonies produced by the wild type ST556 and ST606 (*rpsL1*) were observed as described in Fig 6. Strain and the genotype are indicated at the top of each photograph. The “opaque” and “transparent” represent the phenotypes of the seeding colonies.
(TIF)

S5 Fig Strict correlation between the *hsdS*_*A1*_ allele and opaque colony phenotype. The colonies formed by the nine *hsdS*_*A*_ allelic variants of ST556 were observed as described in Fig 6. The strain and *hsdS*_*A*_ genotype are marked at the top of each column.
(TIF)

S6 Fig Epigenetic-driven morphological dynamics of the pneumococcal colonies in the ST556 derivatives carrying either the type-19F (WT) or type-2 (ST556^cps2^) capsule. The *hsdS*_*A*_ allele-locked derivatives of strain ST556 (WT, type 19F) and isogenic capsule switch variant producing a type-2 capsule (ST556^cps2^) were grown for 16 or 24 hours and processed as described in Fig 7. The *hsdS*_*A*_ allele carried by each strain is marked at the left side of each row. The representative colonies with opaque and transparent appearance in the parental strains are indicated with blue and red arrowheads, respectively.
(TIF)

S7 Fig Colony opacity in encapsulated and unencapsulated TIGR4 derivatives. The *hsdS*_*A*_ allele-locked derivatives and parental strain of encapsulated and unencapsulated TIGR4 were grown for 16 or 24 hours and photographed as described in Fig 7. The *hsdS*_*A*_ allele of each strain is marked at the left side of each row. The opaque and transparent colonies in the TIGR4∆*cps* strain are indicated with red and blue arrowheads, respectively.
(TIF)

S8 Fig Functionally validating DNA methyltransferase activity of the wild type HsdM and its allelic mutants in *E*. *coli* by DNA methylation protection assay. **(A)** Schematic map of the pRRS derivatives containing methylation motif 5’-CAAAAAAAAGTACTT-3’ and *hsdM* ^E228A^ *-hsdS*_*A*_ (pTH8221) or *hsdM* ^N255A^ *-hsdS*_*A*_ (pTH8222) that were propagated in *E*. *coli* ER2796. **(B)** The plasmids were treated in the presence (+) or absence (−) of restriction enzymes (PstI and ScaI), and separated by agarose gel electrophoresis. The constructs containing the frame-shifted *hsdM-hsdS*_*A*_ (pTH4836) and wild type *hsdM-hsdS*_*A*_ (pTH4832) were included as the controls for unmethylated DNA and methylated DNA, respectively. Molecular sizes of the standards are indicated in kilobases (kb).
(TIF)

S9 Fig Complementation of the *hsd*_*A1*_∆*hsdRM* mutant of ST556 with the wild type or mutant alleles of *hsdM*. Strain TH7928 lacking the entire coding region of *hsdR* (MYY572) and *hsdM* (MYY571) was complemented with either the wild type *hsdM* or its mutant alleles with a point mutation in catalytic residue E228A or N255A. Both of these two residues are essential for *hsdM* in DNA methylation activity. Only the wild type *hsdM* (strain TH8199) restored the opaque colony phenotype of the *hsd*_*A1*_ allele.
(TIF)
