# Supplementary material for: Epigenetic Switch Driven by DNA Inversions Dictates Phase Variation in Streptococcus pneumoniae
Source: PLoS Pathog. 2016 Jul 18;12(7):e1005762. doi: 10.1371/journal.ppat.1005762 (PMC4948785; doi:10.1371/journal.ppat.1005762)
Supplement: S1 Table — (DOCX) [file ppat.1005762.s001.docx]

**Table S1. Quantitative assessment of the stability in the colony opacity phenotypes among the ST556 derivatives^1^**

| **Strain**  **(genotype)** | **Opacity phenotype of seeding colony** | **No. of colonies** | | **Percentage of the opaque^2^** |
| --- | --- | --- | --- | --- |
|  |  | **Opaque** | **Transparent** |  |
| ST556  (Wild type) | Opaque | 230 | 44 | 83±1 |
|  |  | 200 | 43 |  |
|  |  | 201 | 38 |  |
|  | Transparent | 180 | 31 | 80±6 |
|  |  | 164 | 58 |  |
|  |  | 213 | 54 |  |
| ST606  (ST556, *rpsL1*) | Opaque | 229 | 37 | 83±3 |
|  |  | 225 | 43 |  |
|  |  | 180 | 45 |  |
|  | Transparent | 123 | 32 | 79±5 |
|  |  | 130 | 46 |  |
|  |  | 161 | 32 |  |
| TH6012  (ST606, ∆*psrA*) | Opaque | 276 | 0 | 100 |
|  |  | 219 | 0 |  |
|  |  | 208 | 0 |  |
|  | Transparent | 164 | 1 | 99±1 |
|  |  | 175 | 2 |  |
|  |  | 190 | 6 |  |
| TH5445  (ST606, ∆*hsdS_A_-_C_*::*hsdS_A1_*) | Opaque | 198 | 0 | 100 |
|  |  | 204 | 0 |  |
|  |  | 249 | 0 |  |
| TH5446  (ST606, ∆*hsdS_A_-_C_*::*hsdS_A2_*) | Transparent | 0 | 203 | 0 |
|  |  | 0 | 178 |  |
|  |  | 0 | 219 |  |
| TH5447  (ST606, ∆*hsdS_A_-_C_*::*hsdS_A3_*) | Transparent | 1 | 253 | 0 |
|  |  | 0 | 158 |  |
|  |  | 0 | 176 |  |
| TH5448  (ST606, ∆*hsdS_A_-_C_*::*hsdS_A4_*) | Transparent | 1 | 140 | 0 |
|  |  | 0 | 199 |  |
|  |  | 1 | 213 |  |
| TH5449  (ST606, ∆*hsdS_A_-_C_*::*hsdS_A5_*) | Transparent | 0 | 187 | 0 |
|  |  | 0 | 145 |  |
|  |  | 0 | 227 |  |

| TH5450  (ST606, ∆*hsdS_A_-_C_*::*hsdS_A6_*) | Transparent | 0 | 250 | 0 |
| --- | --- | --- | --- | --- |
|  |  | 0 | 250 |  |
|  |  | 0 | 162 |  |
| TH5451  (ST606, ∆*hsdS_A_-_C_*::*hsdS_A7_*) | Transparent | 1 | 188 | 0 |
|  |  | 0 | 234 |  |
|  |  | 0 | 221 |  |
| TH5452  (ST606, ∆*hsdS_A_-_C_*::*hsdS_A8_*) | Transparent | 1 | 217 | 0 |
|  |  | 1 | 208 |  |
|  |  | 0 | 223 |  |
| TH5453  (ST606, ∆*hsdS_A_-_C_*::*hsdS_A9_*) | Transparent | 0 | 232 | 0 |
|  |  | 0 | 235 |  |
|  |  | 0 | 166 |  |
| TH5792  (ST606, ∆*hsdS_A_-_C_*) | Transparent | 1 | 44 | 0 |
|  |  | 0 | 204 |  |
|  |  | 1 | 244 |  |

^1^Three different colonies were used to streak three agar plates for each phenotype of a single strain; the opaque and transparent colonies on each plate enumerated as described in Fig. 6.

^2^The averages ± standard deviations were calculated on the basis of the three duplicate plates.
