# Supplementary material for: Epigenetic Switch Driven by DNA Inversions Dictates Phase Variation in Streptococcus pneumoniae
Source: PLoS Pathog. 2016 Jul 18;12(7):e1005762. doi: 10.1371/journal.ppat.1005762 (PMC4948785; doi:10.1371/journal.ppat.1005762)
Supplement: S2 Table — (DOCX) [file ppat.1005762.s002.docx]

**Table S2. Plasmids and bacterial strains used in this study**

| **Plasmid** | **Description** | **Reference or source** |
| --- | --- | --- |
| pIB166 | *E. coli* – *S. pneumoniae* shuttle vector, Cm^r^ | (1) |
| pRRS | A vector for expressing methyltransferase genes; Ap^r^ | (2) |
| pTH4832 | pRRS::*hsdM-hsdS* (MYY571-MYY570); coding sequence of MYY571 and MYY570 cloned in PstI/BamHI sites of pRRS followed by 5’-AAGTACTTTTTTTTG-3’; Ap^r^ | (3) |
| pTH4836 | pRRS::frame-shifted *hsdM-hsdS* (MYY571-MYY570) followed by 5’-AAGTACTTTTTTTTG-3’; an adenine was inserted in the site following the start codon of MYY571; Ap^r^ | (3) |
| pTH7222 | pIB166::500 bp DNA fragment without any of 5’-CRAAN_8_CTT-3’, 5’-CRAAN_9_TTC-3’, or 5’-CRAAN_8_CTG-3’ methylation motifs, Cm^r^ | This study |
| pTH7223 | pIB166::500 bp DNA fragment with five 5’-CRAAN_8_CTT-3’ methylation motifs, Cm^r^ | This study |
| pTH7224 | pIB166::500 bp DNA fragment with five 5’-CRAAN_9_TTC-3’ methylation motifs, Cm^r^ | This study |
| pTH7225 | pIB166::500 bp DNA fragment with five 5’-CRAAN_8_CTG-3’ methylation motifs, Cm^r^ | This study |
| PTH8221 | pRRS::*hsdM*^E228A^*-hsdS*; coding sequence of *hsdM*^E228A^*-hsdS* cloned in PstI/BamHI sites of pRRS followed by 5’-AAGTACTTTTTTTTG-3’; Ap^r^ | This study |
| pTH8222 | pRRS::*hsdM^N255A^-hsdS*; coding sequence of *hsdM*^N255A^*-hsdS* cloned in PstI/BamHI sites of pRRS followed by 5’-AAGTACTTTTTTTTG-3’; Ap^r^ | This study |
| **Strain** | **Description** | **Reference or source** |
| D39 | *Streptococcus pneumoniae* strain, serotype 2, encapsulated | (4) |
| ER2796 | *E. coli* derivative, lacking endogenous methyltransferase activities | (2) |
| P384 | *Streptococcus pneumoniae* strain, serotype 6A, encapsulated | (5) |
| ST1759 | TIGR4 derivative; *rpsL1* | (6) |
| ST556 | *Streptococcus pneumoniae* strain, serotype 19F, encapsulated | (7) |
| ST606 | ST556 derivative; *rpsL1* | This study |
| ST877 | *Streptococcus pneumoniae* strain, serotype 35B, encapsulated | This study |
| TH2835 | *Streptococcus pneumoniae* strain, serotype 14, encapsulated | This study |
| TH2886 | *Streptococcus pneumoniae* strain, serotype 23F, encapsulated | This study |
| TH2901 | *Streptococcus pneumoniae* strain, serotype 6B, encapsulated | This study |
| TH3592 | *E. coli* DH5α derivative, carrying pIB166 | This study |
| TH4306 | D39 derivative; *rpsL1* | (8) |
| TH4992 | ST606 derivative; the Spn556II RM system is replaced by JC; ST606*∆*Spn556II::JC | This study |
| TH5444 | TH4992 derivative; the Spn556II RM system is removed; TH4992∆Spn556II; | This study |
| TH5445 | TH5993 derivative; the entire *hsdS* region is replaced by *hsdS_A1_*; TH5993∆*hsdS_A_-_C_*::*hsdS_A1_* | This study |
| TH5446 | TH5993 derivative; the entire *hsdS* region is replaced by *hsdS_A2_*; TH5993∆*hsdS_A_-_C_*::*hsdS_A2_* | This study |
| TH5447 | TH5993 derivative; the entire *hsdS* region is replaced by *hsdS_A3_*; TH5993∆*hsdS_A_-_C_*::*hsdS_A3_* | This study |
| TH5448 | TH5993 derivative; the entire *hsdS* region is replaced by *hsdS_A6_*; TH5993∆*hsdS_A_-_C_*::*hsdS_A6_* | This study |
| TH5449 | TH5993 derivative; the entire *hsdS* region is replaced by *hsdS_A5_*; TH5993∆*hsdS_A_-_C_*::*hsdS_A5_* | This study |
| TH5450 | TH5993 derivative; the entire *hsdS* region is replaced by *hsdS_A4_*; TH5993∆*hsdS_A_-_C_*::*hsdS_A4_* | This study |
| TH5451 | TH5993 derivative; the entire *hsdS* region is replaced by *hsdS_A7_*; TH5993∆*hsdS_A_-_C_*::*hsdS_A7_* | This study |
| TH5452 | TH5993 derivative; the entire *hsdS* region is replaced by *hsdS_A8_*; TH5993∆*hsdS_A_-_C_*::*hsdS_A8_* | This study |
| TH5453 | TH5993 derivative; the entire *hsdS* region is replaced by *hsdS_A9_*; TH5993∆*hsdS_A_-_C_*::*hsdS_A9_* | This study |
| TH5791 | ST606 derivative; the entire *hsdS* region is replaced by JC; ST606∆*hsdS_A-C_*::JC | This study |
| TH5792 | TH5791 derivative; the entire *hsdS* region is removed; TH5791∆*hsdS_A-C_* | This study |
| TH5793 | ST1759 derivative; the entire *hsdS* region is replaced by JC; ST1759∆*hsdS_A-C_*::JC | This study |
| TH5794 | TH5793 derivative; the entire *hsdS* region is removed; TH5793∆*hsdS_A-C_* | This study |
| TH5914 | ST606 derivative; *hsdRM* is replaced by JC1; ST606∆*hsdRM*::JC1 | This study |
| TH5915 | ST6112 derivative; *hsdM* is replaced by JC1; TH6112∆*hsdM*::JC1 | This study |
| TH5993 | ST606 derivative; *psrA* region is inserted by JC; ST606∆*psrA*::JC | This study |
| TH6012 | TH5993 derivative; *psrA* region is removed; TH5993∆*psrA* | This study |
| TH6111 | ST606 derivative; *hsdR* is replaced by JC1; ST606∆*hsdR*::JC1 | This study |
| TH6112 | TH6111 derivative; *hsdR* is removed; TH6111∆*hsdR* | This study |
| TH6113 | TH5914 derivative; *hsdRM* is removed; TH5914∆*hsdRM* | This study |
| TH6115 | TH5915 derivative; wild type *hsdM* is complemented; TH5915::*hsdM* | This study |
| TH6116 | TH5915 derivative; *hsdM*^E228A^ is complemented; TH5915::*hsdM*^E228A^ | This study |
| TH6117 | TH5915 derivative; *hsdM*^N255A^ is complemented; TH5915::*hsdM*^N255A^ | This study |
| TH6500 | ST1759 derivative; *hsdS_A_* is replaced by JC; ∆*hsdS_A_*::JC | This study |
| TH6501 | ST606 derivative; *hsdS_A_* is replaced by JC; ST606∆*hsdS_A_*::JC | This study |
| TH6502 | TH6501 derivative; *hsdS_A_* is removed; TH6501∆*hsdS_A_* | This study |
| TH6525 | ST1759 derivative; *psrA* is replaced by JC; ST1759∆*psrA*::JC | This study |
| TH6555 | TH6525 derivative; *psrA* is removed; TH6525∆*psrA* | This study |
| TH6659 | TH5993 derivative; *psrA* is complemented; TH5993::*psrA* | This study |
| TH6669 | TH6525 derivative; *psrA* is complemented; TH6525::*psrA* | This study |
| TH6671 | P384 derivative; *rpsL1* | This study |
| TH6675 | ST877 derivative; *rpsL1* | This study |
| TH7187 | TH6671 derivative; the entire *hsdS* region is replaced by JC1; TH6671∆*hsdS_A_-_C_*::JC1 | This study |
| TH7191 | TH6675 derivative; the entire *hsdS* region is replaced by JC1; TH6675∆*hsdS_A_-_C_*::JC1 | This study |
| TH7193 | TH7187 derivative; the entire *hsdS* region is replaced by *hsdS_A1_*; TH7187∆*hsdS_A_-_C_*::*hsdS_A1_* | This study |
| TH7196 | TH7187 derivative; the entire *hsdS* region is replaced by *hsdS_A2_*; TH7187∆*hsdS_A_-_C_*::*hsdS_A2_* | This study |
| TH7199 | TH7187 derivative; the entire *hsdS* region is replaced by *hsdS_A3_*; TH7187∆*hsdS_A_-_C_*::*hsdS_A3_* | This study |
| TH7211 | TH7191 derivative; the entire *hsdS* region is replaced by *hsdS_A1_*; TH7191∆*hsdS_A_-_C_*::*hsdS_A1_* | This study |
| TH7214 | TH7191 derivative; the entire *hsdS* region is replaced by *hsdS_A2_*; TH7191∆*hsdS_A_-_C_*::*hsdS_A2_* | This study |
| TH7217 | TH7191 derivative; the entire *hsdS* region is replaced by *hsdS_A3_*; TH7191∆*hsdS_A_-_C_*::*hsdS_A3_* | This study |
| TH7222 | ER2796 derivative; ER2796 transformed with pTH7222 | This study |
| TH7223 | ER2796 derivative; ER2796 transformed with pTH7223 | This study |
| TH7224 | ER2796 derivative; ER2796 transformed with pTH7224 | This study |
| TH7225 | ER2796 derivative; ER2796 transformed with pTH7225 | This study |
| TH7434 | TH7454 derivative; the entire *hsdS* region is replaced by *hsdS_A1_*; TH7454∆*hsdS_A_-_C_*::*hsdS_A1_* | This study |
| TH7437 | TH7454 derivative; the entire *hsdS* region is replaced by *hsdS_A2_*; TH7454∆*hsdS_A_-_C_*::*hsdS_A2_* | This study |
| TH7440 | TH7454 derivative; the entire *hsdS* region is replaced by *hsdS_A3_*; TH7454∆*hsdS_A_-_C_*::*hsdS_A3_* | This study |
| TH7443 | TH7457 derivative; the entire *hsdS* region is replaced by *hsdS_A1_*; TH7457∆*hsdS_A_-_C_*::*hsdS_A1_* | This study |
| TH7446 | TH7457 derivative; the entire *hsdS* region is replaced by *hsdS_A2_*; TH7457∆*hsdS_A_-_C_*::*hsdS_A2_* | This study |
| TH7449 | TH7457 derivative; the entire *hsdS* region is replaced by *hsdS_A3_*; TH7457∆*hsdS_A_-_C_*::*hsdS_A3_* | This study |
| TH7454 | ST1759 derivative; the entire *hsdS* region is replaced by JC1; ST1759∆*hsdS_A_-_C_*::JC1 | This study |
| TH7457 | TH4306 derivative; the entire *hsdS* region is replaced by JC1; TH4306∆*hsdS_A_-_C_*::JC1 | This study |
| TH7556 | TH2835 derivative; *rpsL1* | This study |
| TH7560 | TH2886 derivative; *rpsL1* | This study |
| TH7562 | TH2901 derivative; *rpsL1* | This study |
| TH7568 | TH7556 derivative; the entire *hsdS* region is replaced by JC1; TH7556∆*hsdS_A_-_C_*::JC1 | This study |
| TH7572 | TH7560 derivative; the entire *hsdS* region is replaced by JC1; TH7560∆*hsdS_A_-_C_*::JC1 | This study |
| TH7574 | TH7562 derivative; the entire *hsdS* region is replaced by JC1; TH7562∆*hsdS_A_-_C_*::JC1 | This study |
| TH7587 | TH7568 derivative; the entire *hsdS* region is replaced by *hsdS_A1_*; TH7568∆*hsdS_A_-_C_*::*hsdS_A1_* | This study |
| TH7590 | TH7568 derivative; the entire *hsdS* region is replaced by *hsdS_A2_*; TH7568∆*hsdS_A_-_C_*::*hsdS_A2_* | This study |
| TH7593 | TH7568 derivative; the entire *hsdS* region is replaced by *hsdS_A3_*; TH7568∆*hsdS_A_-_C_*::*hsdS_A3_* | This study |
| TH7605 | TH7572 derivative; the entire *hsdS* region is replaced by *hsdS_A1_*; TH7572∆*hsdS_A_-_C_*::*hsdS_A1_* | This study |
| TH7608 | TH7572 derivative; the entire *hsdS* region is replaced by *hsdS_A2_*; TH7572∆*hsdS_A_-_C_*::*hsdS_A2_* | This study |
| TH7611 | TH7572 derivative; the entire *hsdS* region is replaced by *hsdS_A3_*; TH7572∆*hsdS_A_-_C_*::*hsdS_A3_* | This study |
| TH7614 | TH7574 derivative; the entire *hsdS* region is replaced by *hsdS_A1_*; TH7574∆*hsdS_A_-_C_*::*hsdS_A1_* | This study |
| TH7617 | TH7574 derivative; the entire *hsdS* region is replaced by *hsdS_A2_*; TH7574∆*hsdS_A_-_C_*::*hsdS_A2_* | This study |
| TH7620 | TH7574 derivative; the entire *hsdS* region is replaced by *hsdS_A3_*; TH7574∆*hsdS_A_-_C_*::*hsdS_A3_* | This study |
| TH7901 | TH4306 derivative; the entire *cps* locus is replaced by JC1; TH4306∆*cps*::JC1 | This study |
| TH7903 | TH7901 derivative; the entire *cps* locus (type-2) is replaced by the entire *cps* locus (type-19F) of ST556; TH8160^cps19F^ | This study |
| TH7908 | TH7903 derivative; the entire *hsdS* region is replaced by JC1; TH7903∆*hsdS_A_-_C_*::JC1 | This study |
| TH7910 | TH7908 derivative; the entire *hsdS* region is replaced by *hsdS_A1_*; TH7908∆*hsdS_A_-_C_*::*hsdS_A1_* | This study |
| TH7912 | TH5445 derivative; *hsdR* is replaced by JC1; TH5445∆*hsdR*::JC1 | This study |
| TH7913 | TH7908 derivative; the entire *hsdS* region is replaced by *hsdS_A2_*; TH7908∆*hsdS_A_-_C_*::*hsdS_A2_* | This study |
| TH7915 | TH7912 derivative; *hsdR* is removed; TH7912∆*hsdR* | This study |
| TH7916 | TH7908 derivative; the entire *hsdS* region is replaced by *hsdS_A3_*; TH7908∆*hsdS_A_-_C_*::*hsdS_A3_* | This study |
| TH7918 | TH7915 derivative; *hsdM* is replaced by JC1; TH7915∆*hsdM*::JC1 | This study |
| TH7919 | TH5445 derivative; the coding sequence of *bgaA* is replaced by JC1; TH5445∆bgaA::JC1 | This study |
| TH7921 | TH5446 derivative; the coding sequence of *bgaA* is replaced by JC1; TH5446∆bgaA::JC1 | This study |
| TH7923 | TH7919 derivative; the coding sequence of *bgaA* is replaced by *hsdS_A1_*; TH7919∆*bgaA*::*hsdS_A1_* | This study |
| TH7925 | TH5445 derivative; *hsdRM* is replaced by JC1; TH5445∆*hsdRM*::JC1 | This study |
| TH7926 | TH7919 derivative; the coding sequence of *bgaA* is replaced by *hsdS_A2_*; TH7919∆*bgaA*::*hsdS_A2_* | This study |
| TH7928 | TH7925 derivative; *hsdRM* is removed; TH7925∆*hsdRM* | This study |
| TH7929 | TH7921 derivative; the coding sequence of *bgaA* is replaced by *hsdS_A1_*; TH7921∆*bgaA*::*hsdS_A1_* | This study |
| TH7932 | TH7921 derivative; the coding sequence of *bgaA* is replaced by *hsdS_A2_*; TH7921∆*bgaA*::*hsdS_A2_* | This study |
| TH8118 | TH8208 derivative; the coding sequence of *bgaA* is replaced by *hsdS_A1_*; TH8208∆*bgaA*::*hsdS_A1_* | This study |
| TH8121 | TH8208 derivative; the coding sequence of *bgaA* is replaced by *hsdS_A3_*; TH8208∆*bgaA*::*hsdS_A3_* | This study |
| TH8145 | TH7919 derivative; the coding sequence of *bgaA* is replaced by *hsdS_A3_*; TH7919∆*bgaA*::*hsdS_A3_* | This study |
| TH8148 | TH7921 derivative; the coding sequence of *bgaA* is replaced by *hsdS_A3_*; TH7921∆*bgaA*::*hsdS_A3_* | This study |
| TH8151 | TH8208 derivative; the coding sequence of *bgaA* is replaced by *hsdS_A2_*; TH8208∆*bgaA*::*hsdS_A2_* | This study |
| TH8154 | TH7908 derivative; the entire *hsdS* region is replaced by *hsdS_A4_*; TH7908∆*hsdS_A_-_C_*::*hsdS_A4_* | This study |
| TH8156 | TH7908 derivative; the entire *hsdS* region is replaced by *hsdS_A5_*; TH7908∆*hsdS_A_-_C_*::*hsdS_A5_* | This study |
| TH8158 | TH7908 derivative; the entire *hsdS* region is replaced by *hsdS_A6_*; TH7908∆*hsdS_A_-_C_*::*hsdS_A6_* | This study |
| TH8160 | ST606 derivative; the entire *cps* locus is replaced by JC1; ST606∆*cps*::JC1 | This study |
| TH8162 | TH8160 derivative; the entire *cps* locus (type-19F) is replaced by the entire *cps* locus (type-2) of D39; TH8160^cps2^ | This study |
| TH8164 | TH8162 derivative; the entire *hsdS* region is replaced by JC1; TH8162∆*hsdS_A_-_C_*::JC1 | This study |
| TH8166 | TH8164 derivative; the entire *hsdS* region is replaced by *hsdS_A1_*; TH8164∆*hsdS_A_-_C_*::*hsdS_A1_* | This study |
| TH8168 | TH8164 derivative; the entire *hsdS* region is replaced by *hsdS_A2_*; TH8164∆*hsdS_A_-_C_*::*hsdS_A2_* | This study |
| TH8170 | TH8164 derivative; the entire *hsdS* region is replaced by *hsdS_A3_*; TH8164∆*hsdS_A_-_C_*::*hsdS_A3_* | This study |
| TH8172 | TH8164 derivative; the entire *hsdS* region is replaced by *hsdS_A4_*; TH8164∆*hsdS_A_-_C_*::*hsdS_A4_* | This study |
| TH8174 | TH8164 derivative; the entire *hsdS* region is replaced by *hsdS_A5_*; TH8164∆*hsdS_A_-_C_*::*hsdS_A5_* | This study |
| TH8176 | TH8164 derivative; the entire *hsdS* region is replaced by *hsdS_A6_*; TH8164∆*hsdS_A_-_C_*::*hsdS_A6_* | This study |
| TH8178 | TH5445 derivative; the entire *cps* locus is replaced by JC1; TH5445∆*cps*::JC1 | This study |
| TH8180 | TH5446 derivative; the entire *cps* locus is replaced by JC1; TH5446∆*cps*::JC1 | This study |
| TH8182 | TH5447 derivative; the entire *cps* locus is replaced by JC1; TH5447∆*cps*::JC1 | This study |
| TH8184 | TH7443 derivative; the entire *cps* locus is replaced by JC1; TH7443∆*cps*::JC1 | This study |
| TH8186 | TH7446 derivative; the entire *cps* locus is replaced by JC1; TH7446∆*cps*::JC1 | This study |
| TH8188 | TH7449 derivative; the entire *cps* locus is replaced by JC1; TH7449∆*cps*::JC1 | This study |
| TH8190 | ST1759 derivative; the entire *cps* locus is replaced by JC1; ST1759∆*cps*::JC1 | This study |
| TH8192 | TH7434 derivative; the entire *cps* locus is replaced by JC1; TH7434∆*cps*::JC1 | This study |
| TH8194 | TH7437 derivative; the entire *cps* locus is replaced by JC1; TH7437∆*cps*::JC1 | This study |
| TH8196 | TH7440 derivative; the entire *cps* locus is replaced by JC1; TH7440∆*cps*::JC1 | This study |
| TH8199 | TH7918 derivative; wild type *hsdM* is complemented; TH7918::*hsdM* | This study |
| TH8200 | TH7918 derivative; *hsdM*^E228A^ is complemented; TH7918::*hsdM*^E228A^ | This study |
| TH8201 | TH7918 derivative; *hsdM*^N255A^ is complemented; TH7918::*hsdM*^N255A^ | This study |
| TH8208 | TH5447 derivative; the coding sequence of *bgaA* is replaced by JC1; TH5447∆bgaA::JC1 | This study |
| TH8210 | TH7457 derivative; the entire *hsdS* region is replaced by *hsdS_A4_*; TH7457∆*hsdS_A_-_C_*::*hsdS_A4_* | This study |
| TH8212 | TH7457 derivative; the entire *hsdS* region is replaced by *hsdS_A5_*; TH7457∆*hsdS_A_-_C_*::*hsdS_A5_* | This study |
| TH8214 | TH7457 derivative; the entire *hsdS* region is replaced by *hsdS_A6_*; TH7457∆*hsdS_A_-_C_*::*hsdS_A6_* | This study |

Ap^r^: carrying β lactamase gene; Cm^r^: carrying chloramphenicol acetyltransferase gene.

**References**

1. Biswas I, Jha JK, Fromm N. Shuttle expression plasmids for genetic studies in Streptococcus mutans. Microbiology. 2008;154(Pt 8):2275-82.

2. Murray IA, Clark TA, Morgan RD, Boitano M, Anton BP, Luong K, et al. The methylomes of six bacteria. Nucleic Acids Res. 2012;40(22):11450-62.

3. Feng Z, Li J, Zhang JR, Zhang X. qDNAmod: a statistical model-based tool to reveal intercellular heterogeneity of DNA modification from SMRT sequencing data. Nucleic acids research. 2014;42(22):13488-99.

4. Lanie JA, Ng WL, Kazmierczak KM, Andrzejewski TM, Davidsen TM, Wayne KJ, et al. Genome sequence of Avery's virulent serotype 2 strain D39 of Streptococcus pneumoniae and comparison with that of unencapsulated laboratory strain R6. J Bacteriol. 2007;189(1):38-51.

5. King SJ, Hippe KR, Gould JM, Bae D, Peterson S, Cline RT, et al. Phase variable desialylation of host proteins that bind to Streptococcus pneumoniae in vivo and protect the airway. Mol Microbiol. 2004;54(1):159-71.

6. Hava DL, Camilli A. Large-scale identification of serotype 4 Streptococcus pneumoniae virulence factors. Mol Microbiol. 2002;45(5):1389-406.

7. Li G, Hu FZ, Yang X, Cui Y, Yang J, Qu F, et al. Complete genome sequence of Streptococcus pneumoniae strain ST556, a multidrug-resistant isolate from an otitis media patient. J Bacteriol. 2012;194(12):3294-5.

8. Wen Z, Sertil O, Cheng Y, Zhang S, Liu X, Wang WC, et al. Sequence elements upstream of the core promoter are necessary for full transcription of the capsule gene operon in Streptococcus pneumoniae strain D39. Infection and immunity. 2015;83(5):1957-72.
