# Supplementary material for: Epigenetic Switch Driven by DNA Inversions Dictates Phase Variation in Streptococcus pneumoniae
Source: PLoS Pathog. 2016 Jul 18;12(7):e1005762. doi: 10.1371/journal.ppat.1005762 (PMC4948785; doi:10.1371/journal.ppat.1005762)
Supplement: S3 Table — (DOCX) [file ppat.1005762.s003.docx]

**Table S3. Primers used in this study**

| **Primer** | **Sequence (5’-3’)** |
| --- | --- |
| P1 (Pr8055) | TGGAAATACTTTGGAGAAAAATGTTCGTGAATATACGGATGA |
| P2 (Pr9397) | CAGAATTATCAAGCGGAAAGAGCAACT |
| P3 (Pr9399) | TTCATATATAGGATAAGAGGTTGTTTCGTCTTTG |
| P3' (Pr9405) | AAATATCTTTTATTTTTATAACAACCCAATTCATAGGTATA |
| P4 (Pr7601) | CAAAGACGAAACAACCTCTTATCCTATATATGAA |
| P4' (Pr7604) | TATACCTATGAATTGGGTTGTTATAAAAATAAAAGATATTT |
| P5 (Pr9400) | TCCCAACTTTCTGGTATTTCACAAGGTAC |
| P6 (Pr9401) | GTACCTTGTGAAATACCAGAAAGTTGGGA |
| P7 (Pr9402) | CGCTTGTTCAGGTGAAAGATATTGTAGATT |
| P8 (Pr9403) | AATCTACAATATCTTTCACCTGAACAAGCG |
| P9 (Pr9404) | TTGAAATAGGTTTATTGTTGATGAGATTGTAGATAAT |
| P10 (Pr7567) | ATTATCTACAATCTCATCAACAATAAACCTATTTCAA |
| P11 (Pr8058) | TAATACTAATATAGAAGAAAGATTGCCTGTGGGTAA |
| Pr387 | TGTTCCCAGCTATTTTTATTCAGA |
| Pr388 | TCTCTTTATCCCCTTTCCTTATGC |
| Pr6929 | GAGACTCGAGATTGAACCCTCCGC |
| Pr6930 | CAAACTCGTACAAACAACAAGACCACCTAA |
| Pr7566 | ATGACACCAGAACAACTTAAAGCAAGTATTCT |
| Pr7567 | ATTATCTACAATCTCATCAACAATAAACCTATTTCAA |
| Pr7601 | CAAAGACGAAACAACCTCTTATCCTATATATGAA |
| Pr7602 | CCATAATCACAAAAATAGCAGGTAGTCAGTTT |
| Pr7603 | ATAAAATCAATTTATTGGAATTTTGGGCAA |
| Pr7651 | CAGGAAACAGCTATGAC |
| Pr7652 | GTAAAACGACGGCCAGT |
| Pr7676 | TGATATTTTGCAGTTGTTGGAGGACAA |
| Pr7677 | TTCGTTTCACAATGGGAAATTGATAATTATT |
| Pr7678 | TTGAAAGATTTTGAATTACATTTGAAGAAAGC |
| Pr7679 | GAGATCTAGACTACTTTTAGTTTAATAAGTTCACTTACACGAGCCC |
| Pr7681 | TTACCAATTAACAATTTTATCTACAATATTTTGTTGTTC |
| Pr7806 | GAGACTCGAGGAGCATGTAGAAATCGGT |
| Pr7836 | AAAACAGCTACTGAACAAAATGTAGATAAAAT |
| Pr7837 | TTCAGTAGCTGTTTTAGTATTTTCAGCTAAACCAGCTTTCTTCAAAT |
| Pr7953 | CTACATCATGACAGCAGAAGAGCGC |
| Pr7954 | GAGAGAGCTCCCACTCCCAAGTATCAGGAATATCATAAGG |
| Pr7955 | TAGAATGCGGAGGGTTCAATTTATGTGCTTCAAGTATATA |
| Pr7956 | TATATACTTGAAGCACATAAATTGAACCCTCCGCATTCTA |
| Pr8047 | CTATACCTCCAACCTTTCAAATGCATCTC |
| Pr8048 | GAGATCTAGAATCCTCCTCACTCATTTCTTTCTTAGACTTTATAA |
| Pr8049 | GAGACTCGAGCAAGCAATTAAAGAATTGGAGCAAGAGC |
| Pr8050 | TGCTTTCTTTTCAATACCAAAAACAGCTG |
| Pr8053 | GAGACTCGAGGATAATGTATTAGCTGATATTTTGCAGTTGTTGG |
| Pr8054 | CCTCACCCACTCCCAAGTATCAGG |
| Pr8056 | GAGATCTAGACATCGCTCTTTGGAGAATACTTGCTTTA |
| Pr8057 | GAGACTCGAGTCAATTTCTTGAGTGCTTCCATCAGC |
| Pr8193 | GCCTTTTTATATACTTGAAGCACATAAGGAACATCAATTTCTTG |
| Pr8194 | CAAGAAATTGATGTTCCTTATGTGCTTCAAGTATATAAAAAGGC |
| Pr8227 | ATCGCTGGTCGTGCTGAAATCTG |
| Pr8228 | GAGATCTAGAACAAGTCCCAATCATTACTGAGCAAGC |
| Pr8229 | GAGACTCGAGTCTAGAGCAAGCCAAGCAGCAGTAGCAG |
| Pr8230 | TCAGTTCCTTCTTACCACAAGACCATCTC |
| Pr8406 | TAAATCAACTTTGGAAATGATTATGTGCTTCAAGTATATA |
| Pr8407 | TATATACTTGAAGCACATAATCATTTCCAAAGTTGATTTA |
| Pr8408 | TTTCTCAACTTTTTGAGTAATTATGTGCTTCAAGTATATA |
| Pr8409 | TATATACTTGAAGCACATAATTACTCAAAAAGTTGAGAAA |
| Pr8410 | ATATTGACGCACTAATTTAGTTATGTGCTTCAAGTATATA |
| Pr8411 | TATATACTTGAAGCACATAACTAAATTAGTGCGTCAATAT |
| Pr8412 | TGGAACAAGTTATCCTGCAATCAATGATTATAATTTTAAT |
| Pr8413 | ATTAAAATTATAATCATTGATTGCAGGATAACTTGTTCCA |
| Pr8414 | ATAATAGATTAAAATTATAATTATGTGCTTCAAGTATATA |
| Pr8415 | TATATACTTGAAGCACATAATTATAATTTTAATCTATTAT |
| Pr8416 | TAATTAGAGATAGAAATTGATTATGTGCTTCAAGTATATA |
| Pr8417 | TATATACTTGAAGCACATAATCAATTTCTATCTCTAATTA |
| Pr8771 | TAGCGGTGTTATGCGGAGATAATCTG |
| Pr8772 | GAGACTCGAGTTCTCCTGAGTTCCCAGCATTTTGA |
| Pr8773 | GAGATCTAGAACTTTGGAGAAAAATGTTCGTGAATATACG |
| Pr8774 | CCATCGCTCTTTGGAGAATACTTGCT |
| Pr8797 | TAGAATGCGGAGGGTTCAATATTTACAAGCAATTAAAGAA |
| Pr8798 | TTCTTTAATTGCTTGTAAATATTGAACCCTCCGCATTCTA |
| Pr8799 | TAGAATGCGGAGGGTTCAATAAGATTGATAATGTATTAGC |
| Pr8800 | GCTAATACATTATCAATCTTATTGAACCCTCCGCATTCTA |
| Pr8815 | TACAGCTGTTTTTGGTATTGCTAAGAAAGCATTTCCTCATCT |
| Pr8816 | AGATGAGGAAATGCTTTCTTAGCAATACCAAAAACAGCTGTA |
| Pr8817 | ACCCTAAAATTGTTCATGGAGCTACTTTGGAGAAAAATGTTC |
| Pr8818 | GAACATTTTTCTCCAAAGTAGCTCCATGAACAATTTTAGGGT |
| Pr8983 | AGTTGTTGGAGGACAAATAATTATGTGCTTCAAGTATATA |
| Pr8984 | TATATACTTGAAGCACATAATTATTTGTCCTCCAACAACT |
| Pr9576 | GAGAAAGCTTCGAATACTGAAGCTTCCAAGGCTAGCAACTGAATACGGG |
| Pr9577 | GAGAAGTACTAAGACTCAGTATTCGCGTCAGTTTAAGGGAAGTGATCGTTACA |
| Pr9578 | GAGAAAGCTTCGAATACACTTCTTTCCAACGCCCTTCAACTACCCTATTCA |
| Pr9579 | GAGAAGTACTGAACACTCAGTATTCGAAAGTTCTTGAAGTTAACGCAGATGCAG |
| Pr9780 | GAGAAAGCTTCGAATACTGAAGCTGCTGGAATATTTACACCATCACAACGAATTAG |
| Pr9781 | GAGAAGTACTCAGACTCAGTATTCGGTCATTATATGATTGCAGTGGCAGGG |
| Pr9785 | GAGAAAGCTTTCGAGGAGAGGCTTGTCACGCGT |
| Pr9786 | GAGAAGTACTATGGGCATGAATGACTGTGGTATGAT |
| Pr9967 | GAGATCTAGACACTTGCAGGTTCGTCATTGGG |
| Pr9968 | GAGACTCGAGTGTTGTGGCTGGTGGATTTATTTTCC |
| Pr9969 | GCTTGCAGATTTGATGGGACACG |
| Pr10014 | GAGACTCGAGCATATTGGATATTCCTGCCACTCATAATG |
| Pr10041 | CAGGTCTTTCTTACAAGAAGGGCGATT |
| Pr10103 | GGAACAGGAGGCTTCTTGACTTCG |
| Pr10104 | GAGATCTAGACCATCATCACCACGAAATATCTCAGT |
| Pr10105 | GAGACTCGAGGGGTTGAAATAGGTTTATTGTTGATGAG |
| Pr10106 | ACCCTTAAAATAGCGATTTCGGTCG |
| Pr10107 | CCATCATCACCACGAAATATCTCAGTTTCCTTTTTATCTCGTTTGATTTTTCC |
| Pr10108 | GGAAAAATCAAACGAGATAAAAAGGAAACTGAGATATTTCGTGGTGATGATGG |
| Pr10109 | CTACAATCTCATCAACAATAAACCTATTTCAACCCCATTATCTATAGG |
| Pr10110 | CCTATAGATAATGGGGTTGAAATAGGTTTATTGTTGATGAGATTGTAG |
| Pr10489 | CAGTTTGAACATATCGGTCTTCAGT |
| Pr10491 | CCGCTCGAGATGTTGTTTGAAAAATAATTTTC |
| Pr10583 | TGAACAAGACCCAAATGATGAATCAGTC |
| Pr10584 | TGCTTAAATCGCCCTTCTTGTAAGAAAG |
| Pr10585 | GGCTTCATTATGAGTGGCAGGAATATC |
| Pr10586 | GTTCGATTTTATCAACGATTCTGGATTGT |
| Pr10598 | GAGAAAGCTTAAGTGATTAGTCAAAGAATGGTGATG |
| Pr10599 | GAGAAGTACTTCAGTCTGACGACCAAGAGAGCCA |
| Pr10706 | AGCCAGCTCTCTCTCGTGGAGAATT |
| Pr10711 | TGTTTTCAAAGGTACGAATTTCATCAAAGT |
| Pr10934 | AGTTGTTGGAGGACAAATAATTCTTTTCATCTCTTCATGA |
| Pr10935 | TCATGAAGAGATGAAAAGAATTATTTGTCCTCCAACAACT |
| Pr11159 | GAGATCTAGACCTTAATAGTGGGAATTTGTAAAGTTAATTGAATT |
| Pr11160 | GCTCTGCGAAACTTGCACTTGTTG |
| Pr11162 | ggtctcGAGAAATCCAATTCAAACTTCGTCAGC |
| Pr11163 | ggtctcTTTCTCATCAACAATAAACCTATTTCAACCC |
| Pr11164 | ggtctcATATGACACCAGAACAACTTAAAGCAAGTATTCT |
| Pr11165 | ggtctcTCATATTGAACCCTCCGCATTCTAAAA |
| Pr11166 | ggtctcGGACTGAACGGCTGATGTAGATATTTTATACTT |
| Pr11167 | ggtctcCAGTCCTAAATAGAAGATAAAGAGTCTGGGACA |
