# Supplementary material for: Epigenetic Switch Driven by DNA Inversions Dictates Phase Variation in Streptococcus pneumoniae
Source: PLoS Pathog. 2016 Jul 18;12(7):e1005762. doi: 10.1371/journal.ppat.1005762 (PMC4948785; doi:10.1371/journal.ppat.1005762)
Supplement: S4 Table — (DOCX) [file ppat.1005762.s004.docx]

**Table S4. PCR amplifications used for pneumococcal mutagenesis in this study**

| **Strain ID** | **Relevant primers** | | | **Template DNA** | **Parent strain** |
| --- | --- | --- | --- | --- | --- |
|  | **Upstream** | **Downstream** | **Fusion PCR** |  |  |
| **ST556 background** | | | | | |
| ST606 | Pr387 | Pr388 |  | ST588 | ST556 |
| TH4992 | Pr6930/Pr6929 | Pr7953/Pr7954 |  | ST556 | ST606 |
| TH5444 | Pr6930/Pr7956 | Pr7955/Pr7953 | Pr6930/Pr7953 | ST556 | TH4992 |
| TH5445 | Pr7566/Pr8407 | Pr8406/Pr7953 | Pr7566/Pr7953 | ST556 | TH5993 |
| TH5446 | Pr7566/Pr8409 | Pr8408/Pr7953 | Pr7566/Pr7953 | ST556 | TH5993 |
| TH5447 | Pr7566/Pr8411 | Pr8410/Pr7953 | Pr7566/Pr7953 | ST556 | TH5993 |
| TH5448 | Pr8055/Pr8413  Pr8412/Pr8407 | Pr8406/Pr7953 | Pr8055/Pr7953 | ST556 | TH5993 |
| TH5449 | Pr8055/Pr8413 Pr8412/Pr8409 | Pr8408/Pr7953 | Pr8055/Pr7953 | ST556 | TH5993 |
| TH5450 | Pr8055/Pr8413  Pr8412/Pr8411 | Pr8410/Pr7953 | Pr8055/Pr7953 | ST556 | TH5993 |
| TH5451 | Pr8055/Pr8415 | Pr8414/Pr7953 | Pr8055/Pr7953 | ST556 | TH5993 |
| TH5452 | Pr8055/Pr8417 | Pr8416/Pr7953 | Pr8055/Pr7953 | ST556 | TH5993 |
| TH5453 | Pr8055/Pr8153 | Pr8154/Pr7953 |  | ST556 | TH5993 |
| TH5791 | Pr8055/Pr8056 | Pr8057/Pr8058 |  | ST556 | ST606 |
| TH5792 | Pr8055/Pr8984 | Pr8983/Pr8058 | Pr8055/Pr8058 | ST556 | TH5791 |
| TH5914 | Pr8047/Pr8048 | Pr8053/Pr8054 |  | ST556 | ST606 |
| TH5915 | Pr8771/Pr8772 | Pr8773/Pr8774 |  | TH6112 | TH6112 |
| TH5993 | Pr7678/Pr7679 | Pr7806/Pr7681 |  | ST556 | ST606 |
| TH6012 | Pr7602/Pr7836 | Pr7837/Pr7603 | Pr7602/Pr7603 | ST556 | TH5993 |
| TH6111 | Pr8047/Pr8048 | Pr8049/Pr8050 |  | ST556 | ST606 |
| TH6112 | Pr8047/Pr8798 | Pr8797/Pr8050 | Pr8047/Pr8050 | ST556 | TH6111 |
| TH6113 | Pr8047/Pr8800 | Pr8799/Pr8054 | Pr8047/Pr8054 | ST556 | TH5914 |
| TH6115 | Pr8771 | Pr8774 |  | TH6111 | TH5915 |
| TH6116 | Pr8771/Pr8816 | Pr8815/Pr8774 | Pr8771/Pr8774 | TH6111 | TH5915 |
| TH6117 | Pr8771/Pr8818 | Pr8817/Pr8774 | Pr8771/Pr8774 | TH6111 | TH5915 |
| TH6501 | Pr8055/Pr9967 | Pr9968/Pr9969 |  | ST556 | ST606 |
| TH6502 | Pr8055/Pr10935 | Pr10934/Pr7601 | Pr8055/Pr7601 | ST556 | TH6501 |
| TH6659 | Pr7602 | Pr7567 |  | ST556 | TH5993 |
| TH7912 | Pr8047/Pr8048 | Pr8049/Pr8050 |  | TH5445 | TH5445 |
| TH7915 | Pr8047/Pr8798 | Pr8797/Pr8050 | Pr8047/Pr8050 | TH5445 | TH7912 |
| TH7918 | Pr8771/Pr8772 | Pr8773/Pr8774 |  | TH7915 | TH7915 |
| TH7919 | Pr8227/Pr8228 | Pr8229/Pr8230 |  | TH5445 | TH5445 |
| TH7921 | Pr8227/Pr8228 | Pr8229/Pr8230 |  | TH7919 | TH5446 |
| TH7923 | Pr8230/Pr11167  Pr11165/Pr11166 | Pr11163/Pr11164  Pr8227/Pr11162 |  | TH5445 | TH7919 |
| TH7925 | Pr8047/Pr8048 | Pr8053/Pr8054 |  | TH5445 | TH5445 |
| TH7926 | Pr8230/Pr11167  Pr11165/Pr11166 | Pr11163/Pr11164  Pr8227/Pr11162 |  | TH5446 | TH7919 |
| TH7928 | Pr8047/Pr8800 | Pr8799/Pr8054 | Pr8047/Pr8054 | TH5445 | TH7925 |
| TH7929 | Pr8230/Pr11167  Pr11165/Pr11166 | Pr11163/Pr11164  Pr8227/Pr11162 |  | TH7923 | TH7921 |
| TH7932 | Pr8230/Pr11167  Pr11165/Pr11166 | Pr11163/Pr11164  Pr8227/Pr11162 |  | TH7926 | TH7921 |
| TH8118 | Pr8230/Pr11167  Pr11165/Pr11166 | Pr11163/Pr11164  Pr8227/Pr11162 |  | TH7923 | TH8208 |
| TH8121 | Pr8230/Pr11167  Pr11165/Pr11166 | Pr11163/Pr11164  Pr8227/Pr11162 |  | TH8145 | TH8208 |
| TH8145 | Pr8230/Pr11167  Pr11165/Pr11166 | Pr11163/Pr11164  Pr8227/Pr11162 |  | TH5447 | TH7919 |
| TH8148 | Pr8230/Pr11167  Pr11165/Pr11166 | Pr11163/Pr11164  Pr8227/Pr11162 |  | TH8145 | TH7921 |
| TH8151 | Pr8230/Pr11167  Pr11165/Pr11166 | Pr11163/Pr11164  Pr8227/Pr11162 |  | TH7926 | TH8208 |
| TH8160 | Pr10489/Pr11159 | Pr10491/Pr11160 |  | ST556 | ST606 |
| TH8162 | Pr10706 | Pr10711 |  | D39 | TH8160 |
| TH8164 | Pr8055 | Pr8058 |  | TH5791 | TH8162 |
| TH8166 | Pr8055 | Pr8058 |  | TH5445 | TH8164 |
| TH8168 | Pr8055 | Pr8058 |  | TH5446 | TH8164 |
| TH8170 | Pr8055 | Pr8058 |  | TH5447 | TH8164 |
| TH8172 | Pr8055 | Pr8058 |  | TH5450 | TH8164 |
| TH8174 | Pr8055 | Pr8058 |  | TH5449 | TH8164 |
| TH8176 | Pr8055 | Pr8058 |  | TH5448 | TH8164 |
| TH8178 | Pr10489/Pr11159 | Pr10491/Pr11160 |  | TH8160 | TH5445 |
| TH8180 | Pr10489/Pr11159 | Pr10491/Pr11160 |  | TH8160 | TH5446 |
| TH8182 | Pr10489/Pr11159 | Pr10491/Pr11160 |  | TH8160 | TH5447 |
| TH8199 | Pr8771 | Pr8774 |  | TH7915 | TH7918 |
| TH8200 | Pr8771/Pr8816 | Pr8815/Pr8774 |  | TH7915 | TH7918 |
| TH8201 | Pr8771/Pr8818 | Pr8817/Pr8774 |  | TH7915 | TH7918 |
| TH8208 | Pr8227/Pr8228 | Pr8229/Pr8230 |  | TH7919 | TH5447 |
| **TIGR4 background** | | | | | |
| ST1759 | Pr387 | Pr388 |  | ST588 | TIGR4 |
| TH5793 | Pr8055/Pr8056 | Pr8057/Pr8058 |  | TIGR4 | ST1759 |
| TH5794 | Pr8055/Pr8984 | Pr8983/Pr8058 | Pr8055/ Pr8058 | TIGR4 | TH5793 |
| TH6500 | Pr8055/Pr9967 | Pr10014/Pr10041 |  | TIGR4 | ST1759 |
| TH6525 | Pr7678/Pr7679 | Pr7806/Pr7681 |  | TIGR4 | ST1759 |
| TH6555 | Pr7602/Pr7836 | Pr7837/Pr7603 | Pr7602/Pr7603 | TIGR4 | TH6525 |
| TH6669 | Pr7602 | Pr7567 |  | TIGR4 | TH6525 |
| TH7434 | Pr10103/Pr10107  Pr10108/Pr10109 | Pr10110/Pr10106 | Pr10103/Pr10106 | TIGR4,  TH5445 | TH7454 |
| TH7437 | Pr10103/Pr10107  Pr10108/Pr10109 | Pr10110/Pr10106 | Pr10103/Pr10106 | TIGR4,  TH5446 | TH7454 |
| TH7440 | Pr10103/Pr10107  Pr10108/Pr10109 | Pr10110/Pr10106 | Pr10103/Pr10106 | TIGR4,  TH5447 | TH7454 |
| TH7454 | Pr10103/Pr10104 | Pr10105/Pr10106 |  | TIGR4 | ST1759 |
| TH8190 | Pr10489/Pr11159 | Pr10491/Pr11160 |  | TIGR4 | ST1759 |
| TH8192 | Pr10489/Pr11159 | Pr10491/Pr11160 |  | TH8190 | TH7434 |
| TH8194 | Pr10489/Pr11159 | Pr10491/Pr11160 |  | TH8190 | TH7437 |
| TH8196 | Pr10489/Pr11159 | Pr10491/Pr11160 |  | TH8190 | TH7440 |
| **D39 background** | | | | | |
| TH4306 | Pr387 | Pr388 |  | ST588 | D39 |
| TH7443 | Pr10103/Pr10107  Pr10108/Pr10109 | Pr10110/Pr10106 | Pr10103/Pr10106 | D39, TH5445 | TH7457 |
| TH7446 | Pr10103/Pr10107  Pr10108/Pr10109 | Pr10110/Pr10106 | Pr10103/Pr10106 | D39,  TH5446 | TH7457 |
| TH7449 | Pr10103/Pr10107  Pr10108/Pr10109 | Pr10110/Pr10106 | Pr10103/Pr10106 | D39,  TH5447 | TH7457 |
| TH7457 | Pr10103/Pr10104 | Pr10105/Pr10106 |  | D39 | TH4306 |
| TH7901 | Pr10489/Pr11159 | Pr10491/Pr11160 |  | D39 | D39 |
| TH7903 | Pr10706 | Pr10711 |  | ST556 | TH7901 |
| TH7908 | Pr10103 | Pr10106 |  | TH7457 | TH7903 |
| TH7910 | Pr10103 | Pr10106 |  | TH7443 | TH7908 |
| TH7913 | Pr10103 | Pr10106 |  | TH7446 | TH7908 |
| TH7916 | Pr10103 | Pr10106 |  | TH7449 | TH7908 |
| TH8154 | Pr10103 | Pr10106 |  | TH8210 | TH7908 |
| TH8156 | Pr10103 | Pr10106 |  | TH8212 | TH7908 |
| TH8158 | Pr10103 | Pr10106 |  | TH8214 | TH7908 |
| TH8184 | Pr10489/Pr11159 | Pr10491/Pr11160 |  | TH7901 | TH7443 |
| TH8186 | Pr10489/Pr11159 | Pr10491/Pr11160 |  | TH7901 | TH7446 |
| TH8188 | Pr10489/Pr11159 | Pr10491/Pr11160 |  | TH7901 | TH7449 |
| TH8210 | Pr10103/Pr10107  Pr10108/Pr10109 | Pr10110/Pr10106 | Pr10103/Pr10106 | D39,  TH5450 | TH7457 |
| TH8212 | Pr10103/Pr10107  Pr10108/Pr10109 | Pr10110/Pr10106 | Pr10103/Pr10106 | D39,  TH5449 | TH7457 |
| TH8214 | Pr10103/Pr10107  Pr10108/Pr10109 | Pr10110/Pr10106 | Pr10103/Pr10106 | D39,  TH5448 | TH7457 |
| **P384 background** | | | | | |
| TH6671 | Pr387 | Pr388 |  | ST588 | P384 |
| TH7187 | Pr10103/Pr10104 | Pr10105/Pr10106 |  | P384 | TH6671 |
| TH7193 | Pr10103/Pr10107  Pr10108/Pr10109 | Pr10110/Pr10106 | Pr10103/Pr10106 | P384 | TH7187 |
| TH7196 | Pr10103/Pr10107  Pr10108/Pr10109 | Pr10110/Pr10106 | Pr10103/Pr10106 | P384 | TH7187 |
| TH7199 | Pr10103/Pr10107  Pr10108/Pr10109 | Pr10110/Pr10106 | Pr10103/Pr10106 | P384 | TH7187 |
| **TH2901 background** | | | | | |
| TH7562 | Pr387 | Pr388 |  | ST588 | TH2901 |
| TH7574 | Pr10103/Pr10104 | Pr10105/Pr10106 |  | TH2901 | TH7562 |
| TH7614 | Pr10103/Pr10107  Pr10108/Pr10109 | Pr10110/Pr10106 | Pr10103/Pr10106 | TH2901 | TH7574 |
| TH7617 | Pr10103/Pr10107  Pr10108/Pr10109 | Pr10110/Pr10106 | Pr10103/Pr10106 | TH2901 | TH7574 |
| TH7620 | Pr10103/Pr10107  Pr10108/Pr10109 | Pr10110/Pr10106 | Pr10103/Pr10106 | TH2901 | TH7574 |
| **TH2835 background** | | | | | |
| TH7556 | Pr387 | Pr388 |  | ST588 | TH2835 |
| TH7568 | Pr10103/Pr10104 | Pr10105/Pr10106 |  | TH2835 | TH7556 |
| TH7587 | Pr10103/Pr10107  Pr10108/Pr10109 | Pr10110/Pr10106 | Pr10103/Pr10106 | TH2835 | TH7568 |
| TH7590 | Pr10103/Pr10107  Pr10108/Pr10109 | Pr10110/Pr10106 | Pr10103/Pr10106 | TH2835 | TH7568 |
| TH7593 | Pr10103/Pr10107  Pr10108/Pr10109 | Pr10110/Pr10106 | Pr10103/Pr10106 | TH2835 | TH7568 |
| **TH2886 background** | | | | | |
| TH7560 | Pr387 | Pr388 |  | ST588 | TH2886 |
| TH7572 | Pr10103/Pr10104 | Pr10105/Pr10106 |  | TH2886 | TH7560 |
| TH7605 | Pr10103/Pr10107  Pr10108/Pr10109 | Pr10110/Pr10106 | Pr10103/Pr10106 | TH2886 | TH7572 |
| TH7608 | Pr10103/Pr10107  Pr10108/Pr10109 | Pr10110/Pr10106 | Pr10103/Pr10106 | TH2886 | TH7572 |
| TH7611 | Pr10103/Pr10107  Pr10108/Pr10109 | Pr10110/Pr10106 | Pr10103/Pr10106 | TH2886 | TH7572 |
| **ST877 background** | | | | | |
| TH6675 | Pr387 | Pr388 |  | ST588 | ST877 |
| TH7191 | Pr10103/Pr10104 | Pr10105/Pr10106 |  | ST877 | TH6675 |
| TH7211 | Pr10103/Pr10107  Pr10108/Pr10109 | Pr10110/Pr10106 | Pr10103/Pr10106 | ST877 | TH7191 |
| TH7214 | Pr10103/Pr10107  Pr10108/Pr10109 | Pr10110/Pr10106 | Pr10103/Pr10106 | ST877 | TH7191 |
| TH7217 | Pr10103/Pr10107  Pr10108/Pr10109 | Pr10110/Pr10106 | Pr10103/Pr10106 | ST877 | TH7191 |
