# Supplementary figures and images for: Epigenetic Switch Driven by DNA Inversions Dictates Phase Variation in Streptococcus pneumoniae
Source: PLoS Pathog. 2016 Jul 18;12(7):e1005762. doi: 10.1371/journal.ppat.1005762 (PMC4948785; doi:10.1371/journal.ppat.1005762)

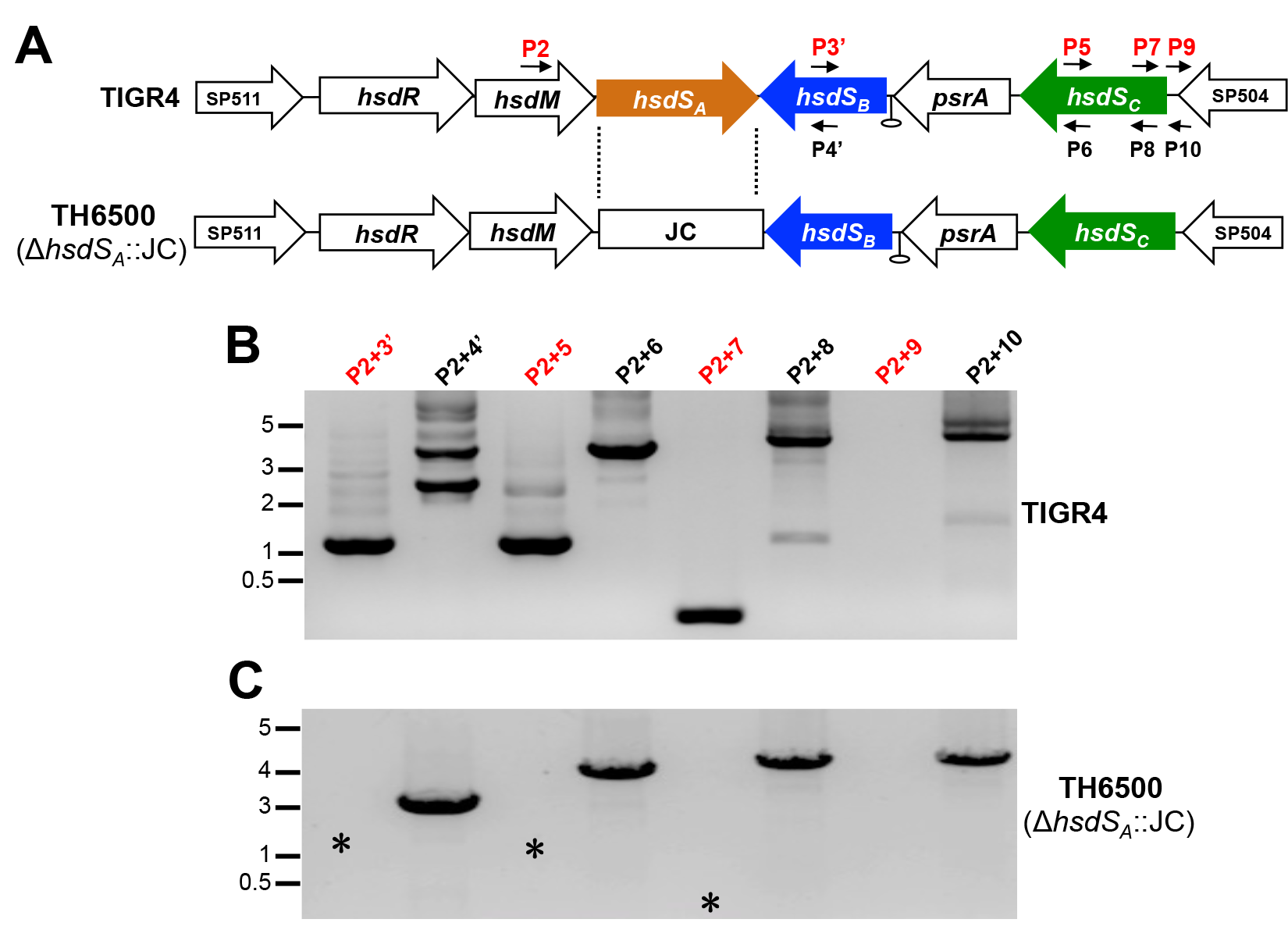

Supplement: S1 Fig — Positions of the primers (A) used for PCR amplification are indicated by small arrows. The Spn556II locus in TIGR4 (B) and isogenic mutant TH6500 (TIGR4ΔhsdS A::JC)(C) were amplified with primers as indicated at the top of each lane. The PCR products that were absent in the mutant strain are marked with asterisks (*). The sizes of the DNA markers are indicated in kilobases. (TIF) [file ppat.1005762.s005.tif]

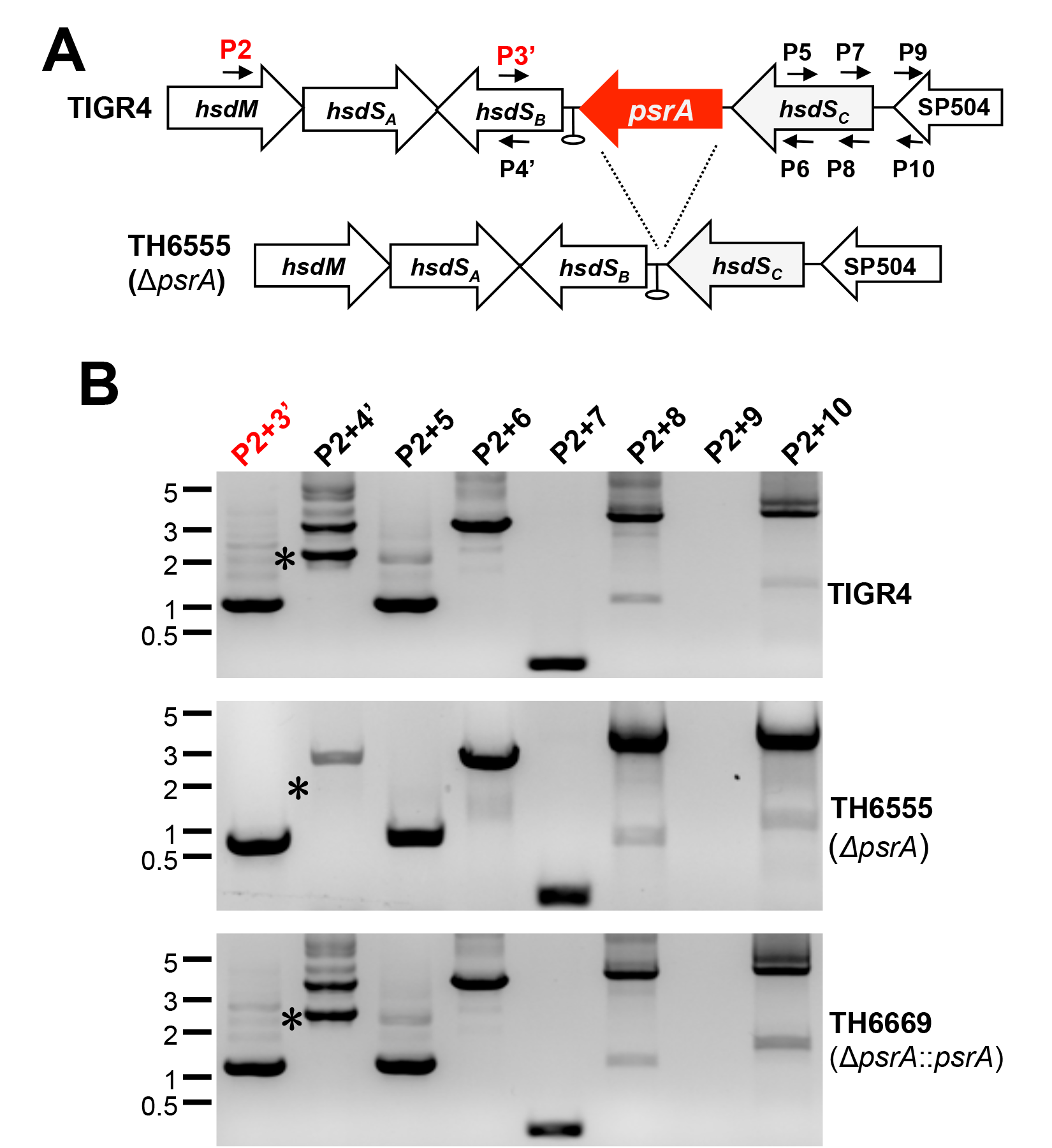

Supplement: S2 Fig — The isogenic psrA mutant (TH6555) was constructed by counter selection (A). The Spn556II hsdS region in TIGR4 (upper panel), TH6555 (middle panel), or psrA complemented strain TH6669 (lower panel) were amplified with primer pairs indicated at the top of each lane (B). The major band absent in TH6555 is marked with an asterisk (*). (TIF) [file ppat.1005762.s006.tif]

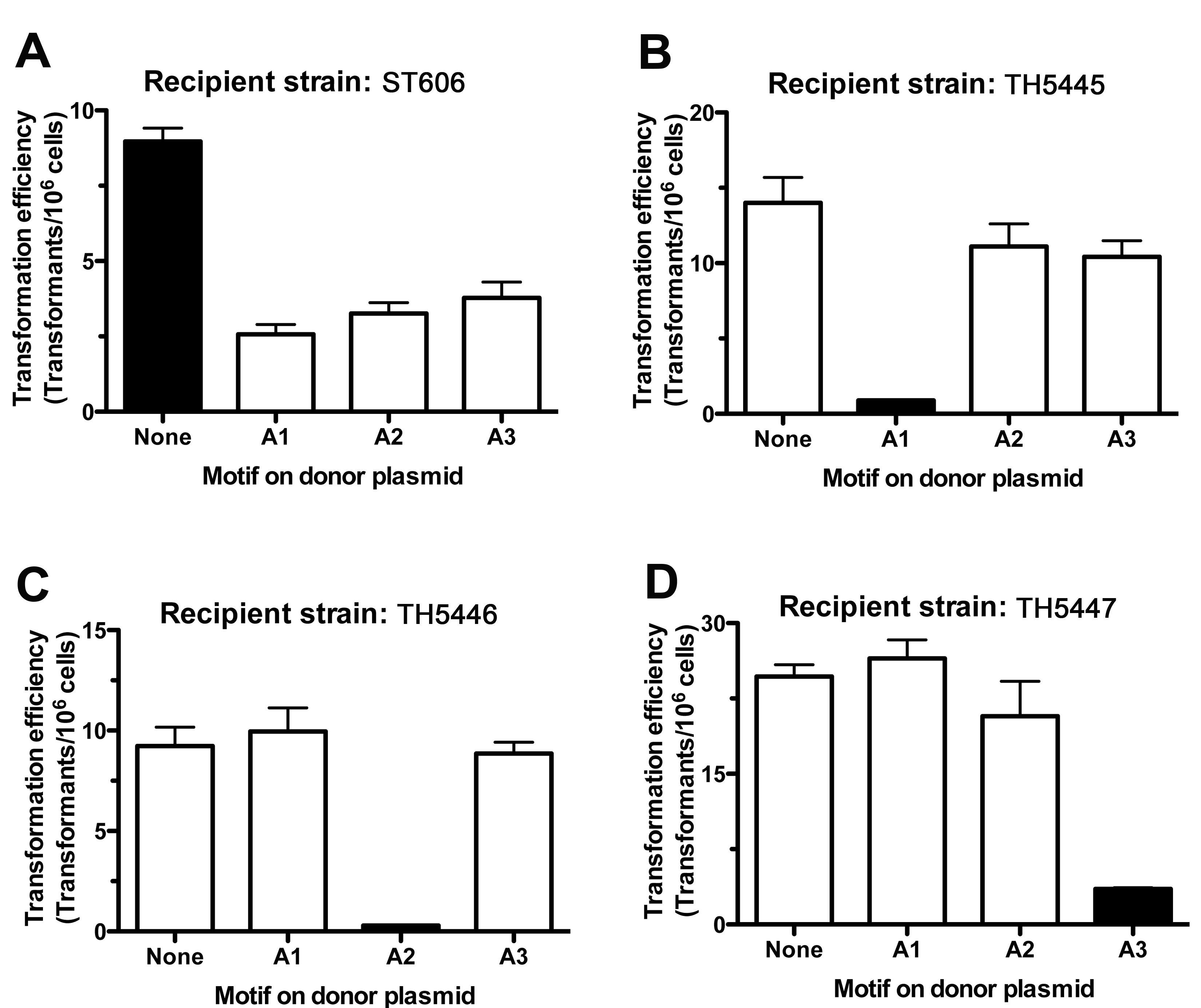

Supplement: S3 Fig — Transformation frequency of ST556 derivatives carrying wild type hsdS A locus (A), invariable hsdS A1 (B), hsdS A2 (C), or hsdS A3 (D) using pIB166 carrying the methylation motifs of HsdSA1 (pTH7223), HsdSA2 (pTH7224), or HsdSA3 (pTH7225). pIB166 without the methylation motifs (none)(pTH7222) was used as a negative control. (TIF) [file ppat.1005762.s007.tif]

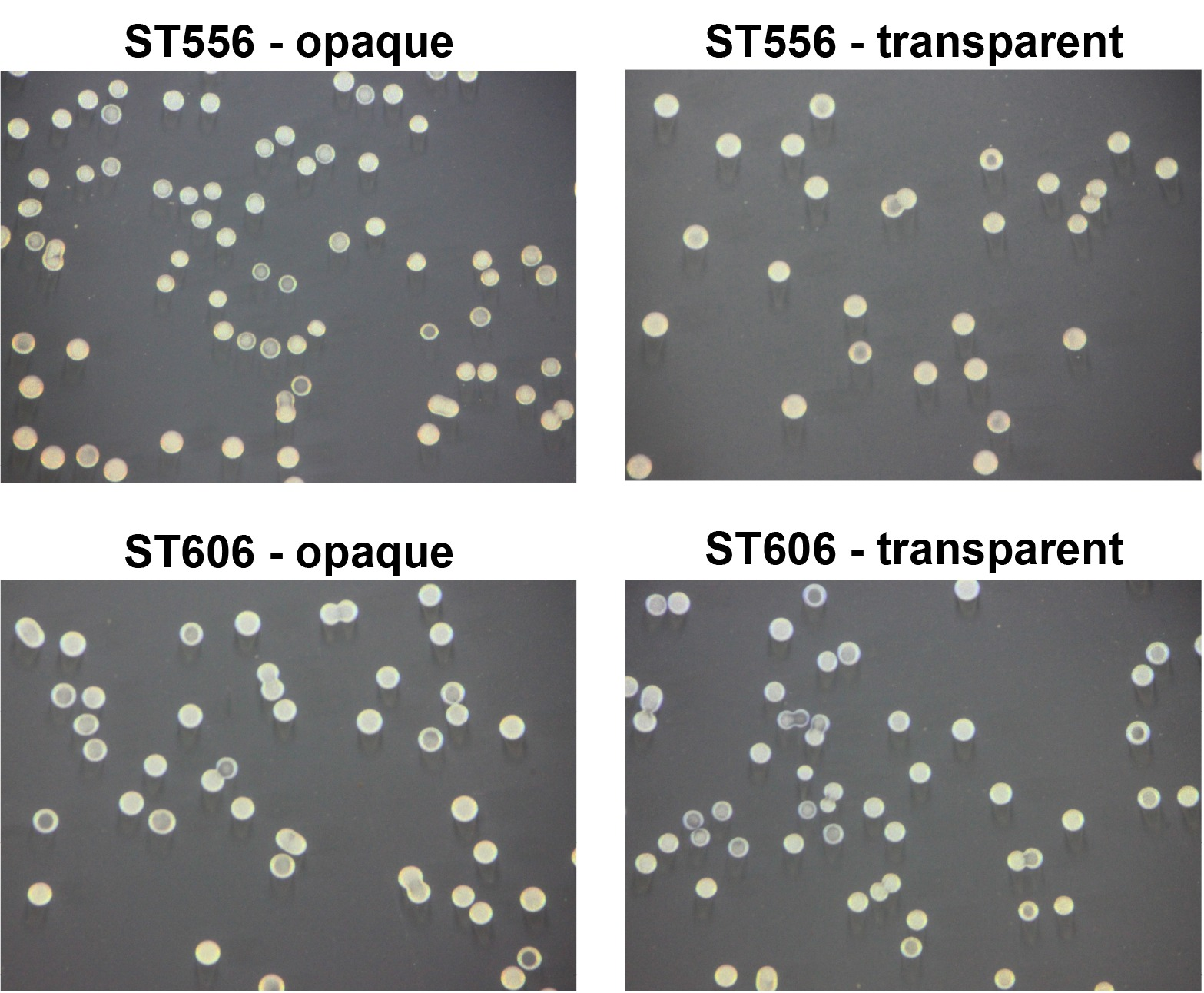

Supplement: S4 Fig — The colonies produced by the wild type ST556 and ST606 (rpsL1) were observed as described in Fig 6. Strain and the genotype are indicated at the top of each photograph. The “opaque” and “transparent” represent the phenotypes of the seeding colonies. (TIF) [file ppat.1005762.s008.tif]

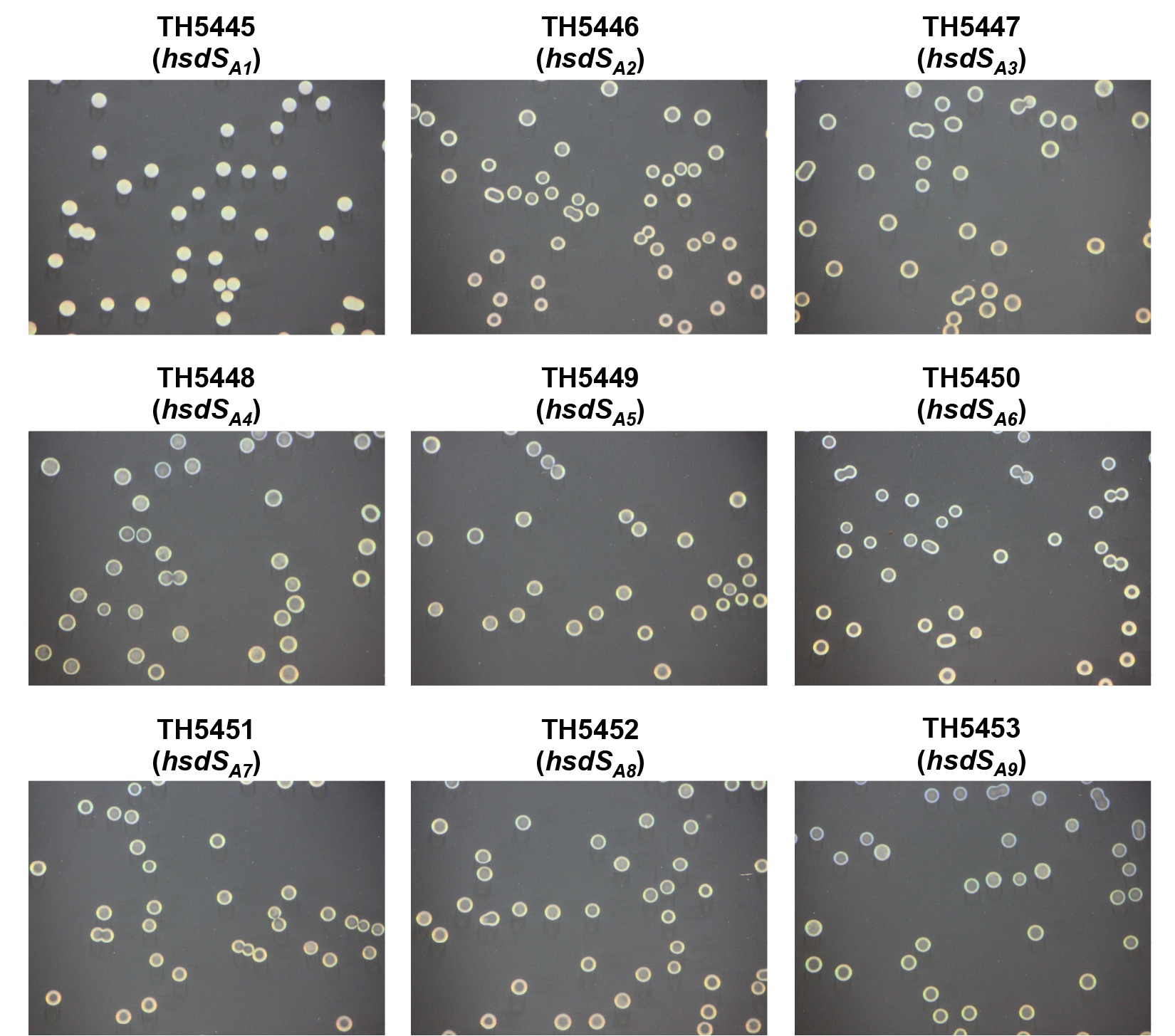

Supplement: S5 Fig — The colonies formed by the nine hsdS A allelic variants of ST556 were observed as described in Fig 6. The strain and hsdS A genotype are marked at the top of each column. (TIF) [file ppat.1005762.s009.tif]

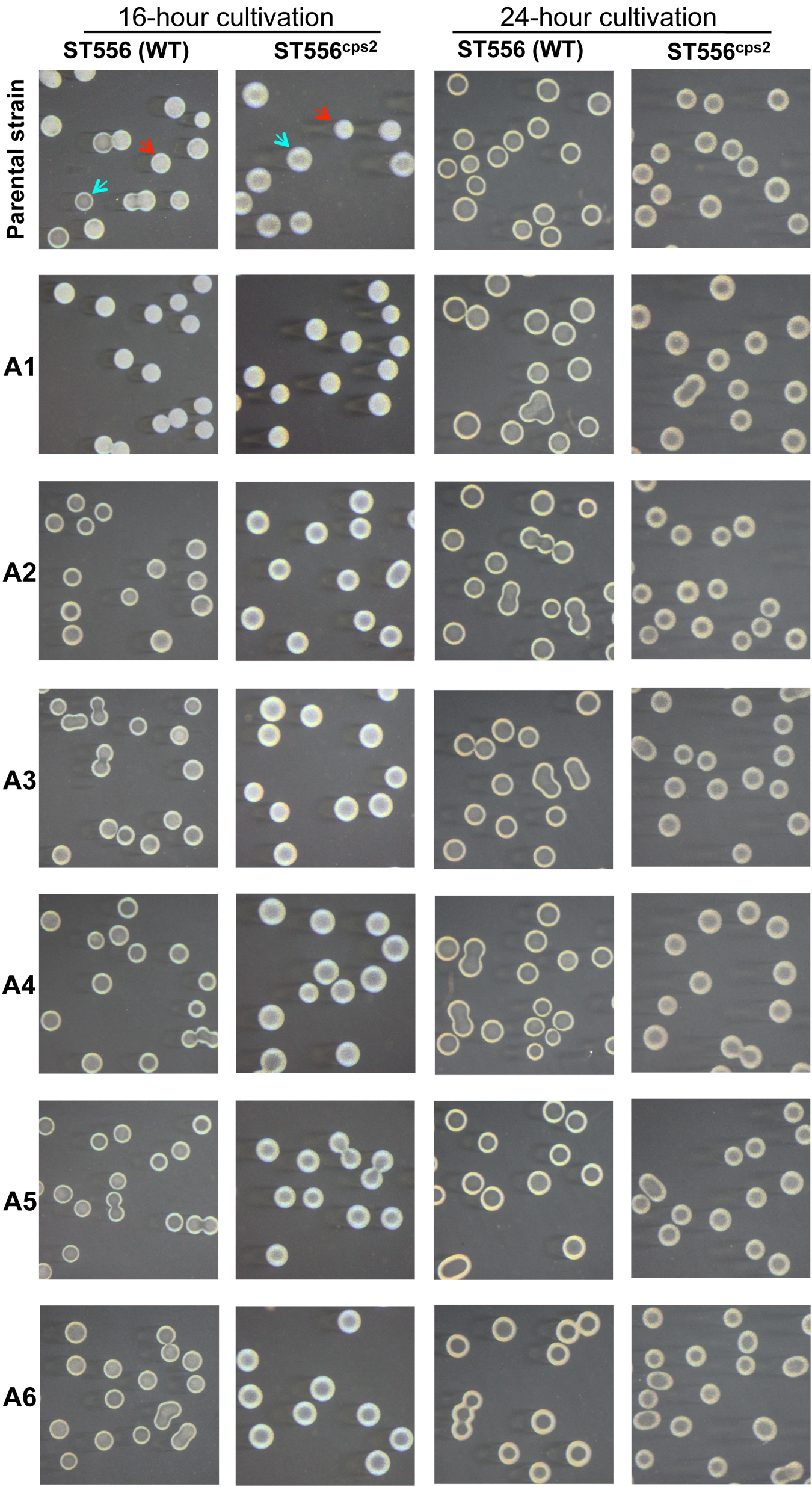

Supplement: S6 Fig — The hsdS A allele-locked derivatives of strain ST556 (WT, type 19F) and isogenic capsule switch variant producing a type-2 capsule (ST556cps2) were grown for 16 or 24 hours and processed as described in Fig 7. The hsdS A allele carried by each strain is marked at the left side of each row. The representative colonies with opaque and transparent appearance in the parental strains are indicated with blue and red arrowheads, respectively. (TIF) [file ppat.1005762.s010.tif]

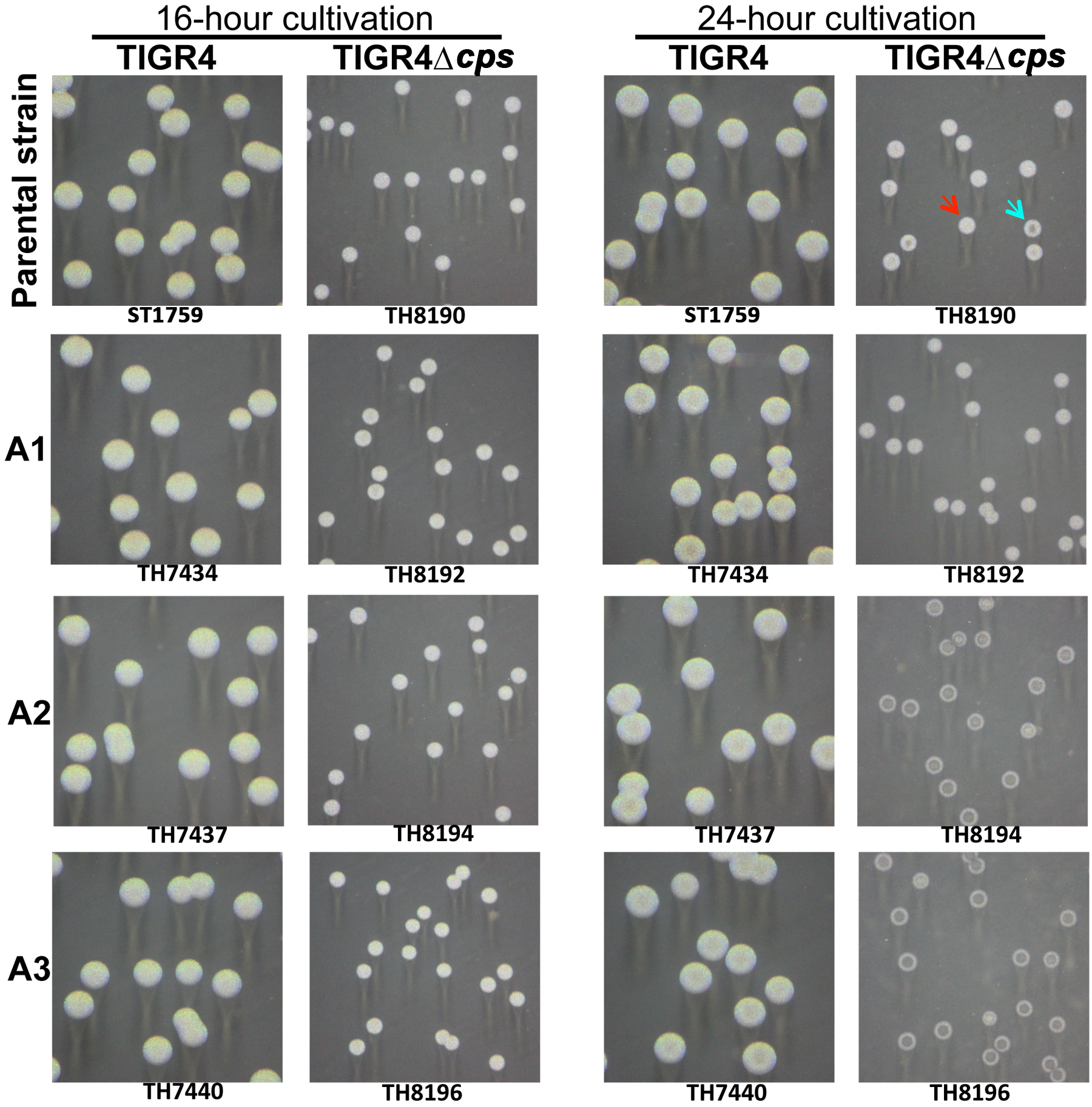

Supplement: S7 Fig — The hsdS A allele-locked derivatives and parental strain of encapsulated and unencapsulated TIGR4 were grown for 16 or 24 hours and photographed as described in Fig 7. The hsdS A allele of each strain is marked at the left side of each row. The opaque and transparent colonies in the TIGR4∆cps strain are indicated with red and blue arrowheads, respectively. (TIF) [file ppat.1005762.s011.tif]

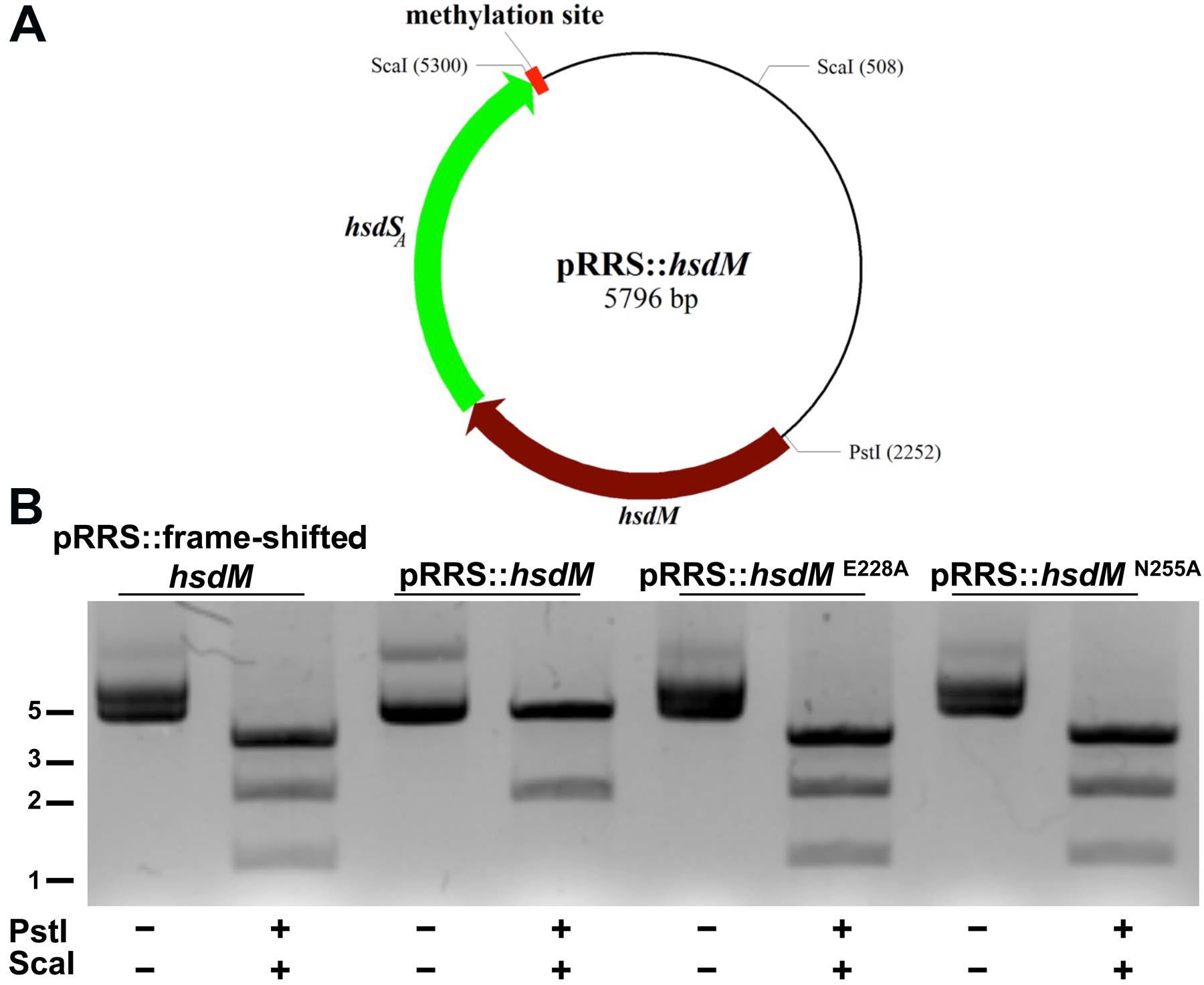

Supplement: S8 Fig — (A) Schematic map of the pRRS derivatives containing methylation motif 5’-CAAAAAAAAGTACTT-3’ and hsdM E228A -hsdS A (pTH8221) or hsdM N255A -hsdS A (pTH8222) that were propagated in E. coli ER2796. (B) The plasmids were treated in the presence (+) or absence (−) of restriction enzymes (PstI and ScaI), and separated by agarose gel electrophoresis. The constructs containing the frame-shifted hsdM-hsdS A (pTH4836) and wild type hsdM-hsdS A (pTH4832) were included as the controls for unmethylated DNA and methylated DNA, respectively. Molecular sizes of the standards are indicated in kilobases (kb). (TIF) [file ppat.1005762.s012.tif]

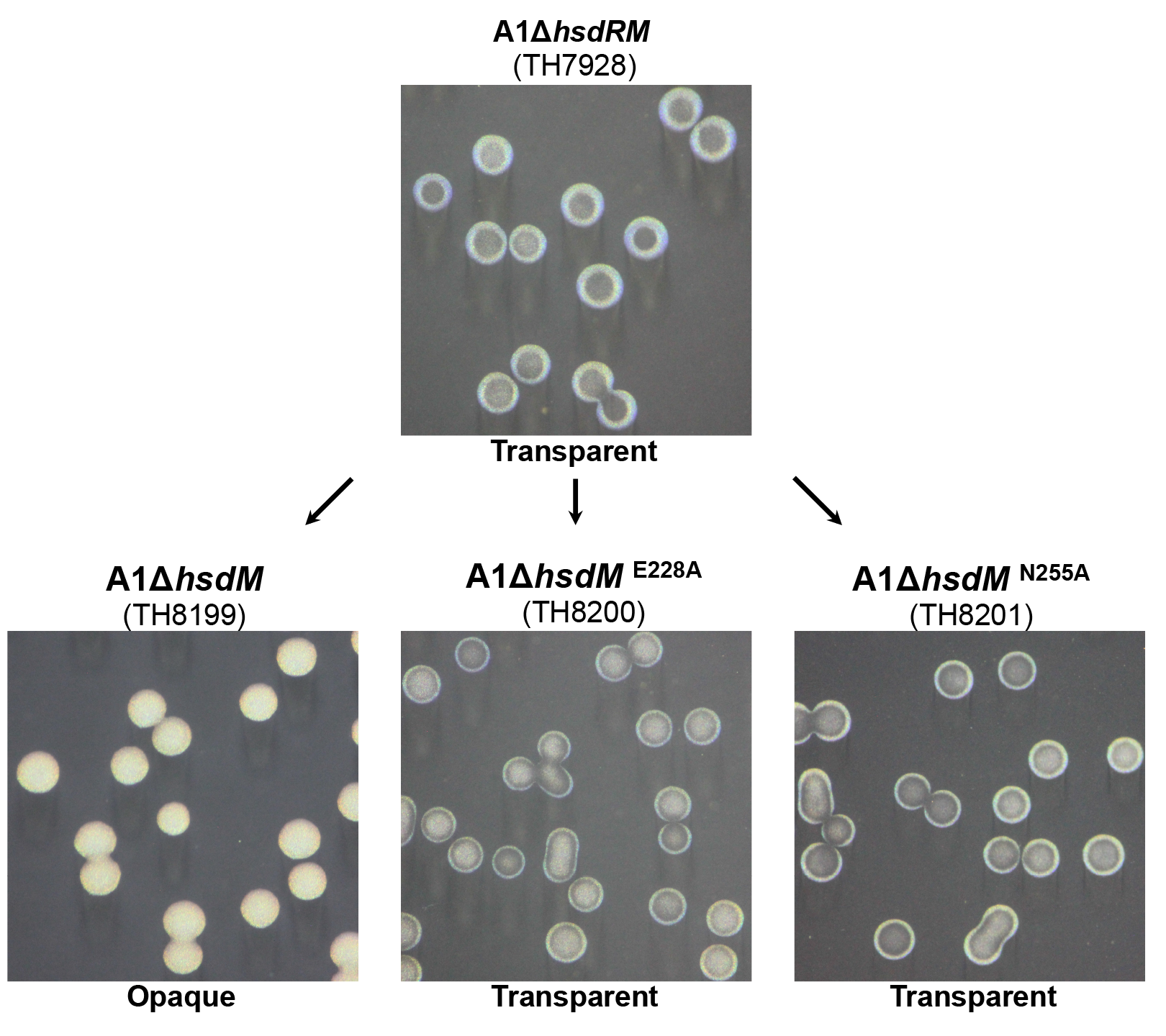

Supplement: S9 Fig — Strain TH7928 lacking the entire coding region of hsdR (MYY572) and hsdM (MYY571) was complemented with either the wild type hsdM or its mutant alleles with a point mutation in catalytic residue E228A or N255A. Both of these two residues are essential for hsdM in DNA methylation activity. Only the wild type hsdM (strain TH8199) restored the opaque colony phenotype of the hsd A1 allele. (TIF) [file ppat.1005762.s013.tif]
